# Book of Abstracts

**DOI:** 10.1080/24740527.2026.2685938

**Published:** 2026-07-16

## Keynote: Cannabis-Based Medicines for Chronic Pain: Updates from Europe

**Professor Winfried Hauser**

Medical Center Pain Medicine and Mental Health Saarbrücken - St. Johann

**Abstract**

The role of cannabis-based medicines (CbMs) for the management of chronic pain is under debate, with conflicting conclusions from systematic reviews and recommendations from medical associations. I will argue that it is necessary to differentiate between different types of CbMs, e.g. THC-dominant, CBD-dominant, THC/CBD-balanced or synthetic cannabinoids versus full spectrum cannabis extracts. Lumping all types of CbMs in a quantitative analysis, as the Neuropathic SPIG of the IASP did recently, underestimates the potential of THC-dominant and THC/CBD-balanced medicines for the management of chronic neuropathic pain. I will justify this statement by a Cochrane review on CbMs for chronic neuropathic pain which will be published in February 2026.

In addition, the significance of systematic reviews of randomised controlled trials of CbMs for chronic pain is limited because most studies included had small sample sizes and a short study duration. I will present two recent European studies with an oral THC-dominant full spectrum cannabis extract in patients with chronic low back pain. One study included 820 patients in a double-blind 12-week treatment phase compared to placebo, a 6-month open-label extension, followed by either a 6-month continuation or randomized withdrawal. The other study included 384 patients and compared during 24 weeks of double - blind treatment against opioids. Based on the findings of the studies, I will discuss the potential role of this medication in the management of chronic low back pain.

Finally, I will give a short overview of the availability of CbMs for the management of chronic pain in European countries and will outline some obstacles for physicians to prescribe CbMs in Germany.

**At the end of this session, participants will be able to**:

- Recognize there are different types of cannabis-based medicines that differ in their efficacy to reduce pain and pain-related symptoms.
- Evaluate reasons for conflicting conclusions of systematic reviews and recommendations from medical associations on cannabis-based medicines for chronic pain.
- Consider the use of cannabis-based medicines based on the dominant mechanism (nociceptive, neuropathic, nociplastic, mixed types) of pain and the non-pharmacological therapies available.

**Plenary**

**Learning and Brain Plasticity in the Development and Persistence of Chronic Pain: Differential Mechanisms an Implications for Patient-Tailored Treatment**

**Professor Herta Flor**

Central Institute of Mental Health, Mannheim

Heidelberg University

**Abstract**

There is ample evidence that learning mechanisms such as sensitization, learning about reward and punishment or emotional learning related to appetitive and aversive stimuli are altered in subacute and chronic pain and predict chronicity. These behavioral mechanisms are related to changes in structure, function and connectivity in many brain regions such as the primary somatosensory cortex, insula, prefrontal, orbitofrontal and striatal networks. We discuss how these mechanisms differ between early and later stages of clinical pain and propose to differentially target these mechanisms in a patient-specific manner using assessment batteries with behavioral and neurobiological biomarkers. We propose modular interventions that involve, for example, sensory or sensorimotor training, brain-based interventions, virtual and augmented reality applications, extinction and exposure training, pharmacologically aided behavioral and cognitive interventions and digital ecological interventions based on individual patient profiles.

**At the end of this session, participants will be able to:**

- Outline learning mechanisms that contribute to pain chronicity.
- Define core brain regions involved in risk for pain chronicity.
- Recognize novel mechanistic interventions for chronic pain.

**Plenary**

**Insights into neuropathic pain mechanisms from human tissue studies**

**Professor Theodore (Ted) Price**

University of Texas

**Abstract**

Dr. Price’s talk will focus on his lab’s and the PRECISION Human Pain Network’s work on human dorsal root ganglion (DRG), peripheral nerves, and spinal cord tissues obtained from either rare surgeries or organ donors. The focus will be on comparing control tissues to those with clear medical histories of chronic neuropathic pain disorders like painful diabetic neuropathy. Dr. Price will present evidence that human nociceptors become hyperexcitable in these conditions, exhibiting spontaneous electrical activity even after days in culture. He will link these physiological findings to -omic and biochemical studies that give insight into the mechanisms that cause these effects in humans and how we might target them with therapeutics. He will also highlight evidence demonstrating that neuropathic pain in humans is often accompanied by neuronal degeneration in the DRG that primarily affects certain kinds of sensory neurons. Collectively, these findings will give the audience a new insight into why patients have neuropathic pain and how new treatments might be able to target the underlying cause of the disease.

**At the end of this session, participants will be able to**:

- Explore factors that may underlie spontaneous activity in nociceptors of humans suffering from neuropathic pain.
- Recognize how non-neuronal cells like satellite glia and adipocytes produce ligands that act on sensory neurons within the the DRG to cause pathology in neuropathic pain.
- Identify that painful diabetic neuropathy is associated with wide-spread neuronal degeneration in the DRG that might be linked to the production of pain.

**Hot Topics Presentations**

**Session Chair: Bradley Kerr**

University of Alberta

**Learning Objectives**

At the end of this session, participants will be able to:

Describe the latest research in pain mechanisms and clinical care; and

Critique and evaluate emerging topics in pain research.

**Pain - Universally Experienced, Unevenly Distributed**

Jax Norman^1^, Matthew Fillingim^1^, Christophe Tanguay-Sabourin^2^, Azin Zare^1^, Lindsay Neuert^1^, Gianluca Guglietti^1^, Etienne Vachon-Presseau^1^

^1^McGill University, ^2^Université de Montréal

**Introduction**: While pain represents a leading cause of global disability, its burden is not equally distributed. Marginalized populations consistently report higher pain prevalence, yet most research remains thematically and geographically siloed. This study leverages data from 10 international cohorts (n=1.28 million) to examine how multiple dimensions of identity, including race/ethnicity, sex/gender, sexuality, caste, immigration status, and rurality, shape pain experiences globally.

**Methods**: This study used a two-stage harmonized consortium approach across 10 cohorts. In stage one, we create weighted logistic regression models within each cohort to examine associations between marginalization status and pain outcomes (general pain and burdensome pain defined as multisite, moderate/severe, or chronic). In stage two, we will use Multilevel Analyses of Individual Heterogeneity and Discriminatory Accuracy (MAIHDA) to examine the intersectional effects of different dimensions of identity.

**Results**: Across cohorts, socially disadvantaged groups consistently demonstrated greater odds of experiencing pain, especially for more burdensome pain phenotypes. For example, in the Longitudinal Aging Study in India (LASI), participants living in rural areas and participants from Scheduled Castes were significantly more likely to report experiencing multisite pain (OR=1.48 [1.37-1.60]; OR=1.14 [1.06-1.23]). Similar results were found in the Chinese Health and Retirement Longitudinal Study (CHARLS), where women and participants from ethnic minority groups were significantly more likely to report experiencing multisite pain (OR=1.83 [1.62-2.06]; OR=1.81 [1.50-2.20]). Analyses of the other cohorts are forthcoming.

**Discussion/Conclusions**: These initial findings support the hypothesis that marginalization, regardless of its specific form, transduces into pain, with intersecting identities potentially compounding the odds of experiencing pain.

**At-home blood collection for proteomic and transcriptomic analysis of chronic pain**

Mara Majer^1^, Oliver Schott^2^, Doriana Taccardi^1^, Hailey Gowdy^1^, Vina Li^1^, Amanda Zacharias^1^, Élisabeth Lamoureux^3^, M. Gabrielle Pagé^3, 4, 5^, Hanno Steen^2^, Nader Ghasemlou^1, 6^

^1^Department of Biomedical and Molecular Sciences, Queen’s University, Kingston, Ontario, Canada, ^2^Department of Pathology, Boston Children’s Hospital and Harvard Medical School, Boston, MA, United States, ^3^Department of Psychology, Université de Montréal, Montreal, Quebec, Canada, ^4^Centre hospitalier de l’Université de Montréal (CHUM) Research Center, Montreal, Quebec, Canada, ^5^Department of Anesthesiology and Pain Medicine, Université de Montréal, Montreal, Quebec, Canada, ^6^Department of Anesthesiology and Perioperative Medicine, Queen’s University, Kingston, Ontario, Canada

**Introduction**: Pain intensity is variable between people and within individuals across the day. Circadian (24-hour) rhythms influence RNA and protein expression in the healthy state and across diseases. Whole blood samples are commonly used to detect disease biomarkers. Our group recently identified circadian rhythmicity in immune cell numbers and whole-blood RNA in people with chronic low back pain (cLBP). Venipuncture was used for this analysis; however, this method is costly and complicates the implementation of repeated sampling protocols. Thus, there is a need for efficient, minimally invasive ways of collecting blood; we used dried blood spots (DBS) as a potential solution.

**Methods**: To investigate whether daily changes in RNA and protein levels can be captured using DBS, two commercially available devices were tested in 23 participants. Collection kits were sent via mail for participants to complete blood collection up to six times across 48 hours, for a total of 251 samples.

**Results**: Liquid chromatography-mass spectrometry was conducted with >1,400 proteins included for analysis. A harmonic regression model identified eight proteins with significant regression coefficients (FDR<0.01), indicating a potential rhythmic expression pattern. RNA extraction shows limited quantity (average total RNA per blood spot = 486 ng, SEM = 47.8) and quality (average A260/280 = 1.52, SEM = 0.02), though we continue optimizing protocols.

**Discussion/Conclusion**: Implementing these blood collection tools in people with cLBP to assess potential rhythmic biomarkers is underway. Our methodological development has the potential to revolutionize pain research by facilitating more efficient collection of biological samples across multiple timepoints.

**Solace, a Therapeutic Conversational Agent for Management of Chronic Pain: Acceptability and Usability Study**

Stephanie Buryk-Iggers^1^, P. Maxwell Slepian^1,2,3,4^, Anna Lomanowska^1^, Binh Nguyen^2^, Tahir Janmohamed^2^, Hance Clarke^1,2,3,4^, Joel Katz^1,2,3,4,5^, Nils Niederstrasser^6^

^1^Department of Anesthesia and Pain Management, Toronto General Hospital, University Health Network, Toronto, ON, ^2^ManagingLife, Inc., Toronto, ON, ^3^Department of Anesthesiology and Pain Medicine, University of Toronto, Toronto, ON, ^4^University of Toronto Centre for the Study of Pain, University of Toronto, Toronto, ON, ^5^Department of Psychology, York University, ^6^School of Psychology, Sport, and Health Sciences, University of Portsmouth

**Background**: Access to efficacious treatment for chronic pain is limited by geography, economics, and scale. Digital health interventions offer an easily scaled solution. Whereas autonomous conversational agents powered by generative artificial intelligence (AI) represent a new frontier in this treatment domain, they have yet to be developed or examined for treatment of chronic pain.

**Objectives**: We sought to develop and test Solace, a first-of-its-kind, expert trained generative AI conversational agent, delivering support grounded in principles of evidence-based pain psychology.

**Methods**: We conducted an acceptability and usability study of Solace in individuals with chronic pain. Participants (n=175) interacted with Solace for 25 minutes. Self-report measures of system usability, treatment acceptability, and therapeutic alliance were completed after the interaction and clinically relevant pain related measures were completed before and after the interaction. Safety guardrails designed to identify and manage instances of suicidal ideation, injury, or requests for medication recommendations performed appropriately.

**Results**: Participants rated the usability of Solace to be excellent (System Usability Scale, mean = 85.04) and that Solace is acceptable as an intervention for chronic pain. Therapeutic alliance between participants and Solace was rated highly (Working Alliance Inventory, mean = 188.03). Participants demonstrated statistically significant improvements in anxiety, pain interference, kinesiophobia, and pain resilience (*p’s* <0.02).

**Discussion/Conclusion**: Solace is a usable and acceptable expert-trained generative AI conversational agent for pain management. Its use is associated with improvements in clinically relevant domains. Randomized clinical trials are needed to evaluate the efficacy of Solace as a strategy for treatment of chronic pain.

**Characterization of IL-1R1+ nociceptors in pain associated with neuroinflammation**

Dominic Bélanger^1, 2^, Camille Illiano^1, 2^, Nicolas Vallières^2^, Nadia Fortin^2^, Steve Lacroix^1, 2^

^1^Département de médecine moléculaire, Faculté de médecine, Université Laval, Québec, QC, Canada, G1V 0A6, ^2^Axe neurosciences, Centre de recherche du CHU de Québec-Université Laval, Québec, QC, Canada, G1V 4G2

**Introduction**: Pain affects about 20% of adults worldwide, and its prevalence exceeds 50% among patients with inflammatory autoimmune diseases such as multiple sclerosis. Painful signals are transmitted from the periphery to the spinal cord and brain via the dorsal root ganglia (DRGs), where nociceptors reside. Interleukin-1β (IL-1β) is a proinflammatory cytokine capable of independently triggering both inflammation and pain, yet the neuronal subtypes mediating its effects remain poorly defined.

**Methods**: Confocal immunofluorescence microscopy was used to characterize the expression of the interleukin-1 receptor type 1 (IL-1R1) in mouse and human DRGs. Spatial transcriptomics and single-cell RNA sequencing (scRNA-Seq) were integrated to define the molecular identity of IL-1R1+ nociceptors. Behavioral analyses were conducted in C57BL/6J mice and IL-1R1-deficient mice following intra cisterna-magna (i.c.m.) administration of IL-1β to assess the contribution of the IL-1β/IL-1R1 signaling pathway to pain.

**Results**: IL-1R1 was found to be highly expressed in a distinct subpopulation of TRPV1+ nociceptors, representing approximately 5-10% of all DRG neurons in both species. Transcriptomic analyses revealed that IL-1R1+ neurons correspond to a subset of non-peptidergic type 3 (NP3) small-diameter, unmyelinated sensory neurons previously associated with inflammation-induced itch. However, deletion of *Il1r1* did not affect itch behaviors triggered by inflammatory mediators such as serotonin, histamine, or chloroquine. In contrast, i.c.m. IL-1β injection selectively upregulated several genes within the NP3 population, many of which are implicated in pain.

**Discussion/Conclusion**: This study identifies IL-1R1+nociceptors as a distinct neuronal subset involved in neuroinflammation-induced pain and reveals novel molecular markers that link IL-1 signaling to pain mechanisms.

**“CARING in Action”: A Knowledge Mobilization Case Report on Communication Training for Chronic Pain Care**

Doriana Taccardi^1^, Nataly R Espinoza Suarez^2^, Swapnil Shah^3^, Jennifer Daly-Cyr^3^, Annie LeBlanc^2, 4, 5^, Nader Ghasemlou^1, 6, 7^, Lynn Cooper^3^, Rachael Bosma^8, 9^, Rachel Roy^3^

^1^Department of Biomedical & Molecular Sciences, Queen’s University, Kingston, Canada, ^2^VITAM Research Center on Sustainable Health, Quebec Integrated University Health and Social Services Center, Québec, ^3^Chronic Pain Network, McMaster University, Ontario, Canada, ^4^Knowledge Evaluation Research Unit, Mayo Clinic, Rochester, Minnesota, USA, ^5^Faculty of Medicine, Laval University, Quebec, Canada, ^6^Department of Anaesthesiology & Perioperative Medicine, Queen’s University, Kingston, Canada, ^7^Centre for Neuroscience Studies, Queen’s University Kingston, Canada, ^8^Centre for the Study of Pain, University of Toronto, ^9^Toronto Academic Pain Medicine Institute, Women’s College Hospital

**Introduction**: Communication gaps between clinicians and people with lived and living experience (PWLLEs) persist as a major barrier to effective, patient-centered care. Supporting the education of clinicians is essential to improving patient satisfaction and the overall experience of care for both clinicians and PWLLEs. The CARING framework was co-created to address this gap in pain management and communication in pain care.

**Methods**: CARING in Action was a collaborative project hosted by the Chronic Pain Network’s (CPN) Training and Capacity Building Committee. This work took place from August 2024 to May 2025 via a “Hack-a-thon” project that brought together approximately 25 participants, including PWLLEs of chronic pain, trainees, clinicians, knowledge brokers, and pain researchers, to form interprofessional teams through iterative, virtual and in-person workshops that emphasized consensus-building, inclusivity, and validation.

**Results**: The co-creation process produced an eight-module educational resource structured around six principles summarized by the acronym CARING: Connect, Ask, Respect, Inform, Nurture, and Generate trust. Each module integrates testimonials, reflective exercises, and practical communication examples. This aligns with the national priorities for chronic pain knowledge mobilization and with the recommendations of the Action Plan for Pain in Canada, developed by the Canadian Task Force.

**Discussion/Conclusions**: *CARING in Action* exemplifies a patient-oriented knowledge mobilization approach that bridges research, clinical practice, and lived experience. By embedding co-created narratives and theory-informed pedagogy, this initiative lays the groundwork for transforming pain communication education and promoting equity, empathy, and patient partnership in chronic pain care across Canada.

This work is supported by CIHR-IMHA and CIHR-SPOR-CPN.

**Investigation of Transcriptomic Changes in the Anterior Cingulate Cortex in a Mouse Model of Chronic Neuropathic Pain**

Ana Leticia Simal^1^, Xinrun Liu^1^, Ian Tobias^1^, Giannina Descalzi^1^

^1^University of Guelph

**Introduction**: Chronic pain impacts 25% of Canadians aged 15 and above, disproportionately impacting women, who constitute 67% of individuals with chronic pain. Despite this, preclinical research has historically used male rodents. Neuropathic pain, one of the most devastating subtypes of chronic pain, afflicts up to one-third of those experiencing persistent pain. The anterior cingulate cortex (ACC) is believed to be a hub for pain-induced long-term changes, with studies showing neuroplastic changes in the ACC rapidly after injury. Understanding the mechanisms involved in pain in the ACC and the biological processes driving the transition from acute to chronic pain are essential for improving diagnosis and treatment. This study investigated differential gene expression (DGE) in the ACC of female and male mice using the spared nerve injury (SNI) model of neuropathic pain.

**Methods**: Adult (8 weeks old) C57BL/6 female and male mice underwent SNI and sham surgery. Mechanical allodynia was assessed using the Von Frey test before injury, and prior to ACC sample collection at 5, 14, 30, and 60 days post-injury. Bulk RNAseq was employed, and DGE analysis of SNI over sham was performed using DESeq2.

**Results**: Despite comparable levels of mechanical allodynia in response to SNI, RNAseq analysis (p < 0.05, log_2_FC > 0.5) revealed distinct sex-dependent transcriptional patterns over time.

**Discussion/Conclusions**: These findings indicates that neuropathic pain induces sexually dimorphic transcriptional changes in the ACC, highlighting the importance of including both females and males in chronic pain research to better understand the molecular mechanisms underlying pain chronification.

## Concurrent Session One

**Session Title: Exploring targets for neuromodulation of chronic pain: Insights from neural oscillations, electrophysiology, and behavioural measures of pain sensitization and modulation**

**Session Chair**: Karen Davis

University of Toronto

**Session Abstract:**

The treatment of chronic pain remains challenging but technological advances in neuromodulation for chronic pain offers an opportunity to develop tailored solutions for personalized pain management based on individual patient characteristics. The question at hand is to identify where in the periphery or brain to target therapy and how to deliver therapy to modulate activity in a way that blocks or normalizes aberrant nociceptive activity or boosts antinociceptive activity. This symposium will examine these issues from the perspectives of a neurosurgeon (Mojgan Hodaie), and two neuroscientists (Serge Marchand, Karen Davis). The talks will explore factors that are being examined to gain insight into the mechanisms of what has gone awry in the peripheral and central nervous system in chronic pain that can be used to guide therapeutic decisions using neuromodulation. The speakers will provide data from their work that assess the patient experience of pain and the health of the nociceptive and antinociceptive systems using behavioural readouts of sensitization (temporal summation) and descending modulation (conditioned pain modulation), MRI imaging readouts of structure, and functional readouts of brain activity and oscillations (fMRI, MEG).

**At the end of this session, participants will be able to**:

- Identify novel approaches and interventional strategies that enable individualized, mechanism-informed care pathways to improve clinical outcomes of surgical treatment for neuropathic pain.
- Identify behavioural approaches used to assess pain sensitivity and modulation and evaluate their utility to help guide chronic pain management decisions.
- Identify aspects of neural activity related to pain sensitivity and modulation and evaluate their utility to help guide chronic pain management decisions.

**Speaker One**

**Title: New modalities for investigating neuromodulation targets to address chronic pain**

**Mojgan Hodaie**

University of Toronto

**Abstract**

Introduction: Treatment of chronic neuropathic pain remains challenging despite decades of progress in neurosurgery. Key obstacles include limited understanding of primary determinants of patient selection, the inherent subjectivity of pain reporting, and constraints imposed by traditional neuroanatomical targets for intervention. More recent strategies have moved beyond standard targets such as the sensory thalamus and periaqueductal gray, toward circuits and nodes implicated by systems-level models of pain.

Methods: Patients with chronic facial pain were assessed for surgical intervention and underwent: 1) validated questionnaires quantifying pain intensity and burden, 2) structural MR imaging, and 3) peripheral or central neuromodulation procedures. Both central and peripheral approaches were implemented in a staged manner to evaluate stimulation effectiveness over time and, for central neuromodulation, to identify and refine the most optimal target locations with postoperative testing and contact/site analysis.

Results: Patient assessments indicate that neuromodulation techniques are effective in improving pain metrics. Central and peripheral neuromodulation address partially distinct candidate populations. Central vs peripheral neuromodulation have a different subset of eligible patients, with central neuromodulation typically being chosen after possible failure of peripheral (and therefore less invasive) procedures.

Discussion/Conclusions: Network-level approaches are developing as a crucial component of surgical intervention for chronic neuropathic pain. This presentation will describe these novel approaches and interventional strategies that enable individualized, mechanism-informed care pathways and, consequently, better clinical outcomes.

**Speaker Two**

**Title: Understanding Variability in Endogenous Pain Modulation: Mechanisms, Clinical Potential, and Limitations**

**Serge Marchand**

Université de Sherbrooke

**Abstract**

Introduction: During this talk I will present some of our research aiming at understanding the mechanisms of endogenous pain modulation with a focus on Conditioned Pain Modulation (CPM) and Temporal Summation of Pain (TSP). Our goal is to evaluate their physiological underpinnings, variability, and potential clinical applications.

Methods: Using psychophysical, electrophysiological, and blood biomarker approaches, we assessed excitatory (TSP) and inhibitory (CPM) mechanisms in more than 300 healthy participants and 100 patients with various chronic pain conditions.

Results: Both CPM and TSP are influenced by multiple factors, including sex, gender, expectations, autonomic function, and monoaminergic biomarkers. The high intra- and inter-individual variability in these measures limits their immediate clinical applicability but provides crucial insights into the mechanisms driving pain modulation and potential targets for personalized interventions.

Discussion/Conclusions: Chronic pain treatment often relies on trial and error. Identifying reliable physiological markers could transform this process. Before CPM and TSP can serve as clinical decision tools, normative data and an understanding of their variability in healthy individuals are essential to identify true deficits in patients.

This talk will discuss our findings on the variability of endogenous pain modulation in both healthy controls and patients and examine their implications for refining clinical approaches and identifying mechanism-based treatment targets

**Speaker Three**

**Title: Informing neuromodulation targets to treat chronic pain based on brain-behaviour-pain associations in individuals**

**Karen Davis**

University of Toronto

**Abstract**

Introduction: Advancements in neuromodulation technologies have produced mixed results in alleviating chronic pain. The question of where and how to deliver neuromodulation is key to the success of a personalized approach to neuromodulation. Towards developing such a tailored approach, our lab has conducted studies to evaluate individual variability in measures of pain sensitivity and modulation that reflect the ascending nociceptive and descending antinociceptive brain pathways, and links to neural oscillations associated with chronic pain at key regions of the dynamic pain connectome.

Methods: People with chronic pain and age/sex matched healthy controls underwent 1) a standard behavioural assessment to evaluate state and trait pain, and a QST battery including conditioned pain modulation (CPM) and temporal summation (TSP), and 2) resting state fMRI and MEG to evaluate functional connectivity and neural oscillations at nodes of the dynamic pain connectome.

Results: Our studies revealed individual variability, including sex differences, in healthy individuals and people with chronic pain. We have also found links between pain and its modulation with functional connectivity of the descending antinociceptive pathways, and alpha and theta oscillations in the dynamic pain connectome. Some data also point to potential predictors of treatment outcome for neuropathic pain.

Discussion/Conclusions: I will discuss how behavioural, imaging, and MEG can identify brain sites/systems that are functioning normally or have gone awry in people with chronic pain. I will then discuss how this information can inform who, what and where to target for an individualized approach to neuromodulation for chronic pain. 

**Session Title: Innovations in addressing pain and substance use challenges in the trades**

**Session Chair: Melanie McDonald**

PainBC

**Session Abstract**

Chronic pain, mental health and substance use challenges disproportionately impact people working in the trades. Research shows that 51% of workers in the construction industry report experiencing chronic pain, putting them at increased risk for substance use, mental health issues and overdose (Umber, 2018). People living with chronic pain are four times more likely to experience depression and anxiety (Racine, 2017) and are two to three times more likely to engage in suicide-related behaviours than the general population (Cambell, 2020). Chronic pain is an overrepresented issue in the trades due to numerous factors. Historically there has been little focus on prevention of pain, a lack of research on this population and paucity of support programs tailored to people working in the trades. Pain BC’s Trades and Pain project is focused on developing tailored education and support programs to help prevent and manage chronic pain for people working in the trades. Alcohol and other drugs (substances) are often used by workers in the trades and other safety-sensitive jobs. The Canadian Centre on Substance Use and Addiction will present its findings from national studies on these and other less understood work-related risks that contribute to worker substance use. CCSA will also discuss its education and training programs to help workplaces. The purpose of this symposium is to explore pain and substance use health in the trades by sharing knowledge, opportunities, and challenges through two organizational initiatives as well as lived experience perspective. Presenters will discuss successes, challenges and outcomes to date.

**At the end of this session, participants will be able to:**

Recognize the multiple factors that drive the challenges of pain and substance use in the trades.

- Gather an understanding of 2 innovations in addressing the challenges of pain and substance use in the trades a) Trades and Pain project at Pain BC and b) Canadian Centre on Substance Use and Addiction training workshops for employers.
- Recognize the experiences of a person with lived experience with pain who worked in the trades and how this shaped him to help and engage others in the trades.

**Speaker One**

**Title: Successes, challenges and opportunities of the trades and pain project**

**Melanie McDonald**

PainBC

**Abstract**

People who work in the trades are the backbone of the Canadian economy and our communities, yet many people feel alone with their experience with pain. Research demonstrates that people working in the trades are disproportionately impacted by pain, mental health, and substance use; this presentation will highlight the numerous factors playing a role in this. According to the Action Plan for Chronic Pain in Canada, people who use substances often point to the lack of appropriate pain care as a contributor to their pain and an impediment to their treatment and recovery. In many areas across Canada there has been a lack of integration of substance use and pain care services to adequately address the unique challenges of people working in the trades.

Pain BC is a not-for-profit organization that was approached by the government, people in the trades, and trades associations to partner on solutions for preventing and managing chronic pain in British Columbia. In 2024 Pain BC launched the trades in pain project which aims to partner with workers, industry, employers and unions to develop pain-related resources and programs tailored for people who work in the trades such as peer support, a text-based support line, and pain education. The goals of this presentation will be too:

1) Highlight the factors connected to the high rates of pain in the trades

2) Discuss the success, challenges and outcomes to date of the trades and pain project and opportunities for the future

**Speaker Two**

**Title: A lived experience perspective of pain in the trades**

**James Boseley**

Saskatchewan Health Authority

**Abstract**

James grew up working in the oilfields in southern Alberta. If you talked about injuries, you were met with eye rolls and being told to “toughen up”. There was never time to be sick, injured, or anything other than putting your work boots on and getting after it! He will discuss his lived experience of working in these high-risk jobs and the mindset that went with it. Due to a workplace accident, James went down a completely different path and became a coach for Pain BC, then became a counsellor, and is now the Trades and Pain Lead. He will share this path and why he says that his story is not unique when it comes to others who work in the trades.

James will discuss the advisory committee that he has put together and the goals of the committee. He will talk about what he experienced reaching out to businesses, unions, and construction associations. James will address the plans for the project and the hopes he has for such an important and underrecognized industry.

**Speaker Three**

**Title: Canadian Centre on Substance Use and Addiction present resources and training programs for the trades**

**Shawna Meister**

Centre for Advancing Collaborative Healthcare & Education (CACHE), University of Toronto,

**Abstract**

Introduction/Aim: Maintaining safe and healthy workplaces are an increasing concern and goal among many professionals in construction and the trades, including managing work-related alcohol and other drug use (substance use). People working in these industries are at higher risk for substance use and related absences, injuries, and, in some cases, opioid-toxicity deaths (Aram et al., 2020; Fraser Health, 2018).

To better understand work-related risks and protective factors for substance use, the Canadian Centre on Substance Use and Addiction (CCSA) will share (1) findings on substance use and workplaces in various industries and, (2) resources and training programs to help workplaces better manage substance use challenges.

Methods: CCSA conducted national studies (surveys, focus groups, interviews) of workers and managers (about 1600 people) from various industries in construction, forestry, long-haul trucking, mining, oil and gas, and non-safety-sensitive industries. Data were analyzed by industry, worker and manager roles, and by sex and gender.

Results: Approximately 33% of construction workers reported consuming substances before or during work, or being intoxicated, hung over, or high. Over half (55%) reported hiding their use while working, often for fear of losing their job or what others would think of them. Reasons for use included feeling pressure to use, to be “tough”, and to manage pain.

Discussion/conclusions: Risks and protective factors to using substances are unique across industries and positions within those industries, meaning workplaces

**Session Title: Harnessing the Power of Digital Health Interventions to put Pain Management into the hands of Canadians**

**Session Chair: Max Slepian**

University of Toronto

**Session Abstract**

Approximately 1 in 5 Canadian adults live with chronic pain. Traditional treatments for chronic pain are effective, but the benefits of these treatments are limited by barriers in access. Moreover, the strongest evidence-based treatments, such as psychological interventions, are labour and time intensive. Digital health interventions (DHI) provide a means to bring these interventions to a public health scale and make them available across Canada. Such DHI can take a wide range of forms and formats. A person with lived experience of chronic pain will describe her own use of DHI to manage her pain and how it has helped vis a vis standard pain management. Dr. Patricia Poulin will describe the Power Over Pain portal, an online hub for DHI that has provided access to treatment to individuals with chronic pain across Canada. Ms. Kristina Axenova will present the development and feasibility testing of a digital translation of an evidence-based Acceptance and Commitment Therapy intervention for individuals with postsurgical pain. Dr. Max Slepian will discuss the role of autonomous conversational agents in the treatment of chronic pain and describe the development of Solace, an expert trained generative artificial intelligence conversational agent for the treatment of chronic pain. In order to make inroads against the burden of chronic pain, it will be critical to make access to DHI a routine component of pain management across Canada.

**At the end of this session, participants will be able to**:

- Articulate the stepped care 2.0 continuum of resources, principles and digital application through Power Over Pain.
- Explore learn how interest holder-informed design and iterative development can be applied to create and evaluate a scalable, digital Acceptance and Commitment Therapy (ACT) program for preventing chronic postsurgical pain and persistent opioid use.

Describe how autonomous conversational agents work and critically evaluate their role in pain management.

**Speaker One**

**Title: Power Over Pain: Building Connection and Supporting Chronic Pain Self-Management and Collective Healing**

**Patricia Poulin**

The Ottawa Hospital

**Abstract**

Developed during the COVID-19 Pandemic to respond to the needs to people living with pain in Canada, Power Over Pain (POP) is a bilingual (French/English) online portal for youth and adults living with chronic pain. It offers free access to a range of pain management resources on the stepped-care continuum, including education in various formats, self-directed courses, peer support, and interactive workshops. Patients can access the vast majority of resources without an account. Patients who wish to monitor their process can create an account and complete self-assessment at different time intervals and receive feed-back. The portal has been and continues to be co-designed with patient partners. This presentation will focus on describing the development of the portal including the role of different committees (e.g., scientific committee, lived experience advisory committee, intervention partners, IT/IS operations committee; Power Over Pain Champion Collective), engagement data, local implementation, as well as how the portal is now moving towards partnered-oriented co-development, implementation and evaluation. We will also describe how Power Over Pain is working towards becoming a vehicle to advance reconciliation in line with the Truth and Reconciliation Committee of Canada - Health-Related Calls to Action. Power Over Pain has now reached over 285,000 with more than 65,000 repeat visitors.

**Speaker Two**

**Title: Advancing Online Psychology Tools for Transitional Pain Services (ADOPT-TPS): The Development and Feasibility RCT of a Digital Acceptance and Commitment Therapy Program for Post-Surgical Pain and Opioid Use**

**Kristina Axenova**

York University

**Abstract**

The Transitional Pain Service (TPS) at Toronto General Hospital is a multidisciplinary program focused on preventing chronic postsurgical pain (CPSP) and reducing persistent opioid use. While the program demonstrates strong outcomes, its broader implementation is limited by the scarcity of specialized pain psychologists. In response to the need for scalable, accessible psychological support, we developed ADOPT-TPS (Advancing Digital Online Psychology Tools for the Transitional Pain Service), an app-based Acceptance and Commitment Therapy (ACT) program translating a psychologist-led psychotherapy group into a self-guided digital format. Our team of clinical psychologists collaborated with app developers, patients, and individuals with lived experience of pain provided usability feedback. The program was refined through iterative design sprints, which incorporated usability feedback from think-aloud and retrospective interviews. Qualitative feedback directly informed revisions to content, navigation, and accessibility features. To assess feasibility, we conducted a randomized controlled trial (RCT) comparing the digital ACT program with an online psychologist-led group. Usability testing supported the digital ACT program as feasible and acceptable. Preliminary RCT findings further demonstrated high patient satisfaction, with participants highlighting he need for pain psychoeducation in the context of post-surgical recovery, and endorsing the program’s coping strategies for chronic pain and opioid tapering. We created ADOPT-TPS to extend the reach of evidence-based pain psychology by leveraging digital delivery. Preliminary results demonstrate strong feasibility and acceptability, supporting its potential to scale psychosocial interventions for CPSP. Broader dissemination of programs like ADOPT-TPS may help close gaps in access to specialized pain care.

**Speaker Three**

**Title: Autonomous conversational agents for pain management: A new frontier in digital health interventions**

**Max Slepian**

University of Toronto

**Abstract**

Digital psychological interventions for chronic pain exist in a wide range of forms, ranging from distance-based replications of services via telephone or video conferencing to web-based treatment programs and mobile applications with content delivered in either a fully self-guided format or supported by a provider or “coach”, either synchronously or asynchronously. Recently, researchers have begun to examine provision of therapy by autonomous conversational agents, or chatbots, for management of chronic pain. Indeed, early studies have demonstrated beneficial effects of such chatbots on pain intensity and interference. Conversational agents using Large Language Model (LLM)-based generative Artificial Intelligence (AI) have upsides over rules-based models. In collaboration with MangingLife, Inc., we developed Solace, an LLM-based, expert trained generative AI conversational agent for psychological treatment of chronic pain. Solace consists of a multi-agent architecture using a library of LLMs. Each agent is responsible for a distinct conversational task, and, together, these agents are coordinated by a centralized orchestration layer that manages conversational flow, memory retention, response timing, and system-level safety controls. Safety guardrails were developed around suicidality, self- and other-harm, active emergencies and medication recommendations. In a sample of 192 individuals with chronic pain, Solace demonstrated excellent usability and was rated as an acceptable treatment option. Moreover, participants reported a strong therapeutic alliance with Solace, and demonstrated improvement in pain resilience, kinesiophobia, anxiety, and pain interference after a single 30-minute conversation. Future applications of Solace, as well as broader considerations for the use of generative AI for treatment of chronic pain, will be discussed.

**Session Title: Public Policy for Pain Amid Changing Political Winds: A Global Advocacy Symposium**

**Session Chair: Kate Nicholson**

National Pain Advocacy Center, IASP Global Advocacy Working Group

**Session Abstract**

Past Canadian Pain Society meetings have opened with the rallying cry - “This isn’t a meeting; it’s a movement” - explicitly endorsing the larger mobilization, education, and public policy agenda for pain. Advocacy for pain is a global imperative. Whether pain is integrated into global health frameworks at the World Health Organization (WHO), for example, impacts how pain is counted, prioritized, and researched. Pain policy isn’t always prescribed by borders: in North America, policy directives in the United States regarding opioid prescribing have historically shaped policies and practices in Canada. While Canada has witnessed enormous progress on pain, and the U.S. has garnered advancements, policymaking is an ever-evolving cycle. How do we maintain progress and build momentum when political priorities change, with implications ranging from available funding in Canada to outright hostility to science and global public health in the U.S.? This session brings together four leaders for a conversation on global policy for pain. Kate Nicholson, as Chair, will frame the conversation and describe current IASP work with the WHO. Maria Hudspith will survey the current landscape of national and provincial efforts in Canada. CPS President, Hance Clarke, and USASP President, Burel Goodin, will outline their visions for moving the needle on pain education and public policy. The panel will be interactive with the Chair engaging panelists in discussion of their collaborations on specific policy agendas as well as strategies for maintaining progress, resisting backward movement, and effectively targeting advocacy resources in times of political change. Panelists will take questions from the audience.

**At the end of this session, participants will be able to:**

Identify recent key pain policy advancements and changes in Canada, the U.S., and globally.

- Recognize strategies for advancing pain research and clinical practice in the policy realm nationally and globally.
- Synthesize a vision for future action, including identifying concrete ways they can advance pain policy within the contexts of their own work and jurisdictions.

**Speaker One**

**Title: Mobilizing and Sustaining Patient Advocacy in Canada**

**Maria Hudspith**

PainBC

**Abstract**

This presentation will focus on the evolution of pain advocacy in Canada, it’s diverse strategies and players, and outcomes to date. Maria will discuss Pain Canada’s role as a mobilizer, convener and backbone for the national action network driving implementation of the Canadian Pain Task Force’s Action Plan’s recommendations. Since launching in 2022, Pain Canada has expanded to include ten provincial and territorial partners, deliver support and education programs across the country, host the only annual conference by-and-for people living with pain, and lead the expansion of National Pain Awareness Week to seven languages and 500 participating organizations. Maria will share the status of the Action Plan’s recommendations, identify gaps and emerging priorities for pain advocacy in Canada.

Methods: The panelist will provide a historical overview of efforts to transform pain advocacy in Canada, through creation of the Action Plan for Pain in Canada, to development of the Pain Canada action network as an enabling structure for the Action Plan’s implementation.

Results: The panelist will address results from successfully engaging policymakers, to assembling a blueprint, to creating a national action network with a diverse set of partners.

Discussions/Conclusions: The talk will conclude with a vision of where engagement and collaboration will take the agenda for pain in Canada going forward.

**Speaker Two**

**Title: Revamping Pain Policies, Pain Research Infrastructures and De-siloing Care: The Canadian Journey**

**Hance Clarke**

University of Toronto

**Abstract**

Dr. Clarke will document the contributions that the Canadian Pain Society has made to advancing the Pain Landscape. Organizational decisions continue to move pain into the spotlight and bring a social media presence and a global audience to Canadian Pain Society events. Strategic partnerships which have been formed will aim to facilitate changes in the ecosystem with respect to revamping the way pain research is funded to improve 3.2% funding rate to pain medicine in Canada. Furthermore, Dr. Clarke will discuss the de-siloing of Pain Care as it relates to chronic pain, mental health and substance use for Canadians. To conclude, this presentation will highlight specific initiatives that the Canadian Pain Society will continue with in the years ahead to ensure a cohesive and integrated pain advocacy plan for the over 8 million Canadians living with Chronic Pain.

Methods: This talk will catalog efforts by CPS to advance the policy and educational agenda from social media to collaboration for pain in Canada.

Results: This panelist will describe the results of efforts including increased collaboration, funding, and de-siloing of related or overlapping conditions.

Discussions/Conclusions: The panelist will finish with a vision for the future to transform care for 8 million Canadians living with pain.

**Speaker Three**

**Title: U.S.-Based Education and Advocacy Amid Unprecedented Attacks on Science and Global Health Policy**

**Burel Goodin**

Washington University, St. Louis

**Abstract**

Dr. Goodin will begin by outlining the USASP’s vision with regard to education, mobilization, and policy advocacy for pain. He will describe recent efforts of USASP, engaged in collaboratively with Nicholson, to push back on the Trump Administration’s threat to cut biomedical funding by 40%, its notable omission of an earmark for pain funding, and its efforts to defund offices dedicated to diversity at the National Institutes of Health, such as the Office of Minority Health.

Thus far, these efforts have proven fruitful with reports from both the U.S. Senate and the U.S. House of Representatives holding steady on biomedical research funding with specific allocations for pain and continued funding of the Office for Minority Health, among others. Dr. Goodin will discuss additional advocacy efforts to address recent attempts to encumber patient access to treatments for pain, and educational efforts, including the use of op-eds, to push back against the spread of scientific misinformation.

Methods: This talk will present case studies of defensive advocacy in the U.S. related to scientific pain research funding, limits on access to care for people living with pain, and scientific misinformation.

Results: The panelist will describe where advocacy has succeeded, where it has fallen short.

Discussions/Conclusions: The conclusion will highlight the critical need for collaboration and mobilization (of PWLE and advocacy and scientific organizations) and present a vision of future directions.

**Session Title: Hypnosis and pain: from basic research to multimodal integration to improve chronic pain management**

**Session Chair: David Ogez**

Université de Montréal

**Session Abstract**

This symposium bridges basic and clinical research to explore how hypnosis can modulate pain through neurophysiological mechanisms to improve clinical management. The first presentation will focus on experimental studies using psychophysiological, EEG and fMRI measures, to study how suggestions for analgesia are processed by the brain to activate endogenous pain regulatory systems. The second presentation will address the integration of hypnosis and virtual reality (VRH) as a novel non-pharmacological approach for chronic pain. Results from user-experience testing demonstrated high levels of satisfaction, immersion, and perceived relief. Findings from a clinical trial confirmed the feasibility and preliminary efficacy of VRH in reducing pain intensity, anxiety. The third presentation will focus on the MUZHY project, a qualitative study exploring the experiences of patients living with chronic pain who participated in a combined hypnosis and music intervention. Thematic analysis revealed converging perspectives on the complementary effects of hypnosis and music in enhancing relaxation, emotional regulation, and reduction of pain. Video-recorded patient testimonials will illustrate lived experiences of these interventions, offering an authentic perspective on their impact in daily life. Through the two clinical research presentations, we will showcase how hypnosis, whether combined with immersive technologies or music, can foster relief, engagement, and meaning in the management of chronic pain. Together, these studies illustrate a continuum of translational research, from laboratory investigations to patient-centered innovation, highlighting the potential of hypnosis, alone or in synergy with other modalities, to improve chronic pain management.

**At the end of this session, participants will be able to**:

- Identify how hypnotic suggestions modulate neurophysiological and psychological processes involved in chronic pain.
- Explore how the combination of hypnosis and virtual reality (VRH) can enhance pain relief and patient engagement in chronic arthritic pain.
- Describe how integrating hypnosis and music (MUZHY project) can support relaxation, emotional regulation, and meaning making in people living with chronic pain.

**Speaker One**

**Title: Brain processing of analgesic suggestions during hypnosis**

**Pierre Rainville**

Centre de recherche institut universitaire de gériatrie de Montréal

**Abstract**

Experimental research demonstrates that suggestions of analgesia administered during hypnosis are effective to reduce acute pain perception. These effects are typically accompanied by a reduction of pain-evoked physiological responses, including motor and autonomic reflexes. Consistent with these effects, functional imaging studies generally show changes in brain responses evoked by acute pain stimuli during the hypnotic modulation of pain. However, the brain processing of verbal suggestions (before pain) has received little attention. Our previous work examined brain responses to metaphorical verbal suggestions to modulate pain compared to control suggestions “to feel pain normally”. Differential responses were found in the left parahippocampal region, bilateral anterior midcingulate cortex (aMCC), and right parietal operculum. These responses to verbal suggestions predicted changes in the brain response to acute painful electrical stimulation administered after the suggestions. Secondary analyses of this fMRI data set using intersubject correlation (ISC) analysis and brain-behavior representational similarity analysis (RSA) confirmed the involvement of higher-order language processing regions and revealed additional brain-behavior convergence reflecting individual differences in hypnotic susceptibility and pain modulation. This basic research provides a roadmap to develop a comprehensive neurobiological understanding of hypnosis and individual responsiveness to hypnotic suggestions, from the brain processing of verbal suggestions to the actualisation of clinical benefits.

**Speaker Two**

**Title: Virtual Reality Hypnosis: Development and clinical evaluation for chronic pain management**

**David Ogez**

Université de Montréal

**Abstract**

Chronic pain remains a major health and societal burden, with limitations in pharmacological approaches and persistent barriers to access evidence-based psychosocial care. Hypnosis and virtual reality (VR) have each demonstrated strong potential to modulate pain perception and emotional distress through complementary mechanisms: focused attention, altered body representation, and immersive distraction. Integrating these modalities within Virtual Reality Hypnosis (VRH) offers a novel, multimodal, and non-pharmacological strategy to enhance analgesia and patient engagement. This presentation will describe the development and evaluation of the VRH platform designed by our interdisciplinary team of clinicians, neuroscientists, and digital artists. Preliminary user-experience testing conducted in adults with chronic pain revealed high satisfaction, strong sense of presence, and significant perceived relief during the immersive sessions. Findings from a subsequent clinical feasibility trial confirmed the acceptability and preliminary efficacy of VRH in reducing pain intensity and anxiety, while improving relaxation and emotional well-being. Together, these findings support the feasibility of VRH as a safe, accessible, and engaging intervention to complement standard pain management. Future work aims to integrate adaptive neurofeedback and test VRH in clinical populations, paving the way toward personalized, technology-enhanced approaches for pain modulation

**Speaker Three**

**Title: A musical intervention to improve pain, anxiety, and well-being in patients with chronic pain**

**Valérie Bouchard**

Université Laval

**Abstract**

For several years, the literature has increasingly demonstrated the positive effects of music on pain and anxiety. Music is now considered one of the non-pharmacological options used in the management of chronic pain to improve patients’ daily lives. The primary objective of this study was to evaluate the feasibility, acceptability, effectiveness, and experiential dimensions associated with a music-based intervention, with and without induction, among patients attending the pain clinic at Chu de Québec.

Thirty-six participants were recruited and randomized into two groups: intervention and control. The interventions were conducted once a week, either in person or at home, over four weeks. In the intervention group, music sessions alternated between an audio montage consisting of an induction phase followed by music and music-only sessions. Semi-structured interviews were conducted at the end of the intervention period and analyzed by two independent reviewers.

Most participants reported a reduction in anxiety and an improvement in overall well-being, with some noting a decrease in pain levels following the sessions. Thematic analysis of the interviews revealed that self-connection, introspection, and personalized music selection were key elements contributing to enhanced feelings of well-being and relaxation. Participants also suggested potential improvements to help sustain and amplify the beneficial effects of the intervention.

This study paves the way for the development of a personalized music program aimed at alleviating chronic pain in affected individuals.

**Session Title: Exploring Neuromodulation for the Management of Chronic Pain: From Mechanisms to Challenges for Clinical Use**

**Session Chair: Guillaume Léonard**

Université de Sherbrooke

**Session Abstract**

Neuromodulation techniques are evolving rapidly. There is emerging evidence supporting their benefits when paired with active rehabilitation (e.g. exercises) for the management of chronic pain. By targeting peripheral and central mechanisms, transcutaneous electrical nerve stimulation (TENS), transcranial direct current stimulation (tDCS) and repetitive transcranial magnetic stimulation (rTMS) may enhance the active rehabilitation effects. For example, both peripheral and central neuromodulation can activate the opioid system and induced hypoalgesia. This hypoalgesia period may be used to facilitate the performance of exercises or of daily activities. Integrating these approaches within a multimodal intervention could strengthen pain and functional outcomes, improving adherence, and supporting longer-term improvements. This symposium aims to examine recent evidence on the complementary roles of non-invasive peripheral and brain stimulation in contemporary pain management. TENS, (objective 1), and tDCS and rTMS (objective 2) effects used either alone or in combination with active interventions for managing chronic pain will be presented. Also, current methodological limitations of the current body of evidence and the mechanisms suggested will be discussed. The objective 3 of this symposium will be to discuss the scientific evidence supporting the use of neuromodulation for different pain presentations (e.g. neuropathic pain). Also, we will discuss regulatory perspectives and challenges to clinical implementation for using these modalities in Canada. The symposium will also outline practical considerations for clinical integration such as feasibility, acceptability, and accessibility. Overall, this symposium will present state-of-the-art evidence and recommendations on neuromodulation techniques for the management of chronic pain.

**At the end of this session, participants will be able to**:

- Review recent studies and document the feasibility, acceptability, and preliminary effectiveness of applying transcutaneous electrical nerve stimulation (TENS) concurrently with exercise for the management of work-related musculoskeletal pain.
- Discuss recent evidence on the effects of rTMS alone or in combination with exercise for the management of chronic low back pain and musculoskeletal pain and discuss the current limitations in the current body of evidence in this field.
- Review current evidence testing the effects of non-invasive neuromodulation modalities (e.g., rTMS and tDCS) for chronic pain and to discuss the challenges and key considerations related to their implementation in Canadian rehabilitation and pain management settings.

**Speaker One**

**Title: Combining TENS and exercise for musculoskeletal pain: feasibility, acceptability, and preliminary outcomes from a pilot randomized trial.**

**Adrien Nourry**

Université de Sherbrooke

**Abstract**

Introduction: Exercise plays a central role in managing musculoskeletal (MSK) pain. However, pain during movement remains a major barrier to adherence. When applied concurrently with exercise, transcutaneous electrical nerve stimulation (TENS) can temporarily relieve movement-related pain, potentially facilitating exercise performance and promoting adherence. This study aims to (i) document the feasibility and acceptability of combining TENS with exercise among workers with MSK pain and (ii) explore its preliminary effectiveness on pain-related outcomes.

Methods: This quadruple-blind pilot randomized controlled trial includes 24 adults with work-related MSK pain. Participants are randomly assigned to receive active or sham TENS applied concurrently with exercise over a 3-week period (seven 30-minute sessions per week). Feasibility (e.g., recruitment rate, session adherence/retention, safety) and acceptability (e.g., ease of use, perceived usefulness, satisfaction) are assessed, and preliminary effects on pain, kinesiophobia, movement, and other pain-related outcomes are documented using validated measures.

Results:  Eighteen participants have completed the study. Preliminary findings indicate that the intervention is safe, well tolerated, and acceptable, and perceived as useful, with no adverse events reported. Recruitment has been slower than expected (since May 2024); non-completion 2/18 and missed session in 2/18 participants. Completion of data collection is scheduled for January 2026, with final results and preliminary effects to be presented at the conference.

Discussion/Conclusion: These findings will provide preliminary evidence on the potential of combining TENS with exercise for workers with musculoskeletal pain. If feasible and acceptable, this approach could serve as a complementary strategy to reduce movement-related pain and improve work rehabilitation outcomes

**Speaker Two**

**Title: Repetitive transcranial magnetic stimulation combined with motor control exercises for the management of chronic low back pain.**

**Hugo Massé-Alarie**

University Laval

**Abstract**

Introduction: Exercise are recommended for chronic low back pain (CLBP) management but with modest effects. Repetitive transcranial stimulation (rTMS) is a non-invasive technique that can target specific brain areas, with the potential to relieve pain and enhance exercise effects. However, its efficacy in CLBP remains uncertain. Considering CLBP is heterogenous, exercises and rTMS can be more effective in subgroups of patients. Our aims were to determine (i) effects of rTMS combined with exercises in CLBP and (ii) if subgroups of patients can best respond to these interventions.

Methods: 140 participants with CLBP were randomized into 4 groups (rTMS; sham rTMS; rTMS+exercise; sham rTMS+exercise). Participants received 10 intervention sessions over 8 weeks. For the primary objective, the primary outcome was pain intensity. For the secondary objective, outcomes were pain intensity and disability, and potential moderators were: pain self-efficacy, kinesiophobia, “central sensitization” symptoms, etc. Linear mixed models were used for both objectives.

Results: For the primary objective, rTMS and exercises was not superior to the other groups when combined or compared to sham rTMS or no exercises. For the secondary objective, participants with high “central sensitization” symptoms who received rTMS had higher disability compared to participants with low score and receiving rTMS, and compared to participants with high score and receiving sham rTMS.

Discussion/Conclusions: In CLBP, rTMS and exercises are not superior than a sham. Also, rTMS prevented disability improvement in patients with CLBP with “central sensitization”. If confirmed, it will be imperative to strongly recommend against rTMS use in this subgroup

**Speaker Three**

**Title: Non-Invasive Neuromodulation for Chronic Pain: Evidence and Implementation Challenges in Canada**

**Luciana Macedo**

McMaster University

**Abstract**

Introduction: Over the last decades, the use of neuromodulation has increased significantly, driven by technological advancements and emerging evidence. Neuromodulation is defined as the alteration of nerve activity through targeted delivery of a stimulus, such as electrical stimulation, to specific neurological sites in the body. Common non-invasive modalities, that specifically target the brain, include Repetitive Transcranial Magnetic Stimulation (rTMS) and Transcranial Direct Current Stimulation (tDCS).

This presentation aims to review current evidence supporting prominent non-invasive neuromodulation modalities for chronic pain management, and to examine the challenges associated with their implementation in Canada within outpatient care settings.

Results: Experimental studies have shown that neuromodulation can produce local effects, such as changes in inflammatory biomarkers, as well as long-term network level effects, such as enhanced engagement of descending pain modulation. Current evidence for rTMS suggest benefits for neuropathic pain of various origins, fibromyalgia and migraine, but insufficient evidence for orofacial pain, low back pain, myofascial pain and complex regional pain syndrome. An existing systematic review on tDCS for chronic primary pain have found significant reduction in pain whether tDCS was applied alone or in combination with other non-invasive non-pharmacological therapies. Interestingly, while neuromodulation shows promising applications, most evidence suggest that regardless of the application, its effects can be augmented when delivered in conjunction with rehabilitation.

Discussion/Conclusion: Emerging evidence has supported the role of non-invasive neuromodulation for some but not all chronic pain conditions. As the body of evidence grows, implementation challenges need to be addressed both from regulatory and health systems perspective.

## Concurrent Session Two

**Session Title: The (surprisingly) short road from genome-wide studies to pain management**

**Session Chair: Burel Goodin**

Washington University School of Medicine, St. Louis

**Session Abstract**

Chronic pain is a multifactorial condition shaped by genetic, psychological, immune, and behavioral factors. Yet these domains are often studied separately, limiting translation of discoveries into integrated treatment strategies. This symposium combines genomic evidence across mental health, immune function, and alcohol use to advance a holistic understanding of chronic pain vulnerability and its clinical management. Katerina Zorina-Lichtenwalter presents findings from Genomic Structural Equation Modeling (GenomicSEM) showing that diverse pain conditions load onto a single general pain susceptibility factor. This factor is genetically correlated with poorer mental health and lower cognitive performance, highlighting shared genetic influences that support biopsychological approaches to treatment. Pamela N. Romero Villela extends this framework to the immune system, identifying moderate genetic correlations between chronic pain and inflammatory markers (rg ≈ 0.45) and smaller but significant overlap with autoimmune diseases (rg ≈ 0.28). These results emphasize the role of shared immune pathways in chronic pain susceptibility and the importance of screening for co-occurring inflammatory or autoimmune conditions. Heval Şeker examines multidimensional genetic links between alcohol use behaviors (AUBs) and pain. Using GenomicSEM and factor analyses across 40 traits, Şeker identifies three latent AUB dimensions—problematic drinking, social drinking, and controlled heavy consumption—with distinct genetic relationships to pain. Problematic drinking correlates positively with pain, whereas social and controlled heavy drinking show negative or null associations. Together, these studies reveal convergent genetic architectures linking psychological, immune, and behavioral domains in chronic pain, reinforcing the need for integrative, multidisciplinary management approaches.

**At the end of this session, participants will be able to**:

- Illustrate how large-scale genomic approaches can be translated into practical insights for understanding and treating pain.
- Analyze the specific genetic relationships between pain, immune and mental health conditions, cognitive abilities, and alcohol use behaviors.
- Evaluate the clinical implications of genetic correlations to inform and support integrated, biopsychological approaches to chronic pain management.

**Speaker One**

**Title: Insights from Genomic Structural Equation Modeling of pain and mental health about integrative pain management**

**Katerina Zorina-Lichtenwalter**

University of Colorado

**Abstract**

Introduction: Chronic pain genetics has historically been siloed, limiting its translation to clinical practice. Findings are often communicated using domain-specific terminology and visualizations that restrict accessibility beyond the field. Meanwhile, most discoveries remain at the molecular level, distant from patient-relevant applications. Recent genome-wide association studies (GWAS) now allow integrative analyses that connect chronic pain with other biological and psychological domains. This discussion aims to highlight the shared genetic architecture of chronic pain and to examine its relationships with mental health and cognitive traits as a path towards treatment options.

Methods: Analyses using Genomic Structural Equation Modeling and genome-wide summary statistics from well-powered datasets conducted to examine genetic relationships across pain traits and between pain traits and psychiatric and cognitive traits derived from large GWAS datasets.

Results: Pain conditions loaded onto a single general susceptibility factor. The general pain factor is positively correlated with mental health traits and neuroticism and negatively correlated with cognitive performance.

Discussion/Conclusions: Findings suggest that chronic pain vulnerability extends beyond tissue damage and includes substantial psychological and environmental components. The shared, non-causal overlap with mental health disorders underscores the need for studies addressing integrated treatment approaches. The inverse relationship with cognition highlights potential benefits of cognitive-based interventions. Together, these results bridge molecular discoveries and clinical relevance, advancing a biopsychological framework for understanding and managing chronic pain.

**Speaker Two**

**Title: Evaluating the genetic overlap and relationship between chronic pain, inflammation, and immune disease**

**Pamela N. Romero Villela**

Washington University, St. Louis

**Abstract**

Introduction: Chronic pain often co-occurs with immune disorders and chronic inflammation. I will explore whether shared genetic factors help explain why chronic pain often co-occurs with various immune traits, disorders, and markers of inflammation. By understanding to what degree and what parts of the genome are shared amongst these conditions, future studies can further elucidate molecular mechanisms underlying their shared genetic factors and later develop better therapeutics for both conditions.

Methods: I will use Genomic Structural Equation Modeling (genomic SEM) and genome-wide summary statistics from the largest powered chronic pain, inflammation, and immune traits to quantify the global genetic overlap between these traits and diseases. I will then use LAVA to pinpoint the genomic areas that are driving the global genetic correlation between chronic pain and immune traits. Lastly, I will use Mendelian Randomization to elucidate causality between chronic pain and the immune traits investigated.

Results: Preliminary analyses found a moderate genetic correlation of 0.45 between chronic pain and inflammation markers (e.i., C-reactive protein and IL6). Conversely, the genetic correlation between chronic pain and a subset of autoimmune diseases showed a modest genetic correlation of 0.28.

Discussion/Conclusions: These findings underscore the wide-ranging role of the immune system in chronic pain. Shared genetic factors partially help explain the high co-occurrence of chronic pain and autoimmune disease and inflammation among patients. From a clinical perspective, this study highlights the importance of checking if patients with autoimmune disease further meet criteria for chronic pain and vice versa.

**Speaker Three**

**Title: Genetic Correlations and Latent Factor Structure of Alcohol Use Behaviors in Relation to Pain Conditions: Implications for Clinical Management**

**Heval Şeker**

Dokuz Eylul University School of Medicine, Izmir, Turkey

**Abstract**

Introduction: Alcohol use behaviors (AUBs) and pain conditions are both common, moderately heritable, and frequently co-occur clinically. Shared genetic liability may contribute to this overlap, yet alcohol phenotypes are multidimensional and may differentially relate to pain. This study quantified genetic correlations between AUBs and pain traits and evaluated whether latent AUB dimensions show distinct genetic associations with pain liability.

Methods: Linkage Disequilibrium Score Regression estimated genetic correlations (rg) across 40 traits including musculoskeletal, gastrointestinal, and headache-related pain, alongside beverage type, drinking patterns, and problematic-use indicators. Exploratory factor analysis (FA) defined the latent structure of AUBs and supported a confirmatory FA integrating alcohol and pain dimensions.

Results: Problematic alcohol consumption showed positive genetic correlations with pain (e.g., arthritis with past alcohol problems: rg = 0.52), whereas higher drinking frequency showed negative correlations (e.g., chronic widespread pain: rg = −0.35). A three-factor AUB structure emerged: F1: Problematic drinking, F2: Social drinking, and F3: Controlled heavy consumption. The integrated model demonstrated good fit (χ^2^(703) = 10,785; CFI = 0.95; SRMR = 0.10). F1 was positively associated with both General Pain (rg = 0.40) and Musculoskeletal Pain (rg = 0.10), F2 showed negative correlations with General Pain (rg = -0.53) and Musculoskeletal Pain (rg = -0.17), while F3 was negatively associated with General Pain (rg = −0.19) and not significantly related to Musculoskeletal Pain.

Discussion/Conclusions: Findings indicate heterogeneity in shared genetic risk underlying alcohol-pain relationships: problematic use is associated with higher chronic pain liability, while other drinking patterns show more variable associations. These findings support nuanced alcohol-risk screening in pain care and integrated treatment approaches.

**Session Title: Psychedelics and Chronic Pain—From Mechanisms to Ethical Trial Design**

**Session Chair: Brittany Rosenbloom**

University of Toronto

**Session Abstract**

Despite decades of research, many with chronic pain achieve only modest relief and limited functional gains, which is often worse for particular chronic pain conditions, such as neuropathic pain. A core challenge is the multidimensional nature of pain (sensory, affective, and cognitive) which leads to the failure of nociception-only approaches when distress, fear, and maladaptive learning sustain the pain experience. Psychedelic-assisted psychotherapies (MDMA-, psilocybin-, and Ketamine- assisted psychotherapy) offer a potential advancement by coupling rapid neuroplastic shifts with structured psychotherapy to recalibrate appraisal, reduce avoidance, and improve function. Nevertheless, there are barriers to psychedelic-assisted psychotherapies gaining evidence, such as heterogeneous protocols, uncertain dosing/psychotherapy models, evolving safety/ethics and regulatory pathways, and limited pain-specific evidence. This symposium explores the gaps in research through a historical lens and addresses each gap by synthesizing mechanisms relevant to chronic pain, critically appraising the evidence, and providing concrete, field-tested trial templates. The faculty’s active leadership of three federally funded clinical trials ensures practical guidance on design, safety monitoring, training/fidelity, and knowledge translation (Drs, Goel and Ladha). Inclusion of a clinical psychologist (Dr. Rosenbloom) ensures depth on psychotherapy and integration, elements central to durable outcomes. This symposium translates cutting-edge evidence into practical frameworks for rigorous trials and real-world implementation.

**At the end of this session, participants will be able to**:

- Discuss evidence-based psychotherapy development within the context of clinical trials for MDMA-assisted psychotherapy for chronic pain and be able to critically appraise the ethical underpinnings of therapies designed for marginalized populations, such as those who have experienced trauma.
- Describe how our program operationalizes ketamine, psilocybin, and MDMA trials for chronic pain; interpret early quantitative and qualitative findings from the KAP pilot; and apply patient-partner insights and methodological safeguards to design rigorous, patient-centered trials.
- Identify key methodological and ethical challenges in psychedelic clinical trials for chronic pain and apply strategies to design studies that avoid common pitfalls from past research.

**Speaker One**

**Title: MDMA‑Assisted Group Psychotherapy for Comorbid Fibromyalgia and PTSD: Developing an Inclusive, Trauma‑Informed Framework for Pain and Trauma Integration**

**Brittany Rosenbloom**

University of Toronto

**Abstract**

Introduction: Chronic pain disproportionately affects marginalized people—including those who have experienced past trauma or violence and as a result are suffering with Post-traumatic Stress Disorder (PTSD). Current chronic pain treatments fail for more than 50% of patients. Recent developments suggest that psychedelic assisted therapy, such as 3,4-methylenedioxymethamphetamine (MDMA) paired with psychotherapy, could lead to a paradigm shift that includes novel therapeutics that will improve the lives of patients with concomitant pain and PTSD. MDMA-assisted psychotherapy may help reframe maladaptive thoughts about pain and alter serotonin-mediated pain pathways, leading to improved treatment outcomes.

Methods: Our research team devised an exploratory study of 16 participants with fibromyalgia who also have a history of trauma and symptoms consistent with PTSD. Participants will have a single group dosing session of 120mg of MDMA (with an optional booster of 60 mg) combined with group psychotherapy for preparation sessions prior to dosing and integration sessions after dosing. The focus of the initial stage of study development is on the development of psychotherapy and integration models that target both pain and trauma reactions.

Results: Our research team, which consists of psychologists, psychotherapists, psychiatrists, and pain physicians, devised a novel group-based psychotherapy model that involves elements of traditional integration seen with psychedelics as well as evidence-based modalities for chronic pain and trauma reactions. (i.e. Acceptance and Commitment Therapy and Internal Family Systems). The development of this novel treatment is explored through a historical lens and built upon a systematic review and meta-analysis for treating concomitant chronic pain and trauma-related symptoms.

Discussion/ Conclusions: This exploratory, ethically-driven, MDMA‑assisted group psychotherapy protocol for comorbid fibromyalgia and PTSD aims to reframe pain, enhance trauma processing, and inform larger trials; if effective, it could transform psychotherapy access and outcomes for marginalized patients with chronic pain and trauma.

**Speaker Two**

**Title: Clinical Research in Psychedelics: Avoiding Past Mistakes**

**Karim Ladha**

University of Toronto

**Abstract**

Introduction: The resurgence of interest in psychedelic-assisted therapies has generated optimism for their potential role in managing chronic pain. However, the current wave of research risks repeating methodological and ethical pitfalls seen in earlier eras of psychedelic and chronic pain research. This presentation aims to highlight core concepts, methodological challenges, and strategies to ensure rigor and credibility in clinical trials evaluating psychedelics for chronic pain.

Methods: A critical review of historical and contemporary psychedelic research will be conducted, emphasizing study design, bias control, participant selection, outcome measurement, and regulatory considerations. Lessons learned from prior studies will be synthesized to identify best practices for future trials.

Results: Analysis has revealed recurring issues, including inadequate blinding, expectancy effects, inconsistent dosing protocols, and challenges in distinguishing pharmacologic from psychotherapeutic effects. Recent advances in trial methodology such as active placebos, improved outcome metrics, and standardized integration protocols offer promising pathways to enhance validity and reproducibility.

Discussion/Conclusions: Avoiding past mistakes requires a disciplined, transparent, and multidisciplinary approach to psychedelic research. Future trials in chronic pain should prioritize methodological rigor, ethical oversight, and the disentanglement of psychological and pharmacologic mechanisms. By doing so, the field can advance credible evidence to guide safe and effective clinical applications.

**Speaker Three**

**Title: Clinical Research in Psychedelics for Chronic Pain: Program Update, Ketamine-assisted psychotherapy (KAP) Pilot Signals, and Patient Voices**

**Akash Goel**

St. Michaels Hospital

**Abstract**

Introduction: To provide a succinct update on our ketamine-, psilocybin-, and MDMA-assisted psychotherapy research program in chronic pain—how studies are run, how we recruit, early quantitative and qualitative signals from our ketamine-assisted psychotherapy (KAP) pilot, and the planned inclusion of patient partners to share lived experience during the panel.

Methods: Prospective, single-site pilot using a manualized ketamine-assisted psychotherapy protocol. Participants complete standardized preparation sessions, undergo protocolized ketamine dosing in a medically supervised therapy room, and receive structured integration until 16-weeks. A therapist delivers care with training on the KAP manual; fidelity is monitored via checklists, supervision, and periodic session review. Safety is ensured with on-site monitoring during dosing, systematic AE capture, and predefined escalation/notification pathways. Recruitment occurs through partnered pain clinics and patient groups; eligibility is confirmed via staged screening and informed consent. Outcomes are captured via PROs (pain interference/function and mood/anxiety measures) and brief, structured qualitative interviews.

Results: Quantitative (KAP pilot): Target enrolment within ~18 months; current sample N = 30. Adherence >70% in all 3-arms. Adverse events were transient (e.g., dissociation, nausea) with 0 SAEs related to study drug. At 30 days: Pain interference achieving MCID was >75% in combined arms but <40% both monotherapy arms.

Qualitative themes: Preparation reduced anxiety and clarified expectations; dosing-day environment (calm room, continuous monitoring, therapist presence) fostered safety; integration supported pacing, acceptance, and functional coping. Barriers included travel/caregiver burden and scheduling.

Discussion/Conclusions: A psychotherapy-integrated, medically supervised model for psychedelic trials in chronic pain is feasible with strong adherence and acceptable safety. Early KAP signals justify progression to randomized, active-control designs with blinded outcomes and explicit expectancy management. Our scalable infrastructure (therapist training, therapy rooms, pharmacy/IP, ePROs, monitoring) supports upcoming psilocybin and MDMA trials. Incorporating patient partners on stage centers patient priorities and strengthens recruitment and integration practices.

**Session Title: Innovations in Veteran-Centered Research: Addressing Chronic Pain and Mental Health**

**Comorbidities through Integrated Approaches**

**Session Chair: Jenny (Jing Wen) Liu**

MacDonald Franklin OSI Research Centre

**Session Abstract**

In this symposium, we present a coordinated program of research that addresses the high prevalence of chronic pain among Canadian Veterans, with a focus on its intersection with mental health and the transition to civilian life. These efforts aim to uncover the systemic, interpersonal, and individual barriers that Veterans, families, and service professionals face and co-develop evidence-based, Veteran-informed solutions that foster resilience, recovery, and well-being. Studies included in this symposium consist of a foundational mixed-methods study that examines how chronic pain amplifies transition challenges and reduces life satisfaction, with qualitative insights revealing how pain disrupts identity, relationships, and mental health amid systemic barriers and stigma. Complementary projects include one that explores pain trajectories, barriers to care, and treatment preferences across different stages of the chronic pain experience, and a qualitative study on Veteran amputees and their caregivers to identify critical service gaps, and a stepped care program evaluation in British Columbia that assesses the feasibility and impact of an interdisciplinary clinic model tailored for Veterans, capturing clinical, social, and economic outcomes. Across all projects, integrated knowledge translation, Veteran engagement, and a focus on equity guide the co-design of sustainable, trauma-informed solutions. Together, these initiatives form a multi-pronged research strategy to enhance chronic pain management, improve mental health, and support meaningful post-service reintegration. They also inform policy recommendations and clinical innovations that can be adapted across contexts to serve Veterans more effectively.

**At the end of this session, participants will be able to:**

Identify key factors that contribute to chronic pain and mental health comorbidities in Canadian Veterans.

- Explore models of care their potential to improve access, coordination, and outcomes for Veterans with chronic pain.

Apply equity-focused, trauma-informed principles when considering policy, service design, and delivery.

**Speaker One**

**Title: Bridging Systems of Care: Collaborative Pathways to Improve Chronic Pain Care for Canadian Veterans**

**Erin Collins**

MacDonald Franklin OSI Research Centre

**Abstract**

Introduction: Canadian Veterans with chronic pain face persistent challenges navigating complex systems to access and engage with essential services and supports. These barriers extend beyond healthcare, encompassing administrative, informational, and systemic factors that disrupt continuity of care. This symposium aims to: (1) present perspectives from Veterans with lived experience, family members, and service providers (healthcare professionals and case managers) on the enduring gaps that cause Veterans to “fall through the cracks”; and (2) engage attendees in co-developing strategies to strengthen system integration and Veteran-centered chronic pain care.

Methods: From January to September 2025, 31 qualitative interviews were conducted with Veterans, family members, and service providers. A mixed inductive-deductive analysis identified barriers, facilitators, and protectors of well-being related to access, engagement, and system navigation.

Results: Veterans described ongoing struggles accessing both direct healthcare and indirect supports affecting pain management, such as financial, psychosocial, and administrative resources. Family members reported a gap between awareness of available supports and their practical accessibility, often carrying heavy coordination burdens. Service providers described working in silos with limited collaboration and knowledge of military culture. Protective factors included continuity of care, early intervention, and strong peer and family support networks.

Discussion/Conclusions: Barriers to care stem less from a lack of services than from systemic fragmentation and poor coordination. Findings underscore the need for integrated, sustainable, and culturally informed models of chronic pain care. Attendees will gain practical insights and co-develop strategies to strengthen navigation, communication, and collaboration.

**Speaker Two**

**Title: Evaluating a Stepped Care Model for Canadian Veterans with Chronic Pain**

**Jenny Liu**

MacDonald Franklin OSI Research Centre

**Abstract**

Introduction/Aim: Chronic pain affects nearly 50% of Canadian Veterans, often co-occurring with PTSD, depression, and other mental health challenges. Transition periods exacerbate access barriers due to changes in coverage and limited care continuity. This study evaluates the acceptability, feasibility, and impact of a provincial, community-based stepped care model implemented at the the Changepain clinic in British Columbia for Canadian Veterans, assessing how it addresses these barriers and supports pain care in transition periods.

Methods: Using a mixed-methods, longitudinal design, the study follows 30 Veterans and their families over 12 months as they engage with the Changepain stepped care program. Data collection includes clinical evaluations, standardized surveys (at baseline, 3, 6, and 12 months), and semi-structured interviews. Indicators span clinical, familial, social, and economic outcomes.

Results: In this presentation, we will present our shared learnings from project set-up and share preliminary findings to date.

Discussion/Conclusions: This community-based stepped care model is a promising model of care that addresses long-standing gaps in Veteran pain care. Results are examined in the context of the model’s potential for scale-up, particularly in regions with high transition-related drop-offs in care. Implications will inform Veteran-centered pain policy, clinical planning, and cross-sectoral coordination.

**Speaker Three**

**Title: Embedding Lived Experience in Chronic Pain Research with Veterans and Families**

**Erin Collins, Jenny Liu & J. Don Richardson**

MacDonald Franklin OSI Research Centre

**Abstract**

Introduction: Despite growing interest in patient-oriented research, few frameworks adequately capture how to engage Veterans, families, and service professionals in research addressing chronic pain and comorbid mental health. This presentation reflects on methodological lessons from a suite of studies conducted at the MacDonald Franklin OSI Research Centre, exploring how lived experience shaped all phases, from design through knowledge mobilization.

Methods: Across multiple projects, including studies on transition experiences, treatment trajectories, amputee Veteran care, and service delivery, Veterans, spouses, and providers were engaged as participants, advisors, and co-creators. Data sources included qualitative interviews, advisory sessions, and iterative feedback loops. We applied trauma-informed, equity-based approaches with intersectional sampling strategies.

Results: Lived experience contributed to reframing research questions, centering real-world complexity, and surfacing hidden barriers (e.g., caregiver burden, stigma in system navigation). Veterans shaped the language, recruitment strategies, and interpretation of findings, leading to more nuanced and contextually grounded insights. Service professionals provided critical system-level context, while family members highlighted invisible labor and relational impacts.

Discussion/Conclusions: Embedding lived experience strengthened the relevance, rigor, and impact of all projects. However, meaningful engagement requires time, trust, compensation, and flexible methods. We examine how Veteran pain research could incorporate lived experience as a core competency to shift power dynamics, improve trust, and generate solutions that work in real life.

**Session Title: Measuring the Quintuple Aim in Healthcare Systems in the Context of Chronic Pain:**

**Advancing Patient Experience, Population Health, Costs Efficiency, Clinician Well-being, and Equity**

**Session Chair: Marimée Godbout**

Université du Québec en Abitibi-Témiscamingue

The Quintuple Aim, encompassing better experience of care, improved population health, reduced costs, clinician well-being, and equity, has emerged as a key framework for guiding healthcare quality improvement across diverse healthcare settings. While widely adopted in primary care, hospital management, and public health, its application to chronic pain management research remains limited. Chronic pain presents unique challenges, including complex patient needs, long-term treatment trajectories, and significant demands on clinicians, underscoring the urgent need for a comprehensive, system-level approach that integrates multiple perspectives. Understanding the relevance of the Quintuple Aim in this context enables clinicians, decision-makers, and researchers to identify appropriate measurement strategies and indicators for each dimension, from patient-reported outcomes to population health metrics, fostering a culture of measurement that actively supports a learning health system and continuous improvement. This symposium will explore, using concrete examples, how the Quintuple Aim has been operationalized and measured in Canadian quantitative and qualitative research. Drawing from studies in chronic pain management, presenters will illustrate how each dimension, including patient experience, population health, cost efficiency, provider well-being, and equity, can be effectively assessed and used to guide meaningful improvement efforts.

**At the end of this session, participants will be able to**:

- Recognize the relevance of the Quintuple Aim framework (better experience of care, improved population health, reduced costs, clinician well-being, and equity) clinician well-being, and equity) in the context of chronic pain healthcare quality improvement.

Distinguish between the measurement strategies and the indicators used to evaluate progress toward each aim.

- Analyze the application of the Quintuple Aim in chronic pain research using concrete examples from both quantitative and qualitative research.

**Speaker One**

**Title: Measuring the Quintuple Aim in Pain: Strengthening Patient-Centred and Equitable Pain Care**

**M. Gabrielle Pagé**

Université de Montréal

**Abstract**

Introduction: The Quintuple Aim offers a system-level framework to improve healthcare through enhanced patient experience, better population health, reduced costs, improved provider well-being, and greater equity. Yet, in pain research and clinical practice, implementing rigorous measurement strategies that meaningfully capture patient perspectives remains a major challenge.

Methods: In this presentation, Gabrielle Pagé will discuss methodological innovations for measuring patient experiences within the context of pain care.

Results: The talk will first highlight intensive longitudinal approaches—such as ecological momentary assessment and daily diary methods—to capture within-person variability in pain and its psychosocial correlates. These approaches can help tailor personalized pain management and improve the precision of intervention delivery. Next, emphasis will be placed on linking patient-reported outcome measures (PROMs) and patient-reported experience measures (PREMs) with system-level performance indicators, drawing on data integration strategies developed in recent studies. Such linkage between PROMs/PREMs, registries, and administrative data can provide actionable insights into treatment responsiveness and equity in access and outcomes. Finally, the presentation will address methodological and ethical considerations for embedding these measures within learning health systems.

Discussion/Conclusions: Strengthening measurement approaches aligned with the Quintuple Aim can accelerate progress toward integrated, equitable, and person-centred models of pain care.

**Speaker Two**

**Title: Costs at the Intersection of Population Health and Equity: Exploring Healthcare Costs of Chronic Back Pain through the Lens of the Quintuple Aim Framework**

**Jessica Wong**

Western University

**Abstract**

Introduction/Aim: The Institute for Healthcare Improvement’s Quintuple Aim calls for advancing patient experience, population health, workforce well-being, equity, and cost optimization. Back pain - a major contributor to disability and health systems burden in Canada and globally - illustrates the challenges of achieving these aims amid high costs, growing burden on populations, and health inequities. This presentation examines healthcare costs for chronic back pain in Canada through the lens of population health and equity within the Quintuple Aim framework.

Methods: This presentation will use a case study approach focusing on chronic back pain in Ontario, Canada to explore healthcare costs at the intersection of population health and equity.

Results: A review of recent research will illustrate the distribution and potential drivers of healthcare costs within this back pain population. The presentation will discuss methodological approaches used in this research, including healthcare costing methodology and analyses of linked population health survey and administrative data. Social determinants of health will be discussed to explore inequities influencing access to care and costs. This presentation will also highlight reflections on future directions towards optimizing costs, reducing inequities, and improving population health for people living with chronic back pain.

Discussion/Conclusions: Guided by the Quintuple Aim framework, this presentation will invite reflection on how healthcare costs for chronic back pain intersect with population health and equity considerations. The discussion will explore the complexities around how health systems can balance efficiency, population health, and equity in the management of back pain and similar high-burden conditions.

**Speaker Three**

**Title: What Gets Measured Gets Improved: Understanding Clinician Well-being**

**Anaïs Lacasse**

Université du Québec en Abitibi-Témiscamingue

**Abstract**

Introduction: Recently, clinician well-being has faced significant challenges, whether due to workforce shortages, the COVID-19 pandemic, or issues surrounding remuneration for healthcare providers across several Canadian provinces. Understanding and assessing clinicians’ experiences of care and well-being, including job satisfaction, fulfillment, and burnout, provides critical insights into how the practice environment shapes their work and influences workforce retention, patient safety, and overall quality of care. This is particularly important in chronic pain management, where patients often present with complex, long-term needs that place sustained demands on providers. For example, clinicians may experience compassion fatigue, a state of exhaustion and distress resulting from repeated empathic engagement with individuals in pain. By fostering a culture of measuring clinician well-being, researchers and healthcare organizations can identify strategies to enhance care delivery, support the workforce, and ultimately improve patient outcomes. Valid, reliable measurement instruments are essential to these efforts.

Methods: In this symposium, Dr. Anaïs Lacasse will present recent advancements in the assessment of clinician well-being.

Results: Specific topics will include clinician well-being conceptual frameworks, systematic review results about validated questionnaires, and gaps in the literature, such as the lack of sex- and gender-based validation studies. She will discuss how these tools can be applied in the Canadian context and introduce a new bilingual toolbox designed to support measurement across diverse Canadian healthcare settings.

Discussion/Conclusions: The session will provide examples of how assessing clinician experiences can inform strategies to optimize care, support providers, and promote sustainable, high-quality healthcare for people living with chronic pain.

**Session Title: Medico-Legal Conundrum of Chronic Pain: A Practical Guide for Clinicians**

**Session Chair: Michael Gofeld**

University of Toronto

**Session Abstract**

This practical and interactive symposium introduces healthcare professionals to the medico-legal aspects of chronic pain care, with the goal of increasing awareness of the medico-legal interface, clarifying professional roles, and alerting clinicians to common and often unintended pitfalls. Because chronic pain frequently enters medico-legal systems, driven in part by the challenge of subjective symptoms within a framework grounded in objective evidence, almost all clinicians will interact with the legal system at some point in their careers, whether through documentation for insurers, release of clinical notes, testimony as a treating provider, or, less commonly, participation as an independent medical evaluator or expert witness. The symposium begins by addressing the foundational question of why the medico-legal world exists, exploring concepts such as the distinction between proof and care, the tensions created by so-called “invisible pain,” and the important principle of conflict between objective findings and subjective complaints in medico-legal processes. The aim is to demystify the medico-legal system by explaining its function as an impartial, rules-based process that delivers unbiased decisions, rather than advocating for or against any individual. The program will then clarify professional boundaries by distinguishing the roles of treating clinicians, independent medical evaluators, experts, and witnesses of fact, and by outlining the respective functions of insurers, lawyers, and judges—emphasizing that expert status is formally conferred only by the court. Finally, the symposium will provide practical guidance on clinical documentation in medico-legal contexts, highlighting that clinical notes are often read outside their original clinical intent and that prioritizing function, avoiding causal overreach, expressing uncertainty appropriately, and steering clear of advocacy-based language are essential to reduce unintended consequences. This symposium is not intended to train clinicians to practice medico-legal work or become IME experts, but rather to help clinicians understand the medico-legal arena in which their clinical work may be scrutinized. Delivered in a panel-discussion format with brief topic introductions followed by moderated discussion and audience Q&A, the session is offered by the Medico-Legal Special Interest Group and is intended for all clinicians caring for people with chronic pain, including physicians, physiotherapists, occupational therapists, psychologists, and nurses.

**Speaker One: R. Deamo Assis** (Centre Intégré de Santé et Services Sociaux d’Abitibi-Témiscamingue)

**Panelists: O. Finlayson** (Mackenzie Health Hospital)**, R. Nemeth** (N**emeth Psychology Prof. Corp),**

**M. Fitzcharles** (McGill University)

Why Does the Medico-Legal World Exist? (Foundations): Panelists will discuss why chronic pain is a challenge for the medico-legal world, especially in the context of subjective symptoms that cannot be accurately measured for a system that relies on objective proof.

**Speaker Two: R. Nemeth**

**Panelists: O. Finlayson, R. Deamo Assis, M. Fitzcharles**

Who Is Who? (Roles and Boundaries): The roles of individuals in the medico-legal interface will be examined, including that of the treating clinician, the medical expert, lawyers, insurers and the judge. Specific attention will be paid to the input of treating healthcare professionals who may not be aware of their critical role in this setting.

**Speaker Three: M. Fitzcharles**

**Panelists: O. Finlayson, R. Deamo Assis, R. Nemeth**

Documentation: Where Medicine Meets Law and pitfalls to avoid: We will highlight the importance of the clinical record that may be read outside of usual clinical context, the importance of prioritizing function over symptoms, common documentation pitfalls to avoid, causal statements without substantiation, and language that introduces bias.

**At the end of this session, participants will be able to:**

• Explore why the medico-legal system exists in the context of chronic pain

• Identify their role and responsibilities as treating clinicians within medico-legal processes

• Communicate and document clinical information in a way that is clinically sound and legally safe

## Concurrent Session Three

**Session Title: New horizons for the understanding and treatment of pain in Multiple Sclerosis**

**Session Chair: Bradley Kerr**

University of Alberta

**Session Abstract**

Chronic pain is a common feature of nearly every autoimmune disease. Multiple Sclerosis (MS) is one example of an autoimmune disease in which chronic pain is a significant burden for people with the disease. The prevalence of autoimmune disease in general is increasing worldwide and more specifically, Canada has some of the highest rates of MS in the world. While it was once overlooked, pain has come to be recognized as a significant feature for people living with MS and a concerted research effort has begun to better understand the underlying causes and treatment of pain in this disease. This symposium will bring together some of the leading laboratories in the field to discuss recent pre-clinical findings on the underlying mechanisms of pain in MS and a discussion of sex differences in these pathways. Speakers will present data demonstrating the contribution of neurons in the peripheral nervous system (PNS) as key drivers of pain in MS and also new therapeutic strategies aimed at targeting the PNS to treat pain in this CNS targeted disease. The symposium will also highlight exciting new data demonstrating the efficacy of a novel strategy to target TNFR2 signalling as a viable therapeutic approach to treat MS associated pain in both sexes.

**At the end of this session, participants will be able to:**

Identify novel sites and sources for pain generation in Multiple Sclerosis.

- Appreciate the specific cellular processes that lead to changes in neuronal function during autoimmune disease.

Evaluate the preclinical efficacy of novel therapeutic strategies to treat pain in MS.

**Speaker One**

**Title: Hormonal regulation of sex-specific neuroplasticity and pain in multiple sclerosis**

**Andrea Klassen**

University of Alberta

**Abstract**

Multiple sclerosis (MS) is a neuroinflammatory disease frequently accompanied by neuropathic pain, affecting up to 75% of patients. Women are more likely to develop MS and experience neuropathic pain, while men often experience a more progressive and neurodegenerative disease course, suggesting sex-specific influences on neuroplasticity and pain mechanisms. Using the experimental autoimmune encephalomyelitis model, our work investigates how gonadal hormones regulate peripheral neuroplasticity in MS-associated pain. Through the four core genotype mouse model, which separates chromosomal and hormonal contributions, we identified that female hormonal conditions promote plasticity and regeneration, whereas male hormonal conditions drive degeneration and neuronal injury. These neuroplastic changes parallel distinct inflammatory profiles in the dorsal root ganglia, revealing divergent peripheral pathways underlying pain vulnerability. Complementary studies in ovariectomized and orchiectomized mice further demonstrate that the removal of estrogen or testosterone differentially alters pain behaviours and disease outcomes, indicating that circulating hormones dynamically shape these sex-specific responses. By defining how hormones influence peripheral neuroimmune interactions, this work provides new insight into the biological basis of sex differences in pain and highlights potential avenues for sex-informed therapeutic strategies in MS.

**Speaker Two**

**Title: Eukaryotic Initiation Factor 2A Regulates Pain Hypersensitivity in Experimental Autoimmune Encephalomyelitis, a Model of Multiple Sclerosis**

**Muhammad Saad Yousuf**

University of Texas

**Abstract**

Multiple Sclerosis (MS) is an autoimmune disease of the central nervous system characterized by inflammation, demyelination, and neurodegeneration. Over 75% of MS patients experience neuropathic pain, which remains inadequately treated. We have previously shown that translational regulation through the Integrated Stress Response (ISR) contributes to pain pathogenesis. The ISR suppresses global protein synthesis via phosphorylation of eukaryotic initiation factor 2α (eIF2α), while promoting translation of stress-adaptive genes through eIF2A. We hypothesized that eIF2A-mediated translation contributes to the initiation and maintenance of pain in MS and its animal model, experimental autoimmune encephalomyelitis (EAE). Immunohistochemical analysis of post-mortem dorsal root ganglia (DRG) from MS patients revealed increased expression of phosphorylated eIF2α and eIF2A in nociceptors compared with non-MS controls. Using a constitutive eIF2A knockout (eIF2AKO) mouse, we found that loss of eIF2A did not affect disease progression or overall EAE severity but significantly attenuated mechanical and cold hypersensitivity. Bulk RNA sequencing of DRGs and spinal cords from eIF2AKO and wild-type mice at EAE onset and chronic timepoints identified distinct inflammatory gene signatures in wild-type tissues that were diminished in eIF2AKOs. These results establish eIF2A as a key translational mediator of neuropathic pain in MS. Targeting the ISR may provide a novel therapeutic avenue for alleviating pain in demyelinating diseases.

**Speaker Three**

**Title: Sex-Chromosome complement & Activin-A shape the therapeutic potential of TNFR2 activation in a model of MS and CNP**

**John Bethea**

George Washington University

**Abstract**

Multiple sclerosis (MS) is an autoimmune and neurogenerative disorder affecting the central nervous system (CNS). MS is more common in females than males (~3:1) with females more commonly developing a relapsing remitting phenotype whereas males develop a progressive disease. Neuroinflammation characterized by infiltrating peripheral leukocytes and the activation of resident microglia and astrocytes results in demyelination, axonal and synaptic damage and a complex array of clinical complications. Chronic pain occurs in approximately 75% of all MS patients is one of the more debilitating neurological complications associated with MS and unfortunately there is no effective therapy.

TNFR2 activation is a promising therapeutic strategy for autoimmune disorders such as MS and chronic neuropathic pain (CNP). This study aimed to identify mechanisms governing the sex-specific efficacy of TNFR2 activation on abrogating pain and motor disease severity in mice experiencing experimental autoimmune encephalomyelitis (EAE), a rodent model of MS. We find that the XX sex-chromosome complement is indispensable for TNFR2 mediated attenuation of EAE associated motor disease. Mice with XY chromosomes experienced exacerbated motor disease severity, associated with an elevated magnitude of neurodegeneration and demyelination. Contrasting this, we show that TNFR2 mediated alleviation of EAE induced CNP is both sex and sex-chromosome independent. However, the alleviation of CNP following TNFR2 activation across two different neuropathic pain models (EAE and chronic constriction injury) was dependent on the gonadal hormone Activin-A.

These findings highlight the importance of considering sex chromosomes and sex-independent gonadal hormones in evaluating potential sex-specific differences in drug efficacy during therapeutic development.

**Session Title: Critical Approaches to Chronic Pain Research: Insights from Research with Structurally**

**Marginalized Communities**

**Session Chair: Desmond Williams**

Person with Lived Experience

**Session Abstract**

This panel brings together projects that collectively reimagine pain research through frameworks of equity, relational accountability, and structural critique. Each presentation addresses how systems of knowledge, care, and governance shape the experience and management of chronic pain among marginalized communities. The first presentation centers Indigenous epistemologies that conceptualize pain as a relational phenomenon intertwined with Land, ceremony, and community. The second focuses on engagement with Black communities in chronic pain research, examining the social and structural determinants that have historically excluded Black Canadians from equitable participation. Using community-based participatory research, it outlines a culturally grounded framework for sustained, justice-driven partnerships. The third interrogates the bureaucratic systems that govern disability-related income support, revealing how administrative processes reproduce poverty and suffering for people living with chronic pain. Together, these papers highlight the intersections of pain, power, and policy, advancing a collective vision of research that is inclusive, community-led, and attentive to structural violence. The panel invites reflection on how culturally responsive, participatory, and critical methodologies can move pain scholarship toward systemic change and social justice. It also advances critical scholarship in chronic pain research, while elucidating the importance of such approaches for addressing structural (as opposed to biophysiological aspects of life with chronic pain. “The speakers and Chair are all members of PEPR, a national SSHRC funded Partnership concerned with promoting EDI-D in patient engagement and building capacity for critical social science approaches to pain scholarship.

**At the end of this session, participants will be able to:**

Outline structural elements of inequities with respect to chronic pain.

- Apply community-based and equity-focused principles to design more inclusive pain research engagement strategies.
- Explore how policies can cause harm and will be able to identify ways in which this amplifies and aggravates the struggles of people with chronic pain in Canada who are socioeconomically marginalized.

**Speaker One**

**Title: Weaving Indigenous Knowledge into Pain Research: Cultural Pathways to Meaningful Engagement Through Sacred Spaces**

**Vanessa Ambtman-Smith**

Western University

**Abstract**

Indigenous epistemologies conceptualize pain and nature as fundamentally interconnected, with healing occurring through reconnection to Land, ceremony, and collective wisdom. This presentation describes a culturally responsive methodology for engaging Indigenous peoples in chronic pain research, centering Traditional Healing Spaces (TH Spaces) at the Centre for Addiction and Mental Health (CAMH). This research examines Indigenous peoples’ perspectives on engagement as knowledge holders rather than research subjects, centering Indigeneity as it relates to ones’ experience of pain. The study involves four urban Indigenous communities, Adults, Youth, Two-Spirit/Queer/Trans peoples, and Elders, utilizing CAMH’s Sacred Fire and ceremonial tipi as research settings. The study examines the intersection of sacred space and engagement protocols that respect Indigenous sovereignty while recognizing pain’s multifaceted meanings, as messenger, teacher, and guide, which extend beyond Western biomedical frameworks. Employing storytelling and sharing circles as primary methodologies privileges Indigenous voices historically excluded in pain research. The four-phase methodology demonstrates how ceremonial practices establish foundations for meaningful research participation. This work advances Truth and Reconciliation Call to Action 22 by positioning TH Spaces as knowledge portals for equitable research engagement. The presentation will describe how relational accountability guides the development of community-directed research protocols, ensuring Indigenous peoples maintain sovereignty over their knowledge and experiences. This framework establishes a foundation for research partnerships that centre reciprocity over extraction, demonstrating how ceremonial practices and Indigenous epistemologies can fundamentally reshape approaches to pain research within institutional settings.

**Speaker Two**

**Title: Reconceptualising Research Engagement Through an Equity Lens: Co-creating a Community Partnership Framework with Black Canadians Living with Pain**

**Anna Hood**

University of Manchester

**Abstract**

Chronic pain disproportionately impacts racialized populations in Canada, particularly Black individuals living with conditions such as lupus and sickle cell. Despite this burden, Black communities remain underrepresented in pain research and underserved by healthcare systems. Traditional models of patient engagement often fail to account for the structural, cultural, and historical factors shaping Black Canadians’ participation. In partnership with the Promoting Engagement of Patients in Research (PEPR) initiative, our project reimagines engagement through a health equity and social justice lens. The project aims to: (1) understand what motivates Black Canadians living with pain to engage in research; (2) identify facilitators, barriers, and sustaining factors; (3) explore the intended and unintended consequences of engagement; and (4) co-create a culturally grounded partnership framework informed by lived experience. Using a community-based participatory research (CBPR) approach, the study collaborates with Black-led organizations, patient advocates, and individuals with chronic pain. Qualitative interviews, focus groups, and co-production workshops are being used to explore experiences, build trust, and co-develop engagement tools and strategies. Early findings are expected to reveal key motivators, relational dynamics, and structural barriers influencing engagement, such as the centrality of trust, cultural safety, and reciprocity in fostering authentic engagement. Emerging themes and process insights will be shared to inform framework development and iterative refinement. This work aims to generate a sustainable, community-driven partnership framework that advances equitable engagement in pain research. The process and preliminary outcomes will be presented to highlight lessons learned and next steps toward transforming engagement practices in Canada.

**Speaker Three**

**Title: Bureaucracy, Chronic Pain, and Poverty: Disability-Related Income Support as Structural Violence**

**Kathleen Rice**

McGill University

**Abstract**

This presentation will show how bureaucratic systems that scaffold disability-related income support in Canada enact structural violence against socioeconomically marginalized people living with chronic pain. Drawing on secondary analysis of data from an institutional ethnographic study (2020-2023) that focused on the “work” of living with chronic pain and socioeconomic marginalization, our team has analyzed participants’ experiences with Long-Term Disability (LTD) and Workers’ Compensation (WC) processes, ultimately revealing that navigating these bureaucratic systems can cause harm. Participants described forms that were opaque, contradictory, and inaccessible; interactions with distrustful or antagonistic caseworkers; and prolonged waiting periods that deepened financial precarity. Drawing on this data, in this presentation I present four interrelated themes: (1) the physical and emotional labour of completing extensive paperwork; (2) misalignment between bureaucratic requirements and the ambiguous nature of chronic pain; (3) bureaucracy as a source of direct and indirect harm; and (4) LTD regulations that entrench poverty. Ultimately, this presentation will show that argue that these bureaucratic practices constitute structural violence by embedding harm within institutional logics that delay and deny support, compel self-representation through deficiency, and reproduce systemic inequities.

**Session Title: Following POLARIS: Practical Guidance in Adjudicating Inclusively**

**Session Chair: Rebecca Pillai Riddell**

York University

**Session Abstract**

Over the ages, POLARIS (also known as the Star that does not walk around, Etoile Polaire, the North Star, or the Pole Star) has been used by many peoples as one way to help navigate a path forward on their journeys. Taking this inspiration, POLARIS (aka a Place for Online Learning for the Adjudication of Researchers Inclusively and Supportively) was created by scholars for scholars who are embedded within research institutions. Funded by a Canada Research Chairs EDI Stipend and York University, the goal is to help researchers navigate towards the just and inclusive adjudication of researchers for both hiring or award adjudication purposes. It is open-access and available in both French and English. POLARIS is for everyone who is called upon in their job to rank, judge, adjudicate, or provide feedback on researchers and their applications. Rather than ‘lowering the bar’, inclusive adjudication raises the bar in terms of both the quality and diversity of researchers in academia. Existing inequalities have historical roots that are deeply embedded within our research ecosystems and our personal mindsets. Rather than simply telling professors what they should do, POLARIS training incites professor to challenge their preconceived notions and find their own way. Join the Canadian Pain Society Equity, Diversity, and Inclusion Committee Co-Chairs Rebecca Pillai Riddell and Lynn Gauthier for an engaging and interactive workshop that promises to be an engaging stop on your journey to more equitable practices.

**At the end of this session, participants will be able to:**

Identify common biases in the adjudication of researchers.

Augment knowledge about best practices to inclusively adjudicate other researchers in peer review processes.

Build awareness of structures that may perpetuate bias in research systems and how to change them.

**Speaker One**

**Title: This is a skills-based workshop rather than a set of separate talks, under the title “Following POLARIS: Practical Guidance In Adjudicating Inclusively.”**

**Rebecca Pillai Riddell and Lynn Gauthier**

York University, Laval University

**Abstract**

This interactive skills-based workshop will first provide an overview of the training modules freely available at POLARIS (https://www.yorku.ca/research/project/polaris/). Using video content and audience-directed questions, the interactive knowledge and training portion will revolve upon posing three thought provoking questions:

1) Does Equity Training Even Work?

2) When should you NOT diversity your department?

3) Do Equity Initiatives Lower the Bar of Excellence?

Participants will then be presented with two annotated CV’s for a Research Chair position and will engage in lively debate over which candidate should be hired. Discussion will be guided to help participants reflect upon entrenched metrics and potential biases that make up one’s definition of the best person for the job.

**Session Title: Navigating Pain and Justice: Advocacy, Ethics, and Fairness in Medico-Legal Processes**

**Session Chair: Mary-Ann Fitzcharles**

McGill University

**Session Abstract**

Chronic pain often intersects with legal and insurance systems, yet the medico-legal dimensions of pain are rarely addressed in major conferences. This symposium brings together three complementary perspectives (clinician, medical expert evaluator, and patient/advocate) to explore how fairness, integrity, and advocacy can be maintained without compromising objectivity. Dr. Gofeld will focus on the treating clinician’s role, highlighting the importance of reliability in advocacy, responsibilities and coordination with insurers and rehabilitation professionals, and the impact of clinical documentation in the legal context. Attendees will learn how to balance therapeutic responsibilities with administrative obligations, maintain accuracy and empathy in records, and support patients navigating scrutiny during claims. Dr. Assis will examine the medical expert’s responsibilities, emphasizing impartiality, ethical frameworks, and the identification of bias from evaluators, claimants, or stakeholders. Participants will gain practical guidance on translating subjective pain into defensible conclusions, producing transparent reports, and influencing policy and clinical understanding of “invisible” pain in the medico-legal setting. Kate Nicholson, civil rights attorney and person with lived experience, will address the legal and human-rights dimensions, clarifying the distinction between impairment and disability, the impact of denied claims, and principles of equity in legal frameworks. Her presentation highlights the emotional and systemic consequences of exclusion and demonstrates how patient-centered advocacy can advance fairness and dignity. Together, the symposium provides a multi-dimensional understanding of medico-legal processes, equipping clinicians, experts, and adjudicators with tools to enhance justice, equity, and patient-centered care in chronic pain management.

**At the end of this session, participants will be able to**:

- Become familiar with the medico-legal landscape of chronic pain and the roles of clinicians, medical expert evaluators, and legal frameworks in shaping patient outcomes and systemic fairness.
- Recognize sources of bias and strategies for advocacy in clinical documentation, independent medical evaluation reporting, and legal decision-making while maintaining objectivity, rigor, and ethical integrity.
- Apply patient-centered and human-rights principles to enhance communication, fairness, and equity for individuals navigating the intersection of chronic pain and medico-legal processes.

**Speaker One**

**Title: Beyond Treatment: How Clinicians Can Advocate for Fairness in Pain and Law**

**Michael Gofeld**

University of Toronto

**Abstract**

Dr. Gofeld will discuss the difference between the clinician and the expert, exploring the role of the physician as an advocate for patients living with chronic pain who find themselves navigating the medico-legal system. He will discuss how clinicians can take a proactive role in coordinating communication among insurers, rehabilitation professionals, and other stakeholders to ensure fair and compassionate care.

The presentation will provide an overview of the medico-legal world (its structures, language, and influence in dispute and compensation cases) and examine how Independent Medical Evaluations (IMEs) can shape patients’ lives, both positively and negatively. Dr. Gofeld will highlight how physicians can advocate effectively for their patients while maintaining objectivity and professional integrity.

Key topics will include how to communicate with insurers, write medical notes that balance accuracy and empathy, and clearly describe functional limitations. The session will also distinguish between the roles and timelines of the treating clinician and the independent healthcare professional in medico-legal contexts.

Finally, Dr. Gofeld will discuss how the wording of clinical documentation can influence legal decisions and patient credibility, and how clinicians can navigate the tension between therapeutic responsibilities and administrative or legal demands. Attendees will gain practical strategies for maintaining patient advocacy through precise, compassionate documentation, and for supporting individuals who may feel mistrusted or scrutinized during medico-legal processes. Also, Dr. Gofeld will introduce the creation of the “Special Group if Interest” in the medico-legal practice supported by the Canadian Pain Society.

**Speaker Two**

**Title: Invisible Pain, Visible Bias: Rethinking Fairness in IME Practice**

**Rodrigo Deamo Assis**

Centre Intégré de Santé et Services Sociaux d’Abitibi-Témiscamingue

**Abstract**

Dr. Rodrigo Deamo Assis will examine how Independent IME experts can uphold fairness and compassion without compromising objectivity. Building on his clinical and medico-legal expertise, he will examine how experts can uphold justice and dignity by advocating through impartial and evidence-based reasoning in an adversarial environment.

The presentation will highlight the dual responsibility of the expert: to serve the court or trier-of-fact while maintaining respect for the individual being evaluated. Dr. Deamo Assis will discuss how to translate subjective experiences of pain into defensible conclusions that reflect both scientific rigor and human understanding.

A central focus will be the role of bias in the IME process: whether emerging from the evaluator, the claimant, or systemic pressures from stakeholders. Attendees will learn how to identify and mitigate these biases through awareness, reflection, and structured ethical reasoning. The session will also address how expert reports shape broader policies, legal decisions, and clinical attitudes toward “invisible” pain conditions. By applying ethical frameworks grounded in justice, transparency, and compassion, IME experts can help solidify their role as contributors to fairness, credibility, and trust within the medico-legal system.

By the end of this presentation, participants will be better equipped to recognize potential sources of bias and to conduct evaluations that balance objectivity with empathy, thereby advancing the integrity of IME practice.

**Speaker Three**

**Title: Being Seen and Heard: The Patient’s Voice in the Medico-Legal World**

**Kate Nicholson**

National Pain Advocacy Center

**Abstract**

Kate Nicholson, civil rights attorney, Executive Director of the National Pain Advocacy Center, and person with lived experience of chronic pain, will examine the intersection of law, equity, and human rights in the context of pain-related disability. Her presentation will explore how legal frameworks, particularly disability and human rights laws, define and protect individuals with chronic pain, and how discrepancies among these systems influence access to fair treatment and justice.

Ms. Nicholson will clarify the legal distinction between impairment and disability, illustrating how this differentiation shapes entitlement to benefits, workplace accommodations, and medico-legal decision-making. The speaker will describe the challenges of being repeatedly assessed, questioned, or disbelieved, and how these experiences can amplify suffering and erode the therapeutic alliance. The presentation will highlight how communication, transparency, and empathy from clinicians and experts can help restore a sense of respect and fairness, even in adversarial systems.

As a civil rights attorney and an individual directly affected by chronic pain, she will offer a unique perspective on the emotional and societal costs of exclusion within legal processes. Attendees will gain a deeper understanding of how fairness, transparency, and human dignity can be embedded into medico-legal and policy frameworks.

Ultimately, this session will invite clinicians, experts, and policymakers to view pain through a human-rights lens—advocating not for a side, but for justice, accessibility, and equity for all individuals navigating chronic pain in the legal and healthcare systems.

**Session Title: Personalization and Protocolization in the perioperative Period: Are they antithetical or can they work together?**

**Session Chair: Hance Clarke**

University of Toronto

**Session Abstract**

Although postsurgical pain is recognized as an important problem, and excellent studies around the mechanistic underpinnings of the acute to chronic pain conversion injury exist in the preclinical literature, acute and chronic postsurgical pain remains a problem for a substantial number of the 240 million patients who undergo surgery worldwide each year. Notably, surgical type is often not the lone or even a key determinant of pain trajectory. Instead, demographic, psychological and other individual-level phenotypic variables more closely align with pain severity and interference in both the short and long term, underscoring the importance of including such measures in studies of postsurgical pain. Given this insight, we pose the question: should our enhanced recovery after surgery (ERAS) protocols be based only around the surgical type? We will debate and explore how the movement towards personalized perioperative medicine intersects with protocolization of care and pose the question of whether distinct ERAS protocols for the “person type” might be a better approach, allowing a better fit of the intent, type, and invasiveness of preoperative preventive techniques.

**At the end of this session, participants will be able to**:

- Review the initial intent of Enhance Recovery After Surgery (ERAS) Pathways and highlight the history and evolution of elements that have been included in it.
- Gain insight into demographic, psychological, social, and biological factors that underlie interpatient variability in the processing of pain, and how these factors may explain differential effectiveness of the preventive interventions among individuals, and how this impacts their inclusion in ERAS protocols.
- Highlight the role of transitional pain services in the practical curation and administration of personalized elements of ERAS protocols.

**Speaker One**

**Title: Protocolization Enhances Compliance with ERAS Pathways**

**Girish Joshi**

University of Texas Southwestern Medical School

**Abstract**

This presentation will discuss the concerns of unwarranted variability and fragmented care- the intent of enhanced recovery with a focus on pain. Dr. Joshi will present data which describes the benefits of protocolization (standardization) of perioperative care and data summarizing the approaches to improve compliance. He will put forward the notion that protocolized and personalized care are complementary

Discussion/Conclusions: Clinicians working with emerging adults in substance use settings may wish to assess levels of pain, and similarly, those working with emerging adults in pain clinics may want to assess substance use behaviours and specific pain-related motives. Future research should continue to examine these relationships longitudinally.

**Speaker Two**

**Title: Shifting the focus to the Patient: Using the Biopsychosocial model to understand pain variability and detect differential efficacy of preventive interventions**

**Kristin Schreiber**

Stanford University Medical Center

**Abstract**

This presentation will review novel studies suggesting that certain preventive interventions are more impactful/effective among certain individuals having the same surgery, as well as others that employ the principle of enrichment with high-risk individuals. These types of studies may allow discernment of the particular patients that may derive benefit from a given intervention and importantly fuel an evidence base for personalization. Dr. Schrieber will demonstrate these principles through highlighting well-designed enriched studies.

**Speaker Three**

**Title: Practical Employment of Personalized Protocols in the Transitional Pain Service**

**Hance Clarke**

University of Toronto

**Abstract**

We will outline what enhanced recovery pathways simply try to cover too much, although this principal might be great with other post-surgical outcomes, there is no one size fits all when dealing with postoperative pain and ultimately the development of chronic post-surgical pain. Transitional Pain services are an excellent innovation in perioperative care which can help to phenotype individuals and deliver personalized care. Recent digital health outcomes will be presented that support the above statements

**Session Title: It’s Raining Menses**

**Session Chair**: Rachael Bosma

University of Toronto

**Speakers**: Tania Di Renna, University of Toronto

Lindsay Wolfson, Women’s College Hospital

Antje Barreveld, Harvard University

Natalie Osbourne, Pritzker School of Medicine

Endometriosis affects millions of people and remains widely misunderstood, underdiagnosed, and undertreated. This session of the Women’s Health Symposium brings together clinical leaders, pain specialists, and a person with lived experience to examine what endometriosis is, how it is experienced, and why diagnosis is often delayed.

Through a moderated, talk show–style discussion, panelists will explore the impact of diagnostic delay, the complexity of endometriosis-related pain, and the complementary roles of gynecology and pain care in management. The conversation will also address current gaps in research, clinical practice, and health system design, while highlighting opportunities to advance more equitable and integrated approaches to care.

By centering lived experience alongside clinical and research perspectives, this session aims to deepen understanding of endometriosis and underscore the importance of improving outcomes for people living with pain.

**At the end of this session, participants will be able to**:

- Describe endometriosis and its clinical presentation, including how the disease is experienced by patients and why symptom severity may not correlate with disease burden.
- Explain factors contributing to delayed diagnosis of endometriosis, including system-level barriers, bias and stigma in women’s health, and the impact of diagnostic delay on patients.
- Differentiate approaches to endometriosis management from gynecologic and pain perspectives, including indications and limitations of surgical and pain-focused treatments.
- Identify key gaps and priorities in endometriosis research, clinical care, and health system organization needed to improve outcomes for people living with endometriosis.

## Concurrent Session Four

**Session Title: Geriatric Discomfort: Is Endogenous Modulation a Function in Decline?**

**Session Chair: Magali Millecamps**

Université de Montréal

**Session Abstract**

In our society, geriatric discomfort is often perceived as a “normal” consequence of aging, and pain in older adults tends to be trivialized, as though it were an inevitable part of getting older. Yet this assumption hides a concerning reality: after the age of 65, one in two individuals suffers from chronic pain, and this proportion continues to rise with age. Such pain is frequently associated with increased comorbidities, such as anxiety and depression, progressive loss of autonomy, and a marked decline in quality of life. Scientific evidence suggests that endogenous pain modulation pathways - internal mechanisms that regulate pain signaling—are often altered in aging populations and may represent a key underlying factor in geriatric discomfort. Understanding these changes is crucial for improving pain management strategies and preserving comfort in later life. This interdisciplinary symposium will explore the role of endogenous modulation in geriatric discomfort from multiple perspectives, ranging from healthy aging to neurodegenerative diseases, in humans and animals. (1) Magali Millecamps will review the physiological basis of endogenous pain modulation, recent theories, and findings from animal models. (2) Guillaume Léonard will examine pain modulation mechanisms in humans, focusing on inhibitory (conditioned pain modulation [CPM]) and facilitatory mechanisms (temporal summation [TS]), and impact of aging. Finally, (3) Imola Mihalecz will examine the relationships between discomfort, pain modulation, and pain catastrophizing in older adults with Parkinson’s disease. Together, these contributions will provide an overview of current knowledge and emerging research on the decline of endogenous pain modulation with aging.

**At the end of this session, participants will be able to:**

Enhance understanding of the key concepts and emerging theories underlying endogenous pain modulation (ePM).

- Recognize that EPM is not limited to descending inhibitory pathways, but represents a dynamic, centrally integrated system involving cortical and subcortical networks.
- Discuss how aging affect endogenous pain modulation, and how these changes may contribute to chronic pain vulnerability.

**Speaker One**

**Title: Losing Control? Conceptual and Experimental Insights into Aging and Pain Modulation**

**Magali Millecamps**

Université de Montréal

**Abstract**

Introduction: This presentation will provide a historical overview and theoretical update on endogenous Pain Modulation (ePM), highlighting recent perspectives that emphasize the central role of cortical structures (SN-Salience Network, and PFC- Prefrontal Cortex). EPM is now understood as a dynamic system integrating both cortical and descending components. We will review behavioral models developed in rodents to study these mechanisms, examine how descending ePM changes with aging, and discuss its potential as a therapeutic target for pain management.

Methods: Mice were assessed using a DNIC-like (diffuse noxious inhibitory control) test at different life stages, under varying living conditions, and in models of chronic pain or neurodegenerative disease. The results obtained from these experiments will be compared to existing literature to identify converging or divergent patterns across studies.

Results: Depending on the evaluation method, the impact of aging on descending modulation varied in magnitude, but all results indicated clear alterations related to age, chronic pain, and neurodegeneration. These findings suggest a progressive decline in the flexibility and efficiency of inhibitory networks, potentially increasing vulnerability to persistent pain.

Discussion/Conclusions: These observations raise questions about the contribution of cortical mechanisms to age-related changes in ePM. Cortical networks may also undergo aging- or pain-induced alterations. The resilience or decline of these cortical modulatory systems could underlie the severity of emotional comorbidities such as depression or pain catastrophizing. Together, this work underscores the need for an integrated view of cortical and descending EPM in the context of aging and its emotional dimensions.

**Speaker Two**

**Title: Characterizing Individual Differences in Pain Modulation: From Normative Data to Predictive Modeling to Optimize Pain Management in Older Populations**

**Guillaume Léonard**

Université de Sherbrooke

**Abstract**

Introduction: Chronic pain, especially in older adults, severely impacts physical functioning, autonomy and quality of life. Dysfunctions in endogenous pain modulation can contribute to pain hypersensitivity and chronification, yet their integration in clinical practice remains limited despite strong potential for personalized management. We present findings from 2 complementary studies that address critical gaps: (1) establishing normative distributions for temporal summation (TS, facilitatory mechanism) and conditioned pain modulation (CPM, inhibitory mechanism), and (2) developing predictive models to estimate these profiles using clinically accessible variables.

Methods: Our analyses included 347 pain-free individuals and 108 participants with chronic pain. TS was induced via tonic heat stimulation, while CPM was evaluated using pressure pain thresholds and heat pain scores before and after a cold pressor test.

Results: Quantile regression revealed important variability in TS and CPM responses, ranging from strong inhibitory to strong facilitatory effects, including paradoxical patterns (e.g., hypoalgesia during TS and hyperalgesia during CPM). TS was influenced by age, whereas CPM remained unaffected. Predictive modeling using LASSO regression explained up to 40% of TS variance and 35% for CPM, identifying blood pressure, monoamines, sex, and pain catastrophizing as key predictors.

Discussion/Conclusion: These findings provide normative benchmarks for identifying atypical responses and demonstrate that pain modulation profiles can be partially estimated from readily available measures. While prediction error currently limits direct clinical application, this work represents a critical step toward integrating pain modulation assessment into routine care and tailoring interventions based on individual profiles, particularly for aging populations.

**Speaker Three**

**Title: The psycho-sensory characterization of pain in Parkinson’s disease**

**Imola Mihalecz**

Université de Montréal

**Abstract**

Introduction: The prevalence of chronic pain in Parkinson’s disease (PD) is twice as high as in the general population. Studies focusing solely on pathophysiological changes in the pain network have failed to explain chronic pain vulnerability in PD. We hypothesized that by including psychological factors, we will be able to better explain chronic pain transition in PD. Our study aimed to (1) characterize multimodal pain modulation mechanisms and (2) assess the psychological risk factor for chronic pain in PD.

Methods: Pain modulation was evaluated through heterotopic noxious counter-stimulation and expectation-induced pain modulation protocols, while pain catastrophizing was assessed as a psychological risk factor for chronic pain. We recruited patients with PD with and without chronic pain, patients with chronic pain without PD, and healthy individuals to explore the differences and similarities between vulnerabilities to chronic pain, whether related to PD or not.

Results: A two-factor analysis of variance was performed to explore characteristics associated with the presence of PD and/or the presence of pain. Preliminary analyses on our still-growing dataset did not show any significant differences between the groups in terms of pain modulation efficacy. However, a significant association was observed between chronic pain and higher levels of pain catastrophizing across the whole sample.

Discussion/Conclusion: Psychological factors have not received much attention in the understanding of chronic pain in PD. Our preliminary findings are consistent with the biopsychosocial model and suggest a new avenue to identify PD patients at risk of developing chronic pain.

**Session Title: Turning Intent into Action: A Call for Anti-Oppressive Approaches in Pain Research with Asian Populations**

**Session Chair: Kathryn Birnie**

University of Calgary

**Session Abstract**

The disproportionate impacts of pain among minoritized groups are increasingly recognized and supported by calls for inclusive and culturally responsive research and care. While Asian populations represent the fastest growing racial and ethnic group in Canada, they are underrepresented and understudied in pain research, rendering their pain disparities ‘unseen’. Existing research calls on the importance of positionality, partnering with communities, and intentionally attending to systems of power influencing pain. This symposium will showcase antiracist, culturally affirming, and community-based approaches in pain through research and knowledge mobilization with Asian populations. Speakers of various roles (trainee, early- and mid-career researcher, patient partner), content and methodological expertise (Asian health, community-based research, patient partnership), disciplines (psychology, nursing), and identities (immigrant, racialized, LGBTQIA+) will explore positionality, power, and inclusion while sharing novel research. Ms. Mica Marbil (she/her) will exemplify integrating positionality in intersectional research approaches using a scoping review of chronic pain among Asian diasporic youth. Dr. Samantha Louie-Poon (they/she) will illustrate how to align antiracist research aims and methods through her work using Transitional Chinese Medicine as community-care for Chinese youth experiencing pain. Jenny Lorca (she/they/siyá) will demonstrate the impact of digital storytelling as an engaging and empowering knowledge mobilization tool for people with lived and situated experience of pain. Chaired by Dr. Kathryn Birnie (she/her), a faculty member engaging in antiracism work and partnership with Asian communities and other organizations, this symposium will present ways of learning from Asian communities and unlearning colonial practices in pain research, transferable across identities, experiences and foci.

**At the end of this session, participants will be able to**:

- Identify how to integrate their unique identities, experiences, and contexts to inform anti-oppressive pain research.
- Recognize epistemic injustices that pervade Western pain research and that impact systemically marginalized populations, including Asian communities.
- Apply reflexive, culturally affirming, and community-led strategies to increase intention and impact of their own pain research.

**Speaker One**

**Title: Positioning Positionality: An Example of Integrating Experience & Context in Work with Asian Diasporic Populations**

**Mica Marbil**

University of Calgary

**Abstract**

Introduction/Aim: Positionality is crucial in anti-oppressive research. It also requires going beyond listing our identities to integrate them in our work—a practice useful for trainees, people with lived experience, and researchers of minoritized groups, but that remains minimally discussed. Drawing from Ms. Marbil’s experience as a racialized trainee conducting pain research with and about her own Asian community, this talk will invite participants to incorporate the contexts and histories that influence pain among marginalized groups, specifically Asian youth, exploring how anti-oppressive research methods can be applied.

Methods: A scoping review following JBI methodology and informed by researcher reflexivity and contexts around Asian health identified peer-reviewed studies including Asian youth with chronic pain in Western settings. Data extraction used PCC (Population, Concept, Context) and PROGRESS-PLUS (Place of residence; Race, ethnicity, culture, language; Occupation; Gender, sex; Religion; Education; Socioeconomic status; Social capital) to incorporate intersecting factors in chronic pain among Asian youth. Results are synthesized narratively, grounded in researchers’ experience and knowledge as part of the Asian diaspora.

Results: Identified studies include various pediatric Asian subpopulations. Strengths and limitations of explanations for pain disparities among Asian youth in Western settings will be presented, highlighting the role of positionality and evidence to interpret and contextualize research findings.

Discussion/Conclusions: Positionality will be demonstrated as integral to intentional, intersectional, and inclusive pain research. This talk will share findings to better understand pain experiences of Asian diasporic youth, while also imparting foundational skills to conduct anti-oppressive pain research across various populations and pain research topics.

**Speaker Two**

**Title: (Re)imagining Pain Research: Embedding a Transformative Lens with Traditional Chinese Medicine**

**Samantha Louie-Poon**

University of Alberta

**Abstract**

Introduction/Aim: Pain research is founded on Western models that often marginalize diverse understandings of health. This perpetuates injustices in pain care, particularly for communities where traditional healing practices are central. This presentation aims to reimagine the underpinnings of pain research through a standpoint grounded in Eastern Ways of Knowing (EWOK).

Methods: Situated in both lived experience (second-generation Chinese immigrant) and empirical findings, a team of East Asian researchers conducted a scoping review and critical inquiry into the philosophical assumptions of pain research. Drawing upon principles from EWOK, we analyzed key texts and scholarly interpretations to (re)imagine alternate orientations to guide pain research. We aimed to create a more inclusive research lens that validates and integrates Traditional Chinese Medicine (TCM) in pain research for Chinese youth.

Results: TCM is conceptualized simultaneously as a set of culturally affirming pain interventions and a method of community care for Chinese youth experiencing pain. Findings highlight how pain experiences for Chinese youth are embedded within the context of community, Qi (vital energy), and interconnectedness. Accordingly, pain research from EWOK broadens scientific inquiry beyond white, Western frameworks.

Discussion/Conclusions: This presentation provides a theoretical foundation for transforming pain research from a deficit-oriented model to one of hope, cultural affirmation, and community. More than a form of pain care, TCM fosters deeper, meaningful engagement with Chinese youth by honoring their traditions and cultural wisdom in research. Adopting culturally affirming lenses can thus lead to more effective and compassionate pain care that respects a diversity of knowing and being.

**Speaker Three**

**Title: Kapwa: Exploring the Power of Conversation and Storytelling Through Masakit (It Hurts)**

**Jennifer Lorca**

PainBC/Pain Canada

**Abstract**

Introduction/Aim: While a socio-relational framework of disseminating experiences of pain aligns with culturally affirming knowledge, these conceptualizations have been historically delegitimized within Western academic traditions. Such practices perpetuate the epistemic marginalization of minoritized communities. Digital storytelling (DST) offers an effective knowledge translation tool of resistance by amplifying the distinct stories of communities whose lived and situated experiences of pain have been silenced by colonial systems of knowledge dissemination.

Methods: As a platform of anti-oppressive, immersive, and relational learning, this talk will show Masakit (It Hurts), a 7-minute DST project portraying the intricate and interconnected threads of identity, culture, context, and pain. Following the viewing of Masakit (It Hurts), a facilitated discussion will invite the audience to engage with the story. This practice is rooted in kapwa: a Filipino word that calls to the shared identity and humanity in each of us, uniting us in healing through talking about pain.

Results: Masakit has been shared and viewed by people living with and without pain, situated both in and outside of the Filipinx-Canadian community, and for whom the story is a collective experience. Through a post-video facilitated discussion, participants will directly engage with Filipino experiences, stories, and concepts, immersing themselves in a culturally affirming way of understanding pain.

Discussion/Conclusions: To understand and care for pain, especially among marginalized individuals, it is necessary to intentionally engage with and amplify their voices and stories. Masakit amplifies the story of family and community, diaspora and migration, pain and discomfort, and importantly, of growth and hope.

**Session Title: Impact of ambient light on pain processing and pain management**

**Session Chair: Jason McDougall**

Dalhousie University

**Session Abstract**

Pain processing and perception are modulated by a number of environmental factors including ambient light. Light intensity, photoperiodicity, and even the wavelength of light can all alter how pain signals are transmitted and processed by the pain pathway. Light is detected by photoreceptors in the eye, relayed to the suprachiasmatic nucleus (SCN) of the hypothalamus, triggering the expression of clock genes that regulate circadian (24-hour) rhythms systemically. This rhythmicity, or lack thereof, can be a factor that impacts the experience of pain. The specific wavelength of ambient light can also influence how pain is encoded and perceived in chronic pain patients. In migraine patients, for example, visual exposure to red light can exacerbate migraine symptoms whereas green light has been shown to reduce the number and intensity of migraine attacks. We may therefore be able to use light to our advantage to “realign” the circadian dysfunction and sensory hypersensitivity that occurs in painful disorders. In this symposium, the presenters will outline how circadian rhythmicity and exposure to different wavelengths of light in our environment can influence chronic pain mechanisms leading to altered pain sensitivity. The findings from our preclinical studies have informed follow-up clinical trials which have shown the potential benefits of regulating our visual exposure to light to help alleviate the symptoms of chronic pain.

**At the end of this session, participants will be able to**:

- Introduce the influence of ambient light periodicity and wavelength on peripheral and central pain mechanisms.

Highlight the impact of circadian rhythmicity in preclinical and clinical settings.

- Share preclinical and clinical findings showing that repeated visual exposure to green light can reduce pain levels and improve quality of life in arthritis patients.

**Speaker One**

**Title: Circadian Dysfunction in Chronic Low Back Pain**

**Nader Ghasemlou and Doriana Taccardi**

Queens University

**Abstract**

Introduction: Circadian rhythmicity represents a potential contributor to fluctuations in pain intensity. Light therapy has been used to alter circadian rhythms across diseases. This study aims to phenotype to select the best candidates for bright light therapy (BLT) as a tool to “reset” circadian dysfunction and improve chronic low back pain.

Methods: Eligible participants complete: 1) the CircaHealth-CircaPain battery to collect biopsychosocial measures; 2) a 10-day e-diary Ecological Momentary Assessment (EMA), at 3 times per day (8am, 2pm, 8pm), to identify phenotypes of pain rhythmicity; 3) Dried Blood Spot (DBS) protein saver cards within 12 hours to detect molecular changes. Participants are randomized to two arms: 1) BLT for 12 weeks; or 2) treatment as usual (TAU). Measures are repeated at baseline (t0), at the end of the intervention (12 weeks after baseline; t1) and at the second follow-up 12 weeks after the completion of BLT/TAU (t2).

Results and Discussion: A pilot study on healthy individuals demonstrated that DBS can be used to detect daily rhythmic changes in proteomics; there is also potential to use this at-home blood collection tool to collect RNA. The set-up of a clinical trial that considers inter- and intra-individual differences in pain rhythmicity, including fluctuations in pain intensity and molecular changes over time will be discussed, along with challenges and opportunities faced in the process. The potential of circadian rhythm restoration in individuals with arrhythmic phenotypes as a precision medicine approach to improve chronic pain will also be discussed in the context of neuroimmune interactions.

**Speaker Two**

**Title: Ambient Green Light Reduces Osteoarthritis Pain by Engaging the Endocannainoid System**

**Jason McDougall**

Dalhousie University

**Abstract**

Introduction: The pharmacological management of osteoarthritis (OA) pain is compounded by the fact that current treatments have limited efficacy and are often associated with negative side-effects. Alternative, non-pharmacological approaches are therefore of great interest. Visual exposure to ambient green light has been found to be beneficial in chronic pain conditions such as migraine and low back pain. This study aimed to assess the analgesic effects of green light in a rodent model of OA.

Methods: Knee OA was induced in male and female Wistar rats by intra-articular injection of sodium monoiodoacetate. Following OA development, rats were exposed daily to either white light (control) or green light (experimental). Pain behaviour was tested by hindlimb weight bearing and von Frey hair mechanosensitivity. The effect of light treatment on knee nociceptor sensitivity was determined by in vivo electrophysiology. The role of the endocannabinoid system was tested by treating separate cohorts of animals with the selective CB-1 receptor antagonist AM281.

Results: Compared to white light treatment, exposure to green light significantly reduced pain behaviour in both male and female rats. Peripheral nociceptor activity, however, was unaffected by either light intervention. The analgesic effects of green light were attenuated in rats treated with the CB-1 antagonist AM281. Responses were equiefficacious in both sexes.

Conclusion/Discussion: Daily visual exposure to green light reduced joint pain by promoting the systemic release of endocannabinoids. This photobiomodulatory response occurred independently of peripheral nociceptor activity suggesting the involvement of higher centres in the pain pathway.

**Speaker Three**

**Title: Examining the Analgesic Potential of Green Light Therapy in Patients with Osteoarthritis**

**Melissa O’Brien**

Dalhousie University

**Abstract**

Introduction: Clinical trials have demonstrated that visual exposure to dim green light can help reduce acute and chronic pain. How green light therapy may affect patients with painful knee osteoarthritis (OA) has yet to be investigated.

Methods: Using a one-way crossover design, we examined the effect of green or control white light therapy in patients with moderate to severe knee OA pain. Study participants were provided with LED strips and viewed dim white light for 1-2 hours per day for 10 weeks. Following a 2-week wash-out period, participants were switched to green light treatment (wavelength = 525nm) for a further 10 weeks. Our primary outcome was changes in arthritis disability score as measured by the Western Ontario and McMaster University Arthritis Index (WOMAC) questionnaire. Secondary outcomes included patient-reported changes in pain intensity, pain disability, and patient satisfaction.

Results: Nineteen participants completed our study. Following green light therapy (GLT), average WOMAC scores significantly decreased relative to baseline measures while control white light therapy (WLT) had no significant effect. Both WLT and GLT reduced pain intensity scores; however, green light produced a greater analgesic effect. In addition, GLT reduced pain interference scores whereas WLT had no effect on this parameter. The Patient’s Global Impression of Change was significantly improved over baseline with both WLT and GLT.

Discussion/Conclusions: Our study demonstrated that daily exposure to ambient green light had a beneficial effect on OA knee pain. The mechanism responsible for this promising analgesic effect requires further investigation in a larger cohort of OA patients.

**Session Title: Cancer pain management in Canada: Towards the development of a patient-centered competency-based curriculum for Canadian healthcare professionals**

**Session Chair: Lynn Gauthier**

Université Laval

**Session Abstract**

Pain is prevalent, distressing, and disabling across the entire cancer continuum, yet it is consistently undertreated despite being recognized as a fundamental human right. Cancer pain management remains a complex, multi-level challenge, compounded by provider training gaps and evolving paradigms of cancer care that increasingly emphasize long-term survivorship. Physicians play a central role in pain management, yet little is known about how Canadian medical education prepares trainees or supports practicing clinicians to meet these demands. Addressing these gaps is critical to closing the evidence-to-practice divide and ensuring equitable patient-centered care. In this bilingual (English/French) session, Maud Bouffard will review the methodological framework for developing competency-based curricula and present findings from two studies: one mapping international cancer pain training programs and the other analyzing Québec medical faculty content, identifying gaps and proposing directions for a comprehensive competency framework. Marie-Ève Cimon and Marie Josée Hammond will share qualitative insights into Québec family physicians perceived needs for cancer pain training, highlighting important knowledge and skills gaps and attitudes influencing pain management, underscoring the need to expand this analysis nationally. Nicole Alberts will describe two studies evaluating Canadian surgery residents’ and oncologists’ cancer pain knowledge and training needs and introduce the development of a pan-Canadian initiative aimed at informing a patient-centered competency framework. Each presentation will employ a bilingual delivery format, with oral content delivered in one language and accompanying slides in the other, and Lynn Gauthier will moderate an interactive discussion in English and French, welcoming audience participation in both languages.

**At the end of this session, participants will be able to**:

- Comprehend the state of knowledge and the scope of existing cancer pain curricula and identify the objectives and content covered in the initial training programs offered by the Faculties of Medicine in Quebec.
- Identify Québec family physicians’ perceived cancer pain training needs across knowledge, skills, and attitudes competency domains and describe preferred approaches to cancer pain education.
- Evaluate pain knowledge among Canadian surgery residents and oncologists and summarize key gaps and training needs in cancer pain education.

**Speaker One**

**Title: Développement d’un curriculum pancanadien pour la gestion de la douleur cancéreuse: D’où part-on?**

**Maud Bouffard**

Research Center of the CHU de Québec-Université Laval

**Abstract**

Introduction/But: La prise en charge de la douleur cancéreuse transcende les spécialités médicales et les contextes cliniques, d’où l’importance d’outiller les médecins par le biais de programmes de formation adaptés aux réalités actuelles. Dans cette présentation, nous aborderons deux études visant 1) à cartographier les programmes de formation existants dans la littérature et 2) à analyser le contenu des programmes dispensés par les Facultés de médecine du Québec, pour 3) en faire une mise en parallèle.

Méthodes: En s’appuyant sur les étapes de Thomas (2022) et la taxonomie de Bloom pour le développement d’un programme de formation par compétences (connaissances/cognitif, habiletés/psychomoteur, attitudes/affectif) afin de s’aligner avec le référentiel CanMEDS, une analyse des besoins a permis d’identifier des différences clés entre la situation actuelle et la situation souhaitée pour orienter un futur curriculum en douleur cancéreuse.

Résultats: Une revue rapide de la littérature a recensé 82 articles, décrivant 80 programmes éducatifs s’adressant en majorité aux infirmières ou sont multidisciplinaires. 31% sont destinés aux médecins. L’ensemble des programmes ciblent des apprentissages du domaine cognitif (99%), psychomoteur (78%) et affectif (84%). Toutefois, 83% de ces programmes datent d’il y a plus de 10 ans. Dans le contexte de la formation des médecins québécois, l’analyse des programmes offerts par les Facultés de médecine révèle que le contenu lié à la douleur cancéreuse représente moins de 0,02% de tous les objectifs de cours offerts au préexternat. Ces objectifs sont reliés au domaine cognitif (67%) et psychomoteur (33%), alors qu’aucun ne touche au domaine affectif.

Discussion/Conclusion: La mise en parallèle de ces résultats mettra en lumière des lacunes dans la formation initiale des médecins et abordera les contenus couverts dans les programmes existants, qui toutefois pourraient ne plus refléter l’état des connaissances actuelles. Des pistes pour orienter le développement d’un référentiel de compétences seront proposées.

**Speaker Two**

**Title: Analyse des besoins de formation des médecins de famille en matière de gestion de la douleur liée au traitements du cancer: Entretiens menées au Québec**

**Marie-Ève Cimon & Marie Josée Hammond**

Université Laval, Patient Author

**Abstract**

Introduction/But: Près de 40 % des personnes ayant eu un cancer éprouvent des douleurs liées aux traitements reçus. Les médecins de famille (MF), acteurs clés du suivi post-traitement, rapportent un faible niveau de confiance en leurs connaissances et habiletés pour en assurer la gestion. Dans cette présentation, nous parlerons d’une analyse des besoins de formation ressentis par les MF du Québec pour la gestion de la douleur liée au cancer et à ses traitements.

Méthode: Une étude qualitative descriptive a combiné un questionnaire sociodémographique électronique (REDCap) et des entretiens individuels ou de groupe. L’analyse thématique, guidée par les dimensions du référentiel CanMEDs (savoirs, savoir-être, savoir-faire), a permis d’identifier des besoins et priorités de formation.

Résultats: Douze MF ont été recrutés en 2024-2025. Neuf ont complété le questionnaire (trois exclus, pratique hors Québec), et huit ont participé à un entretien individuel ou en dyade. Âgés en moyenne de 34,9 ans (±8,7), tous francophones, ils estimaient la formation académique reçue sur la douleur cancéreuse à 3,4 (± 4,6) heures. 38% détenaient une formation complémentaire en douleur (soins palliatifs, spécialisation en douleur). L’analyse révèle une proactivité à s’autoformer, motivée par des lacunes ressenties en termes de savoirs (pharmacologie, mesures non-pharmacologiques/douleur globale), savoir-faire (inconfort à évaluer la douleur globalement, prescrire et ajuster certains traitements pharmacologiques) et savoir-être (influence des attitudes, croyances et facteurs culturels dans la gestion de la douleur). Les MF expriment la nécessité d’accorder de l’importance à la douleur dès le début du cursus de formation initiale, puis d’offrir des modules courts, accrédités et en ligne pour renforcer l’expertise médicale en cours de pratique.

Discussion/Conclusion: Ces résultats orientent le développement de formations ciblées et accessibles pour améliorer la gestion de la douleur cancéreuse. Des pistes en termes de préférences de formation seront discutées.

**Speaker Three**

**Title: Addressing the Problem of Pediatric Cancer Pain: Insights into Canadian Physician Pain Knowledge and Training Needs**

**Nicole M. Alberts**

Concordia University

**Abstract**

Introduction: Pain is prevalent but undertreated among pediatric cancer patients and survivors. Physicians across disciplines play key roles in managing cancer-related pain, and their pain knowledge is likely one of many factors influencing cancer pain management. Despite this, little is known about medical trainees’ and practicing physicians’ pain knowledge or their pain training needs.

Methods: In two separate studies, Canadian surgery residents (N=110) and practicing oncologists (N=23) completed a sociodemographic survey and the Knowledge and Attitudes Survey Regarding Pain (KASRP) - a validated 41-item measure of pain knowledge. Oncologists also completed four open-ended questions assessing perceptions of their pain training and education needs. Descriptive statistics examined overall pain knowledge, and multiple linear regression examined resident factors (e.g., specialty) associated with higher knowledge. Responses to open-ended questions were analyzed using content analysis to identify themes related to oncologists’ pain education and training needs.

Results: Residents scored 75.1% (SD=8.6, range=43.9-95.1) on average on the KASRP - below the 80% passing score, while oncologists scored 80.1% (SD=6.7, range=66.0-95.0). Personal experience with postsurgical pain was associated with greater pain knowledge among surgery residents (β=.24, p=.01). Oncologists described several gaps in their training, including a need for more consistent opportunities and improved pharmacology and sociocultural training.

Discussion: Canadian surgery residents demonstrated pain knowledge below the level generally considered adequate, while oncologists demonstrated adequate knowledge overall. Areas of strength and gaps were identified in both groups. Directions for future research and clinical implications will be discussed.

**Session Title: The Canadian Medical Cannabis landscape: Looking to the future**

**Session Chair: Hance Clarke**

University of Toronto

**Session Abstract**

Medical Cannabis is increasingly being used for the treatment of chronic pain, yet adoption, access to educated prescribers continues to be lacking for many Canadians. This Symposium will provide an overview of real-world evidence and discuss the landscape of medical cannabis and use patterns among the Canadian public. A Canadian Medical Cannabis Clinic Trials Network has been formed, and an overview of this initiative will be provided. Finally, there will be a panel discussion and significant Q and A time.

**At the end of this session, participants will be able to:**

• Describe recent real-world evidence related to medical cannabis use and 6 month observational outcomes

• Describe the steps to recognize the presence of pain sensitization in rheumatic conditions.

• Apply a treatment strategy for the use of medical cannabis in rheumatic pain.

**Speaker One**

**Title: Medical Cannabis: Real World Evidence**

**Hance Clarke**

University of Toronto

**Abstract**

Dr. Clarke will provide an overview of longitudinal real-world results within the Canadian population. He will present the results of a cohort of patients that were enrolled into a 6 month observational study aimed to assess the impact of medical cannabis use on several domains pain, sleep, anxiety, and depression.

**Speaker Two**

**Title: My arthritis is so bad, I need something to help my pain and suffering**

**Mary-Ann Fitzcharles**

McGill University

Almost three quarters of patients with a rheumatic disease report inadequate pain control, even for those with inflammatory arthritis considered well-controlled with disease modifying agents [1]. Persistent pain is most commonly attributed to nervous system sensitization, a condition that is poorly responsive to traditional pain management strategies. Steps towards the clinical recognition of associated nociplastic pain will be discussed, and potential mechanisms for effect of cannabinoids in nociplastic pain will be examined. Dr. Fitzcharles will review the current evidence for use of cannabinoids in the clinical care of patients with rheumatic conditions, with special attention to those with inflammatory joint disease. There will be discussion of the risk/benefit ratio for the use of medical cannabis in rheumatology patients of various age groups beginning with the young adult and extending to the elderly. Finally, there will be discussion of practical steps in the routine care of patients using medical cannabis.

**Additional Panelist: Dr. Winfried Hauser**

**Session Title: Molecular and cellular drivers of pathological pain across development, sex, and Species**

**Session Chair: Michael Hildebrand**

Carlton University

**Session Abstract**

Chronic pain represents a public health crisis with a desperate need for new treatment approaches. To more effectively manage pain, the underlying molecular and cellular mechanisms that drive pathological pain need to be systematically investigated. Here, we highlight our recent work addressing under-explored aspects of processes that lead to dysfunctional pain. Dr. Beggs will highlight how early life injuries can compromise the glymphatic system, and how specific molecular changes within this system can lead to pronounced impairments in sensory, affective, and cognitive function into adulthood. Dr. Ghazisaeidi will next discuss how they uncovered a spinal pain interactome that is largely conserved across sex and species, with core signaling molecules such as Spleen Associated Tyrosine Kinase (Syk) serving as promising sex-inclusive molecular targets for pain. They have also discovered sex differences in spinal gene methylation, with IRF5-mediated microglial involvement in male neuropathic pain states only. Finally, Dr. Hildebrand will discuss how novel human spinal cord preclinical assays are being combined with new rodent measures of spontaneous pain behaviours to study how a specific subtype of excitatory glutamate receptor, GluN2D, may serve as a promising spinal pain target that is conserved across sex and species.

**At the end of this session, participants will be able to**:

- Identify how specific molecular and cellular changes in glymphatic function driven by early life pain can lead to persistent vulnerabilities into adulthood.
- Appreciate how molecular mechanisms of spinal pain can be both conserved and diverge across sexes, which has important implications for the development of sex-inclusive pain therapies.
- Understand how unique human tissue and rodent preclinical pain models and assays are being used to identify new spinal treatment targets for pain.

**Speaker One**

**Title: Early-life surgical injury disrupts adult glymphatic function: Astrovascular mechanisms linking neonatal injury to persistent CNS vulnerability**

**Simon Beggs**

UCL Institute of Child Health, Department of Developmental Neurosciences, London, UK

**Abstract**

Exposure to pain or surgery in early life produces enduring changes in nociceptive processing, stress responsivity, affective behaviour, and cognitive function. These long-term outcomes arise from activity-dependent plasticity and glial reprogramming during critical periods of brain development, yet the impact of early-life pain on brain-wide homeostatic systems has not been examined. Here, we show that a unilateral hindpaw incision in neonatal mice leads to persistent alterations in glymphatic function in the adult mouse brain.

Using tracer-based IVIS imaging and light-sheet microscopy in adult animals, we identify region-specific differences in glymphatic tracer distribution between naïve and incision groups. Molecular and histological analyses implicate astrocyte–vascular mechanisms, including altered expression of aquaporin-4 and components of the dystrophin-associated complex, alongside disruption in astrocyte endfoot coverage of cerebral vessels during early post-injury development.

These findings extend existing models of early-life pain–induced changes in the brain by identifying long-lasting impairment of glymphatic clearance as a previously unrecognized consequence of neonatal surgical injury. Disruption of astrovascular function may represent a convergent mechanism linking early-life pain to persistent sensory, affective, and cognitive vulnerabilities observed later in life.

**Speaker Two**

**Title: Sex-dependent and sex-independent gene regulation after peripheral nerve injury**

**Shahrzad Ghazisaeidi**

Center for Addiction and Mental Health (CAMH)

**Abstract**

Peripheral nerve injury (PNI) triggers widespread gene expression changes in the central nervous system, yet the mechanisms governing sex-dependent and sex-independent regulation of spinal pain pathways remain poorly defined. By integrating cross-species and cross-sex transcriptomic analyses, we identified a conserved pain interactome in the dorsal spinal cord comprising 93 transcripts consistently upregulated in both mice and rats of both sexes. Network analysis revealed Spleen Associated Tyrosine Kinase (Syk) as a central signaling node. Functional validation demonstrated that intrathecal administration of Syk inhibitors, robustly reversed pain hypersensitivity in both males and females, identifying Syk as a sex-independent therapeutic target.

In parallel, reduced representation bisulfite sequencing of the rat spinal cord revealed that peripheral nerve injury induces widespread DNA methylation changes, with multiple genomic regions showing sex-dependent differential methylation. One prominent hotspot on chromosome 12 contained a cluster of microglia-associated genes and includes P2rx4, which is required for microglia-dependent neuropathic pain in males. We found sex differences in methylation of the P2rx4 promoter at an IRF5 binding site after peripheral nerve injury, suggesting an epigenetic mechanism that may regulate IRF5-mediated microglial involvement in male neuropathic pain.

Together, these data demonstrate that peripheral nerve injury engages both sex-dependent and sex-independent mechanisms within spinal pain pathways, highlighting conserved signaling nodes alongside sex-specific regulatory programs.

**Speaker Three**

**Title: Translational models to uncover new spinal treatment targets for pain: (GluN)2D or not 2D?**

**Michael Hildebrand**

Carleton University

**Abstract**

Despite being essential mediators of pain processing, the molecular and functional properties of NMDA receptor subtypes in nociceptive dorsal horn circuits are poorly understood, especially between sexes and in humans. Given its low expression at mature brain synapses but emergent role in spinal pain signaling, the GluN2D subtype of NMDA receptor is an attractive molecular target for future therapeutic approaches.

We combined single-cell/nuclei RNA sequencing analysis with immunohistochemistry to systematically explore the expression of NMDA receptor subunits, including GluN2D, in the dorsal horn of viable spinal cord tissue from male and female rodents and human organ donors. By combining patch-clamp electrophysiological recordings with subunit-specific pharmacological antagonists, we were able to determine the relative functional contributions of these NMDA receptor subtypes. Finally, we tested the effects of a GluN2D-specific antagonist, DQP-997, on spontaneous and evoked pain measures in a rodent model of inflammatory pain.

In contrast to the mature brain where GluN2A dominates synaptic responses, we found that GluN2A, GluN2B, and GluN2D are robustly expressed and contribute to synaptic responses of dorsal horn pain-processing neurons, which was conserved across sex in both species. Surprisingly, the relative role of GluN2D in human spinal pain circuits appeared to be increased compared to rodents. In our rodent inflammatory pain model, blocking GluN2D produced a reversal of pain hypersensitivity in both sexes. Our results highlight the GluN2D subtype of NMDA receptor as a promising sex-inclusive molecular target for treating pain.

## Concurrent Session Five

**Session Title: Anatomy of Failure: Why do some patients fail to improve despite comprehensive multi- or interdisciplinary care**

**Session Chair: Angela Mailis**

University of Toronto

**Session Abstract**

Multidisciplinary and interdisciplinary chronic pain programs are widely endorsed as the gold standard for complex pain management. However, a subset of patients fails to experience meaningful improvement despite receiving comprehensive, evidence-based care. This symposium examines the characteristics, predictors, and qualitative dimensions of treatment “non-response” in chronic pain rehabilitation programs. Speaker 1 will describe the Calgary Chronic Pain Center program, one of the largest in North America, and present data from re-referred patients to identify profiles associated with poor outcomes. Physician and allied health qualitative feedback will also provide insights into potential system-level contributors. Speaker 2 will present quantitative results from a 5-year cohort of 352 interdisciplinary Pain and Wellness Program completers in Vaughan Ontario, highlighting that 23% failed to improve (20% of all women participants and 32% of men participants). Hence, sex-specific trends emerged: men demonstrated higher rates of non-response linked to maladaptive cognitive patterns, whereas women—though initially reporting higher pain and disability—showed greater overall improvement. Speaker 3 will present three in-depth qualitative case studies of non-responders from the Pain and Wellness Program, exploring psychosocial, cognitive, and contextual factors not captured by standardized assessments. Together, these presentations provide a multi-level quantitative and qualitative analysis of factors underlying failure to benefit from multiprofessional care by defining the phenotypes of patients that will not benefit from these limited resources. It also emphasizes the need for individualized and psychologically attuned approaches, leading to optimized program planning and addressing areas of need.

**At the end of this session, participants will be able to**:

- Describe and compare multidisciplinary and interdisciplinary chronic pain program models including patient admission criteria, treatment components, and program structure, as illustrated by the Calgary Chronic Pain Center and Vaughan Pain and Wellness Centre.
- Analyze patient characteristics and outcomes, by identifying phenotypes of non-responders, including sex-specific trends and psychosocial factors contributing to treatment failure.
- Formulate recommendations for improving chronic pain programs, including tailored interventions, optimized admission criteria, and the development of individualized treatment approaches to enhance patient outcomes.

**Speaker One**

**Title: Failure to Launch: Noteworthy Patient Factors from a large Multidisciplinary Pain Program**

**Kelly Shinkaruk**

University of Calgary

**Abstract**

The majority of chronic pain guidelines recommend referral to multidisciplinary pain programs for patients as early as upon initiating first line treatments. This implies that all patients will benefit from a specialized treatment approach. Unfortunately, this is not the case for all patients. In a publicly funded system, it is important to define the types of patients that will, and will not, benefit from these limited resources. Identifying these groups will lead to optimized planning for programs and guide the development of future programs to address areas of need.

During this presentation, I will outline the current program offered at the Calgary Chronic Pain Center, one of the largest multidisciplinary chronic pain programs in North America. In order to elucidate which patients fail to benefit from our program, we analyze data for patients that have been re-referred following completion of the program. Qualitative data gained from surveying physicians and allied health faculty will be presented.

Ultimately, improving our understanding of the types of patients that fail to benefit from the gold standard of care for chronic pain patients will allow key stakeholders to develop resources for these groups of patients, such as specific psychological support techniques as well as rehabilitation tools that align with their need

**Speaker Two**

**Title: Phenotype of non-responders to a comprehensive interdisciplinary community-based pain management program**

**Angela Mailis**

University of Toronto

**Abstract**

Few studies report on poor outcome from multidisciplinary and interdisciplinary chronic pain programs. These studies are notable for heterogeneity given different composition and duration programs, patient-selection criteria, outcome definitions, and definition of “failure,” resulting in variable predictors and small effect sizes. Psychological and social factors appear to frequently outscore biomedical variables. On this background, I will present data from a large cohort of 352 graduates over a 5-year period who completed a 3-month intense interdisciplinary program based in the community, with all participants receiving 60-90 hours of one-to-one personalized treatments. The talk will concentrate on those who failed to experience meaningful improvement. All patients completed detailed demographics and a standardized battery of tests at entry and the 3-month exit. Initial analysis showed that 77% of the patients were considered responders (much or very much improved) vs 23% non-responders (with minimal or no improvement). While Female to Male non-responder ratio was 1.66/1, the failure rate was much higher in male vs female participants (as 1 in 3 males versus 1 in 5 females failed to experience any meaningful change). Preliminary analysis shows that women presented with higher pain levels and greater impact on daily functioning, but experienced much higher rates of improvement, as opposed to men who tended to show more maladaptive cognitive patterns and displayed higher rates of failure. These findings suggest that sex-specific treatment approaches may be beneficial - addressing depression and functional restoration in women, and cognitive restructuring around pain beliefs and acceptance of limitations in men.

**Speaker Three**

**Title: Looking Beyond the Standardized Measures: Understanding Psychological Variables Linked to Poor Treatment Response**

**Karen Spivak**

University of Toronto

**Abstract**

At the community-based Pain and Wellness interdisciplinary pain management program, participants are offered multiple coordinated services addressing physical, emotional, cognitive and life-style issues with a personalized (in-person one-to-one) approach. In addition to Mindfulness coaching, each participant receives 12, one-to-one psychotherapy sessions incorporating psychoeducation, cognitive-behavioural therapy, and acceptance and commitment therapy, as well as Pain Reprocessing Therapy, depending on individual needs. However, even with comprehensive assessments and evidence-based care, some patients don’t progress as expected despite going through the full program. I will explore psychological factors that may help explain these outcomes through a qualitative review of clinical files. By identifying themes, such as motivation, premorbid history, and psychosocial stressors, we aim to better understand the factors influencing treatment response. These insights can help refine our selection criteria for admission to the program (given limited resources), our approach to pain patients, and enhance support for those who face greater challenges in treatment. I will further present 3 illustrative non-responder cases in depth, in an effort to unravel qualitative information not captured by standardized testing.

**Session Title: Expanding Access to Specialized Pain Care in Northern Ontario: A Two-Year Experience**

**Session Chair: Michael Gofeld**

Northern Ontario School of Medicine

**Session Abstract**

Access to specialized pain management remains a major challenge across Northern Ontario, where vast geography, workforce shortages, and funding limitations contribute to striking inequities—particularly among Indigenous, low-income, and disabled populations. Through the Northern Ontario Locum Program, our team has established a sustainable hybrid model combining telemedicine consultations via the Ontario Telemedicine Network (OTN) with monthly on-site interventional clinics at Sault Area Hospital (SAH). Patients are initially evaluated virtually with the assistance of local nurses who facilitate audiovisual setup, patient education, and care coordination. Procedures are performed during monthly 3-day hospital visits, with follow-ups conducted remotely via telephone or OTN. This model enables continuity of advanced pain care without requiring patients to travel long distances. Our group’s prospective study during the COVID-19 pandemic (Interv Pain Med, 2023) validated the clinical reliability of virtual encounters, demonstrating a 93.9% intra-observer agreement between remote and in-person visits and high patient satisfaction (median 7/7). Operational results from SAH confirm these findings, underscoring feasibility and strong institutional and community support. This symposium will share insights from Sault Ste. Marie (Dr. Gofeld, Dr. Smith) and Thunder Bay (Dr. McEwen), highlighting challenges, achievements, and innovations in regional pain care. Discussions will address integration of Indigenous healing approaches, multidisciplinary program development, and funding strategies. Lessons learned are directly applicable to scaling pain services across Canada’s rural and remote communities, ensuring equitable, sustainable, and culturally sensitive access to care.

**At the end of this session, participants will be able to**:

- Identify barriers to accessing specialized pain care among Northern Ontario’s rural, low-income, Indigenous, and disabled populations.
- Evaluate the effectiveness of hybrid service models that integrate telemedicine, hospital-based procedures, and community partnerships in improving access to pain management.
- Formulate strategies to promote equitable, culturally sensitive, and sustainable pain services through collaboration between regional hospitals, academic centers, and Indigenous health systems.

**Speaker One**

**Title: Hybrid Delivery of Interventional Pain Services: Lessons from the North**

**Michael Gofeld**

Northern Ontario School of Medicine

**Abstract**

Access to interventional pain management in Northern Ontario remains limited due to geographic isolation, workforce shortages, and restricted procedural infrastructure. To address these challenges, a hybrid model combining telemedicine consultations via the Ontario Telemedicine Network (OTN) with periodic on-site interventional clinics was established at Sault Area Hospital (SAH) under the Northern Ontario Locum Program.

Patients are initially evaluated remotely with nurse-assisted OTN sessions that include education, triage, and pre-procedure preparation. Candidates for interventions are subsequently treated during scheduled three-day on-site hospital visits, followed by telephone or virtual follow-ups. This model ensures continuity of care while minimizing patient travel burden and resource duplication.

The program builds on prior research validating the clinical reliability of virtual consultations for pain management (Interv Pain Med, 2023), which demonstrated a 93.9% intra-observer agreement between telemedicine and in-person encounters and high patient satisfaction (median 7/7). The Northern Ontario experience confirmed these findings and revealed strong institutional and community support.

Key lessons include the importance of structured collaboration between locum specialists, local staff, and administrators; the value of education for nurses and referring physicians; and the role of virtual platforms in promoting equity of access. This hybrid framework represents a scalable, evidence-based model for delivering interventional pain services to underserved, remote, and Indigenous communities across Canada.

**Speaker Two**

**Title: Building Administrative and Clinical Support for a Sustainable Model**

**Kevin Smith**

Northern Ontario School of Medicine

**Abstract**

Delivering specialized pain care in Northern Ontario requires not only clinical expertise but also coordinated administrative and institutional support. This presentation describes the operational and organizational framework that enabled the successful implementation of a hybrid interventional pain program at Sault Area Hospital (SAH) through the Northern Ontario Locum Program.

Key strategies included aligning hospital resources, developing standardized clinical pathways for referral and follow-up, and integrating telemedicine infrastructure within existing outpatient systems. The collaboration among locum specialists, local nurses, and hospital administrators ensured smooth transitions between virtual consultations and on-site procedural clinics.

Institutional engagement proved essential—administrative leaders facilitated scheduling logistics, equipment availability, and quality assurance processes, while the OTN site coordinators supported technology access for patients and clinicians. Patient satisfaction has been consistently high, and procedural efficiency improved with each cycle of service delivery.

The lessons learned regarding workflow optimization, local staff training, and the importance of relationship-building with community physicians and allied health professionals will be highlighted. The SAH experience demonstrates that administrative readiness and local leadership are critical for sustaining advanced pain services in remote regions. This model provides a template for other hospitals aiming to balance specialist outreach with community-based continuity of care in geographically isolated areas across Canada.

**Speaker Three**

**Title: Resource Limitations and Opportunities for a Multidisciplinary Expansion**

**Virginia McEwen**

Northern Ontario School of Medicine

**Abstract**

Northern Ontario faces significant challenges in providing comprehensive pain management, particularly in regions such as Thunder Bay, where access to specialized services and multidisciplinary teams remains limited. Despite these constraints, innovative local initiatives supported by targeted funding have begun to reshape the landscape of chronic pain care.

Dr. McEwen will discuss her experience developing and leading a multidisciplinary pain program designed to integrate medical, psychological, and rehabilitative approaches within a resource-limited environment. The program emphasizes collaboration among family physicians, physiotherapists, mental health practitioners, and Indigenous health partners to create a coordinated continuum of care.

Through pragmatic use of resources, education outreach, and partnership with community clinics, the Thunder Bay model demonstrates how community-driven innovation can overcome geographic and financial barriers. Early outcomes include improved patient engagement, reduced wait times, and enhanced collaboration between acute and primary care sectors.

This presentation will explore the policy and funding mechanisms that enabled program initiation, the cultural and logistical barriers encountered, and opportunities for expanding similar multidisciplinary frameworks across Northern Ontario. By focusing on local empowerment and cross-sector collaboration, this experience highlights a pathway toward equitable, sustainable, and culturally inclusive pain care for rural and remote populations.

**Session Title: Transforming Perioperative and Critical Care Pain Management: From Evidence to Implementation**

**Session Chair: Yaad Shergill**

PEPR Partnership

**Session Abstract**

Surgical recovery is a critical period for optimizing pain management, improving functional outcomes, and preventing persistent opioid use. Yet, perioperative care remains fragmented, with variability in clinical practice contributing to avoidable complications and opioid-related harms. This symposium brings together complementary strategies that are reshaping perioperative care across the surgical journey: transitional perioperative pain services, Enhanced Recovery After Surgery (ERAS) pathways, and updated Canadian national opioid prescribing recommendations. The first session will examine multidisciplinary approaches to perioperative and critical care pain management, highlighting multimodal, opioid-sparing strategies and team-based practices that improve patient outcomes, satisfaction, and recovery across surgical and intensive care settings. The second session will explore how ERAS pathways provide evidence-based, multimodal care frameworks that standardize perioperative recovery, accelerate mobilization, and reduce complications and opioid exposure across surgical settings. The third session will present an update to the Canadian national consensus recommendations on opioid prescribing at discharge, reflecting evolving evidence and implementation learnings to support safer, individualized prescribing. Together, these talks will offer an integrated roadmap for improving surgical recovery, enhancing patient experience, and advancing opioid stewardship through coordinated clinical pathways, system-level strategies, and evidence-informed guidance.

**At the end of this session, participants will be able to**:

- Describe how multidisciplinary perioperative pain models and ERAS pathways improve recovery, function, and opioid stewardship.

Identify barriers and enablers for implementation of ERAS and pain management best practices.

Apply national recommendations for prescribing pain medications at hospital

**Speaker One**

**Title: Integrating Multimodal Analgesia and Patient Experience to Redefine Perioperative and Critical Care Pain Management**

**Marie Hanna**

John Hopkins University

**Abstract**

Introduction/Aim: The management of perioperative and acute pain remains one of the most significant challenges in surgical and critical care. Despite advances in analgesic therapies, overreliance on prescription opioids has contributed to a public health crisis of dependency, addiction, and opioid-related mortality. This work aims to redefine perioperative and critical care pain management by integrating multimodal analgesia and patient experience to optimize outcomes while minimizing opioid use.

Methods: An innovative, multidisciplinary model for perioperative and acute pain management was developed at Johns Hopkins Medicine. The approach emphasizes collaboration among surgical, anesthesia, and critical care teams, with patient-centered engagement as a core principle. Data were collected from more than 4,000 surgical inpatients to assess relationships between patient satisfaction, pain outcomes, and perceptions of caregiver effort and empathy.

Results: Findings demonstrate that patient satisfaction correlates more strongly with the perception that staff made every effort to manage pain than with the degree of pain relief achieved. Implementation of multimodal, opioid-sparing strategies, including regional anesthesia, non-opioid pharmacologic agents, and individualized opioid weaning protocols, resulted in improved pain control and reduced opioid exposure.

Discussion/Conclusions: Effective acute pain management in both surgical and intensive care settings requires coordinated analgesic, sedative, and rehabilitation strategies to support recovery and minimize long-term opioid use. Sustaining these improvements depends on cultural transformation within hospitals—one that values empathy, communication, patient education, and trust as essential components of care.

**Speaker Two**

**Title: Enhanced Recovery After Surgery (ERAS): Transforming Perioperative Care Through Multimodal, Evidence-Based Pathways**

**Girish Joshi**

University of Texas Southwestern Medical School

**Abstract**

Introduction/Aim: Enhanced Recovery After Surgery (ERAS) pathways provide a comprehensive, evidence-based framework designed to optimize perioperative outcomes and reduce postoperative complications. This presentation aims to illustrate how ERAS has transformed perioperative care globally by improving pain control, minimizing opioid use, and enhancing recovery trajectories across surgical specialties.

Methods: ERAS integrates preoperative optimization, standardized intraoperative care, and proactive postoperative rehabilitation through coordinated multimodal pathways. Evidence from diverse surgical disciplines was examined to evaluate ERAS outcomes related to pain control, opioid consumption, and recovery speed.

Results: Implementation of ERAS has led to significant reductions in postoperative pain and opioid exposure, shortened hospital stays, and improved functional recovery. These results highlight the success of multimodal analgesia, early mobilization, and structured patient engagement as central features of ERAS.

Discussion/Conclusions: Despite clear evidence supporting ERAS, barriers such as practice variability, system-level resistance, and difficulty sustaining compliance remain. Strategies for addressing these include standardization of protocols, team-based implementation, and alignment with institutional quality and safety goals. ERAS thus provides a scalable foundation for advancing opioid stewardship and high-quality surgical recovery.

**Speaker Three**

**Title: Updating Canadian Consensus on Pain Management Prescribing After Surgery: Evidence, Practice, and Future Directions**

**Rachael Bosma**

Women’s College Hospital

**Abstract**

Introduction/Aim: Safe opioid prescribing at hospital discharge following elective surgery remains a key priority in reducing postoperative opioid-related harms. This session aims to present updates to the Canadian Consensus Statement for the Prescription of Pain Medication at Discharge after Elective Adult Surgery, reflecting evolving evidence and emerging best practices in surgical pain management.

Methods: The updated recommendations synthesize the latest literature and practice trends in perioperative pain and opioid prescribing. Core areas include patient education and expectation management, structured risk assessment, prioritization of non-opioid analgesics, individualized prescribing based on inpatient opioid use and recovery trajectory, and strategies for safe storage and disposal.

Results: The revised consensus provides clear, evidence-informed guidance to help prescribers reduce unnecessary opioid exposure while maintaining effective pain control. The framework emphasizes multimodal approaches, enhanced patient engagement, and integration with clinical initiatives such as Transitional Pain Services and ERAS pathways.

Discussion/Conclusions: Positioned within the broader perioperative care continuum, these updated national recommendations aim to promote safer, more consistent, and patient-centered opioid prescribing practices across Canada. Alignment with system- and policy-level actions is essential to sustain improvements in surgical recovery and opioid stewardship.

**Session Title: Advancing Pain Care for Indigenous Peoples: Lessons in Effective Partnerships between Researchers and Indigenous Communities**

**Session Chair: Lisa Richardson**

Women’s College Hospital, University of Toronto

**Session Abstract**

Chronic pain disproportionately impacts Indigenous Peoples in Canada, who experience higher rates of pain, disability, opioid use, and comorbid conditions. Indigenous communities face systemic barriers to accessing culturally safe pain care, rooted in colonial history, racism, and structural inequities. Western medical models often disregard traditional healing practices, compounding mistrust and stigma in healthcare interactions. Addressing these disparities requires collaborative approaches that integrate Indigenous knowledge, uphold data sovereignty, and advance the Truth and Reconciliation Commission’s Calls to Action.

This session will offer insights from successful models of partnerships between Indigenous and non-Indigenous researchers and Indigenous-led research, featuring presentations and a panel discussion guided by Indigenous leaders and Elders. The aim of the session is to foster dialogue between Indigenous and non-Indigenous participants to strengthen relationships, promote culturally safe practices, and catalyze future collaborations to improve pain care for Indigenous Peoples.

**At the end of this session, participants will be able to:**

• Identify effective strategies for establishing and sustaining respectful partnerships with Indigenous communities and researchers.

• Describe best practices for integrating Indigenous knowledge, ethical engagement, and data sovereignty in collaborative research initiatives.

• Recognize the importance of Indigenous-led research as a pathway towards reestablishing trust in healthcare institutions and providing culturally safe pain care.

**Speaker One**

**Title: Perioperative Journey Mapping and Pain Care: Educational Resources for Indigenous Community Members and Healthcare Providers.**

**Lisa Richardson**

Women’s College Hospital

**Abstract**

The team at Ganawishkadawe - The Centre for Wise Practices in Indigenous Health at Women’s College Hospital has been leading an all-Indigenous team of researchers and clinicians to conduct a needs assessment involving sharing circles and interviews among Indigenous patients, patient navigators, and community leaders regarding experiences of perioperative care, including pain care. The project is led by an Indigenous circle of experts and Elders and follows a patient journey mapping approach to identify barriers to high quality and culturally safe care during the peri-operative period. Following the needs assessment, resources to support Indigenous patients in navigating their peri-operative journey, including pain management, are being developed in collaboration with an Indigenous artist and person with lived experience of chronic pain. Similarly, educational resources are being developed for healthcare providers to provide training on culturally safe care during the peri-operative period. This initiative highlights the importance of Indigenous-led research as a pathway to reestablishing trust in healthcare institutions and providing culturally appropriate and safe pain care.

**Speaker Two**

**Title: Chronic Pain Experiences and Care of Indigenous Patients: Educational Resources for Indigenous Communities and Healthcare Providers**

**Miki Peer and Anna Lomanowska**

University Health Network

**Abstract**

The Transitional Pain Service, a multidisciplinary chronic pain clinic located at the Toronto General Hospital, has been engaged in an ongoing partnership with Grand Council Treaty #3 (GCT#3) and Ganawishkadawe - The Centre for Wise Practices in Indigenous Health, to co-create resources that center the experiences and needs of Indigenous people living with chronic pain. Using a strengths-based approach, sharing circles and interviews were conducted with community members living with chronic pain, healthcare providers, Elders, and Traditional Healers. Under the guidance of the GCT#3 Health Council, the knowledge gathered was used to produce trauma-informed and culturally safe chronic pain-related resources for community members and healthcare providers. This project highlights how a meaningful and respectful partnership between settler and Indigenous researchers and clinicians working with Indigenous communities can lead to tangible improvements in access to pain-related resources for communities and promote the training of healthcare providers in providing culturally safer care.

**Speaker Three**

**Title: Project ECHO: Indigenous Chronic Pain and Substance Use Series**

**Patricia Poulin**

University of Ottawa

**Abstract**

Project ECHO (Extension for Community Healthcare Outcomes) is an innovative healthcare provider peer support and education model that uses video conferencing to connect community healthcare providers with specialist teams in a “hub-and-spoke” network. Between 2022 and 2023, the first Project ECHO Indigenous Chronic Pain and Substance Use Health program was launched as a collaboration between the Ottawa Hospital Program Evaluation and Clinical Research team, St. Joseph Care Group, and N’Doo’Owe Binesi (Indigenous Health Division at St. Joseph Care Group), with input from members of the Indigenous Chronic Pain and Substance Use Indigenous Advisory Board, an Elder, and an Indigenous scholar. The program was designed for health care providers committed to improving chronic pain care in partnership with Indigenous Peoples across Canada. It was grounded in a Two-Eyed Seeing philosophy, integrating Indigenous and Western perspectives on chronic pain and substance use care. With strong Anishinaabe leadership, the program was delivered as envisioned and engaged 121 health care professionals. Key insights highlight the need to acknowledge how institutional structures shape programs and underscored the importance of culturally safe frameworks for developing future initiatives.

Panel Discussion: The panel will bring together Indigenous researchers and clinicians, Indigenous community leaders, and Elders. The focus of the panel will be to reflect on lessons learned from research partnerships with non-Indigenous teams, barriers to effective partnerships, recommendations for future partnerships, and opportunities for collaboration.

**Session Title: Innovative Technologies for the Assessment and Management of MSK Pain**

**Session Chair: Ronessa Dass**

Mc Master University

**Session Abstract**

**Aim**: This symposium will explore innovative technologies applied to innovations in delivery of pain rehabilitation.

**Methods**: Three panelists will present an overview of their cutting-edge technological approaches to MSK pain management, including: 1) three distinct virtual reality devices for persistent pain management, 2) Augmented Reality Sensorimotor Training Device (ARISE) an augmented reality system designed to improve cervical sensorimotor control, and 3) co-designed animated video education for remote delivery. Following these presentations, audience members will select to participate in one of the three small group components to obtain hands-on experience utilizing the technologies or to inquire about additional information. The session will conclude with a large group discussion, allowing the audience an opportunity for reflection and sharing of the application to their own research.

**Results**: At the conclusion session, audience members will have exposure to three new emerging technologies in MSK pain. Key themes following each session include: 1) the feasibility and implementation of technologies in MSK from research to clinical practice, 2) preliminary outcomes on the clinical utility of emerging technologies, and 3) consideration of technology-enabled solutions that support better implementation in modern pain care while addressing access, inclusivity, and system-level barriers.

**Discussion/Conclusion**: This workshop will foster cross-disciplinary learning and practical knowledge exchange.

**At the end of this session, participants will be able to:**

• Identify key barriers, priorities, and implementation challenges faced by clinicians and researchers in adopting innovative technologies for MSK pain management.

• Become familiar with available technology-based solutions, including how to access, integrate, and implement them within contemporary pain care settings.

• Recognize and exchange knowledge about additional technologies and resources relevant to MSK pain management, fostering shared learning within the pain care community.

**Speaker One**

**Title: Virtual Reality for Persistent Pain**

**Félix Fiset**

Université Laval

**Jean-Sebastien Roy**

Center for Interdisciplinary Research in Rehabilitation and Social Integration

**Abstract**

This session will introduce participants to virtual reality (VR)–based exercises for the treatment of persistent pain. The exercises are implemented in an open-source software, Large Overground Virtual Suite (LogVS), which is designed to support the development of assessment and treatment tools targeting impairments and functional limitations commonly addressed in rehabilitation.

**Aims**: Demonstrate and practice VR exercises using visual feedback alteration and distraction, designed for individuals with persistent shoulder pain.

**Methods**: After a brief overview of previous research on VR-based exercises for persistent pain, we will present the ongoing clinical research project. In this study, adults with rotator cuff–related shoulder pain participate in four VR rehabilitation sessions involving different exercise types: Bimanual or Unimanual distraction tasks and Augmented or Reduced visual feedback of upper limb movement. These VR-based exercises will then be demonstrated in small groups.

**Results**: Preliminary findings from our clinical research project will be presented focusing on the feasibility and implementation of these interventions in clinical settings.

**Discussion/Conclusion**: After the practice session, the discussion will focus on the specific benefits of VR exercises and the challenges of implementing new technology in clinical settings.

**Speaker Two**

**Title: ARISE in Practice: Augmented Reality Sensorimotor Training for Cervical Rehabilitation Across Clinic and Home Settings**

**Stevie Foglia**

McMaster University

**Abstract**

**Introduction**: This session introduces the Augmented Reality Sensorimotor Training Device (ARISE), an augmented-reality platform designed to improve cervical sensorimotor control in people with neck pain. ARISE presents 3D visual targets that users locate and track using a crosshair controlled by head and neck movement. The session aims to demonstrate how ARISE can be implemented in both in-clinic and home-based rehabilitation to improve movement accuracy, smoothness, coordination, timing, and functional range of motion.

**Methods**: After a brief evidence-informed overview, participants will engage in a hands-on workshop using 10–15 ARISE devices. In small groups, attendees will rotate through clinician and participant roles, practicing goal-directed targeting, moving-target tracking, and individualized progression of speed, task complexity, and movement range. Case-based facilitation will highlight clinical adaptation for different functional levels.

**Results**: We will present findings from McMaster University research and early clinical implementation of ARISE in individuals with neck pain, including observed improvements in cervical sensorimotor performance and practical insights related to usability and adherence. This result will be focused on in clinic use of ARISE as well as at home use, and the challenges and mitigation strategies that we have developed to ensure smooth integration of a research device into a clinical setting.

**Discussion**: The session will examine benefits and challenges of translating research-developed AR technology into real-world rehabilitation. Discussion will emphasize feasibility, clinician workflow integration, and equitable access, including how structured home use with clinician oversight may support patients facing barriers related to transportation, and access to healthcare in remote or underserved areas.

**Speaker Three**

**Title: Using co-design and AI-enhanced animation software to design interventions**

**Joy MacDermid**

Western University

**Abstract**

**Aims**:

- Illustrate how remote patient education can be enhanced by integrating, co-design, animation and live video patient education interventions.

Discuss how EDID and health literacy can be integrated in co-design

Discuss barriers to implementation and potential solutions

**Background/Methods**: We will review processes we have used to co-create different patient education remote programs, sharing the clinical and scientific benefits of a structured research focused co-design process and how differences emerged across different projects. We will illustrate an AI-supported software that accelerated animated video production and when animation versus live video was indicated. Implementation barriers and findings from pilot studies will be used to illustrate potential effects.

**Results**: In the small groups we will illustrate the different programs created to discuss pros/cons and strategies for scale-up/sharing.

**Discussion/Conclusion**: Remote patient education is a cornerstone of pain self-management. Co-design and evidence-based programs are needed to achieve 5 quality aims: better patient experience and outcomes, better clinical utility, cost-effectiveness and equity.

## Concurrent Session Six

**Session Title: Conversations about the biopsychosocial model of pain: Barriers, facilitators, and experiences from clinical practice**

**Session Chair: Cynthia Thomson**

University of the Fraser Valley

**Session Abstract**

A significant evidence-to-practice-gap remains with respect to treating pain through a biopsychosocial lens. Definitions for pain long ago shifted away from the biomedical model that associates pain with tissue damage, to a model that views pain as a dynamic exchange between our biology, thoughts, emotions, social factors, and relationships. Owing largely to societal misconceptions, many people hold strong beliefs that pain is primarily associated with structural and physical abnormalities, thus perpetuating a fear of movement leading to increased disability and lower quality of life. Further, providers cite patient expectations for biomedically focused treatments as a barrier to adhering to the clinical guidelines. Shifting public and provider understanding towards a multifactorial conceptualization of pain has potential to increase acceptance of self-management practices like education, cognitive behavioural therapy, and movement as first-line treatments. We will present recent findings that explore: 1) patient-reported views on psychosocial treatments and perspectives from pain psychology, 2) how to talk about the complexities of pain in a patient-centred way, and 3) share experiences from a program to guide providers towards a biopsychosocial treatment approach.

**At the end of this session, participants will be able to:**

Describe barriers and facilitators to applying the biopsychosocial model of pain to practice.

- Explain why sharing knowledge about the biopsychosocial model is critical to promote uptake of psychosocial strategies for pain management.
- Summarize effective knowledge translation approaches that support sustainable practice change and that may support a patient-centred dialogue about the complexities of chronic pain.

**Speaker One**

**Title: If you build it, who will come? Intentions to attend online psychosocial interventions for pain**

**Susan Holtzman**

University of British Columbia Okanagan

**Abstract**

Introduction: A biopsychosocial approach to pain care optimizes treatment outcomes. In recent years, pain education and psychosocial supports are increasingly offered online to decrease barriers to access. However, engaging with these services requires an awareness of, and openness to, a holistic approach to pain care. The current study examined intentions to engage with psychosocial services for chronic pain, with a key goal of identifying factors that may facilitate and prevent engagement with these services.

Methods: Individuals (n = 996) receiving outpatient care from a pain clinic completed a survey inquiring about physical and mental health. Participants were also asked about their likelihood of accessing online pain education and pain management programs via the clinic and barriers to doing so.

Results: Approximately three-quarters of participants did not intend to engage with the clinic’s free online pain education and support services. Notably, 20% did not think these resources would help their pain. Men were over two times more likely to endorse this belief, and this finding was not accounted for by gender differences in pain, loneliness, or mental health concerns. On the other hand, people with fibromyalgia expressed a greater interest in psychosocial supports but were more likely to report pain and fatigue as barriers to participation.

Discussion/Conclusions: Findings highlight the need for enhanced communication between clinicians and people with lived experienced with pain (PWLE) regarding the potential benefits of pain education and psychosocial supports, and for tailoring those conversations, and the services themselves, to meet the diverse needs and preferences of PWLE.

**Speaker Two**

**Title: Let’s talk about pain! Interest holder engagement to inform the co-development of a toolkit to support the introduction of the biopsychosocial model of pain**

**Cynthia Thomson**

University of the Fraser Valley

**Abstract**

Introduction: Clinical guidelines recommend treating chronic pain using a biopsychosocial model (BPSm), yet the biomedical model remains dominant and public knowledge of the BPSm is low. Many people understand that pain is complex, but to our knowledge, patient-centred ways to introduce the BPSm have not been explored. Our study series identified patient and provider recommendations to inform the co-development of an implementation bundle aimed at supporting patient-centred knowledge sharing of the BPSm of pain.

Methods: We facilitated two focus groups studies: Study 1 with people living with persistent pain (n = 21) and Study 2 with health care professionals (n = 26). Each employed reflexive thematic analysis to identify themes, followed by researcher and data triangulation.

Results: We identified several convergent themes that pertained to the current state of knowledge (or lack thereof), the predominance of the biomedical model, and the importance of listening to the individual’s story. We highlight barriers and facilitators related to knowledge delivery and knowledge reception to inform toolkit development. Interest holders described a need for both active and passive knowledge exchanges with patient-facing resources (e.g., posters, videos, pamphlets) to “plant the seeds” in combination with clinician-facing resources to support conversations with the patient.

Discussion/Conclusions: Our results confirmed knowledge gaps, identified key messages, and potential techniques to support knowledge sharing. The value of BPSm knowledge sharing was evidenced by the “hope” and “agency” patients described following the exchanges. Shifting the public understanding towards a multifactorial conceptualization of pain can increase the acceptance of self-management practices.

**Speaker Three**

**Title: Implementing the Biopsychosocial Model: Driving Innovative Pain Care Transformation in Resource-Limited Settings**

**Ahmad Qayyam**

Alberta Health Services

**Abstract**

Introduction: Chronic pain is a leading cause of disability, and allied health therapists play a key role in its management. Treating pain using a biopsychosocial approach is considered best practice for chronic pain management. This session highlights the importance of integrating the biopsychosocial model into practice particularly in resource-limited settings and demonstrates how therapists can lead scalable, evidence-informed, and patient-centered care that improves access, outcomes, and system-level impact.

Methods: We implemented an initiative that integrated the biopsychosocial model into rural public physiotherapy and occupational therapy outpatient practice to improve chronic pain care in limited-resource settings (50 providers, 20 sites). Guided by evidence-based principles from the IASP, national pain frameworks and research on rural knowledge translation, the program focused on enhancing clinical reasoning, provider confidence, and continuity of care.

Implications: Our team developed structured training, clinical tools, a triage system, and a community of practice to support ongoing learning and sustainability. Allied health leadership supported the shift in paradigm through stakeholder engagement, knowledge translation and practice innovation. Our program reinforces the role of therapists as leaders in delivering innovative, evidence-based chronic pain care, especially in rural and resource-limited settings. By embedding the biopsychosocial model into daily practice, physiotherapists and occupational therapists can advance equitable, evidence-based care while contributing to health system resilience and sustainability. The project also highlights the profession’s expanding role in health system transformation, education, and patient-centered care delivery. The session is relevant to clinicians, managers, educators, and health system leaders interested in advancing equitable, evidence-based pain care.

**Session Title: Going Deeper: Arts-based Research Approaches to Chronic Pain**

**Session Chair: Heather Noga**

University of British Columbia

**Session Abstract**

Arts-based research approaches have recently become integrated into chronic pain (CP) research. Arts-based research, in various contexts beyond the field of CP, has proven to be an effective approach for facilitating communication, building researcher-participant relationships and deepening our understanding of people’s lived experiences with illness. Arts-based research presents a unique opportunity for widespread knowledge translation and relatable content is developed by people with lived and living experience (PWLLE) for PWLLE. This symposium will focus on the rational for art-based research and its potential application in CP research specifically. We will demystify a wide range of artistic approaches including digital storytelling, photovoice and painting. We will examine the value of arts-based research, not only as a research methodology, but also for its therapeutic potential and its effectiveness as a strategy for knowledge translation. Through the eyes of PWLLE we will discuss how, at times, pursuing alternative pathways, different traditional research approaches, can provide new perspectives, enrich understanding, and promote meaningful and impactful collaborations. Finally, the panel will address limitations, challenges and considerations for involving people in CP in arts-based research practices. Chaired by H. Noga, a coordinator of arts-based research projects with over 10 years of experience, we will share a range of perspectives in a panel discussion. Our panel will include Dr. Howard, a leading researcher in arts-based methodologies and knowledge translation, K. Penfold, a PWLLE who participated in a digital storytelling project and J. Desrosiers, a trainee who incorporated art workshops into her doctoral research.

**At the end of this session, participants will be able to:**

Define art-based approaches and their potential for chronic pain research.

- Outline key aspects of the lived experience of chronic pain, with a focus on the experience of participating in an arts-based research project.

Address the strengths, limitations, challenges and opportunities of using art in research.

**Speaker One**

**Title: Arts-based research for exploring the complex and multidimensional experiences of chronic pain**

**Fuchsia Howard**

University of British Columbia

**Abstract**

Introduction: Traditional research methods (e.g., surveys, interviews, focus groups) often capture only the surface of the experiences of chronic pain and typically lead to conventional outputs like scientific publications. Arts-based research fosters co-creation of knowledge and meaningful engagement between researchers and participants, generating untapped insights into chronic pain and its lived realities. These creative approaches may be therapeutic and generate exceedingly rich findings and unique media for knowledge translation.

Methods: Our team conducted a scoping review to assess the breadth and scientific potential of arts-based research in chronic pain research. We also applied several arts-based research approaches to explore chronic pain, including Photovoice, where participants conveyed their experiences through photography and narrative reflection, and Digital Storytelling, which used script writing, images and sound to create a personal video.

Results: This symposium will provide key insights from our review of 14 studies employing arts-based methods in chronic pain research. We will share Photovoice study findings depicting the experiences of 22 people, visually illuminating experiences of life with endometriosis. Lastly, we will discuss the therapeutic and ethical implications of Digital Storytelling participation, noting that 28/36 people opted to share their story widely.

Discussion/Conclusions: Arts-based research practices are not only acceptable scientific methods of knowledge inquiry but also offer highly desirable mediums of research participation for people with chronic pain that open new pathways for understanding, healing, and connection.

**Speaker Two**

**Title: Finding Connection and Meaning Through Arts-Based Research: Reflections from a Patient Partner**

**Katherine Penfold**

Person with Lived Experience

**Abstract**

Introduction/Aim: This presentation reflects on how arts-based research can create connection and meaning for those living with chronic pain, particularly in the period after medical treatment when visibility and support often fade. Drawing from my experience as a patient partner of The University of British Columbia (UBC) following a hysterectomy, bilateral salpingo-oophorectomy, and nearly 20 years of undiagnosed endometriosis, I explore how creative and collaborative research provided space to process and contribute after the acute phase of my illness had ended.

Methods: Through my participation in arts-based workshops with UBC, I engaged in creative practices such as writing, audio, and visual storytelling. These methods supported reflection on identity, loss, and healing, offering language for experiences that clinical frameworks (or the loved ones around me) couldn’t capture.

Results: Participating in this work allows me to feel seen within the “after,” a stage where pain has lessened but the emotional and social impact remain. Collaboration with researchers and peers fosters recognition, camaraderie, and a renewed sense of agency in the desperate world of chronic pain.

Discussion/Conclusions: This personal account highlights how arts-based research can bridge medical and lived experience. While each participant’s story is unique, creative collaboration offers a powerful way to honour the ongoing process of healing and meaning-making beyond a diagnosis.

**Speaker Three**

**Title: Painting the Invisible: A Story of Decolonial and Collaborative Research Exploring Indigenous People’s Meanings of Chronic Pain**

**Joséanne Desrosiers**

Université du Québec en Abitibi-Témiscamingue

**Abstract**

Introduction: The prevalence and consequences of chronic pain are not equally distributed across populations. In Canada, Indigenous People are disproportionately affected, with an estimated 38.9% living with chronic pain, the highest prevalence among all population groups. Despite this reality, Indigenous People are often invisible in chronic pain research and face multiples barriers to healthcare access due to systemic racism, discrimination, and persistent stereotypes.

Methods: Grounded in these realities and as a part of my doctoral studies, a decolonial, collaborative research project began in spring 2021, involving partners from the Val-d’Or Indigenous Friendship Centre and academic researchers. The objective was to use art workshops to explore the meanings of chronic pain among Anicinapek and Eeyouch participants.

Results: In this session, I will describe how this art-based approach, anchored in the knowledge and priorities of Indigenous research partners, helped create a culturally relevant and safe space for sharing meanings of pain to transform research and healthcare practices.

Discussion/Conclusions: As a trainee, I will reflect on my experience of exploring a new path through arts-based research, highlighting both the benefits and challenges encountered by our collaborative team. I will also discuss how this approach supported the co-creation of concrete knowledge and tools for partners, participants, and the Centre’s community.

**Session Title: From Insight to Action: Redesigning Pain Care in the Community**

**Session Chair: Carolyn A. Harrison**

Patient Partner

**Session Abstract**

Biopsychosocial, multimodal pain care has long been recognized as the optimal approach to address the diverse impacts of chronic pain across the lifespan. Yet most people living with chronic pain do not get referred to chronic pain clinics or have long waits to access tertiary care services. Therefore, the majority of pain care takes place in community settings. Patient and provider perspectives are powerful drivers of innovation in pain care. These perspectives do more than describe problems, they offer essential evidence for redesigning care and building more responsive, equitable systems. This symposium will explore how understanding pain care from multiple levels, the patient experience, the clinician experience and system processes and information flow, can guide meaningful change. Presentations will offer multidisciplinary perspectives from three provinces (British Columbia, Alberta, and Saskatchewan) using research and clinical/health systems data and experience about the role of primary and community-based pain management. Specifically, they will highlight how patient and caregiver journey mapping can inform new care pathways, how we need to address clinician training and education, and how these insights can be mobilized into concrete practice and policy reforms. By engaging with health system leaders and advocacy organizations, we can strengthen how health care provider knowledge and system-level strategies are aligned, ensuring that clinicians across primary care are equipped to meet the complex needs of people living with pain. Together, we can move from insight to action, shaping a health system that truly responds to those it serves.

**At the end of this session, participants will be able to**:

- Interpret patient and family journey maps as a strategy to understand lived experience perspectives on navigating chronic pain care in the community and identify how these insights can inform more equitable and person-centred care pathways.
- Describe process mapping as a tool for primary care health system design that integrates patient and provider experiences with chronic pain.
- Identify common gaps in knowledge and challenges faced by providers in navigating and delivering pain care and explore approaches to enhance provider capacity to educate and support patients living with chronic pain.

**Speaker One**

**Title: Journeys Through Chronic Pain Care: Mapping Family Experiences in Community-Based Settings**

**Megan MacNeil**

University of Calgary, University of Alberta

**Abstract**

Introduction/Aim: Most youth and families living with chronic pain receive care in primary and community settings rather than specialized clinics. Understanding their journeys is essential for shaping care that is timely, equitable, and responsive. This presentation examines how journey mapping can make visible family experiences and needs, and how these insights can guide knowledge mobilization and engagement processes to strengthen primary care pathways.

Methods: Patient journey mapping was co-developed with youth with chronic pain and their caregivers across Canada (n=18) through semi-structured interviews and collaborative map validation. Qualitative analysis identified touchpoints with providers, barriers and facilitators to care, and the influence of social determinants of health such as geography, income, and school or work demands.

Results: Journey maps revealed recurring challenges in how families accessed and navigated pediatric chronic pain care in primary and community settings. Families frequently described cycles of repeated visits, diagnostic investigations, and delays in connection to biopsychosocial supports. These patterns were shaped by gaps in provider knowledge, lack of coordinated pathways, and broader structural factors. Families also described moments when supportive providers validated their experiences and improved navigation.

Discussion/Conclusions: Family journeys highlight how chronic pain care in primary and community settings is influenced by provider knowledge, care coordination, and structural barriers linked to social determinants of health. Current models of primary care often lack the team-based, interdisciplinary approaches known to support evidence-informed pain management. This presentation will demonstrate how journey mapping can generate insights to strengthen coordination, provider preparedness, and equity in community-based pain care.

**Speaker Two**

**Title: Pathways and possibilities: Process mapping for health system co-design of primary care pain management**

**Susan Tupper**

Saskatchewan Health Authority

**Abstract**

Introduction/Aims: People living with pain may receive care across multiple service lines including primary care, acute or surgical services, emergency or urgent care, physician specialists, community-based multidisciplinary providers, homecare, and tertiary pain clinics. Process mapping is a tool to improve healthcare quality, efficiency, and user experience. By engaging patients and families, community interest holders, multidisciplinary healthcare providers, and operational decision-makers in process mapping, we make visible the individual steps of pain care, barriers to workflow, wastes, and redundancies. In session two of this symposium, the Saskatchewan experience of process mapping to co-design patient navigation for chronic pain in primary care will be described.

Methods: Seven primary care health networks in Saskatchewan and their community partners were engaged in patient journey mapping and health services process mapping. Champions from these networks are engaged in working groups to co-design new tools, resources, and patient navigation processes for primary care chronic pain management.

Results: New tools identified for development include screening tools and guidelines, provincial routing maps, triage tools, pathways to guide primary care decision making, and geo-mapping of pain management resources. To demonstrate process mapping for health system co-design, symposium participants will be invited to take part in a rapid-fire interactive mapping activity.

Discussion/Conclusions: Primary care is the foundation of pain management. Health system design is required to ensure care navigation and treatment supports are in place and operational for people living with pain throughout their healthcare journey.

**Speaker Three**

**Title: Redesigning Pain Care: Building Competence, Confidence, and Connection Across Disciplines**

**Tori Etheridge**

University of British Columbia

**Abstract**

Introduction/Aim: Nearly every health care provider will encounter people living with pain, yet many continue to feel underprepared to deliver effective, evidence-informed care. This presentation shares findings from a provincial engagement initiative that explored the experiences and learning needs of clinicians working in chronic pain care across British Columbia.

Methods: Pain Care BC engaged over 100 clinicians and conducted six focus groups with 29 professionals from physiotherapy, occupational therapy, nursing, mental health, and chiropractic care. Discussions examined clinician preparedness to work in pain, access to training and mentorship, knowledge gaps, onboarding experiences, and the competencies needed for effective interdisciplinary pain care.

Results: Most pre-licensure programs offer minimal pain education, and there are currently no standardized interdisciplinary competencies to guide practice, resulting in wide variation in clinician confidence, knowledge, and quality of care across the province. Key themes from the focus group revealed notable variability in clinician preparedness, access to mentorship, and confidence in delivering pain care. Participants identified multiple barriers that limit consistency and quality of care across disciplines and practice settings

Discussion/Conclusion: By identifying these educational and systemic gaps, this initiative is informing a coordinated, province-wide effort to formalize pain care competencies and strengthen professional development. The goal is to move beyond physician-centered models toward an integrated, team-based approach that builds capacity across disciplines, creating a more connected, confident, and responsive health care system for people living with chronic pain.

**Session Title: Chronic craniofacial/orofacial pain: Perspectives from Dental Professionals and Patients**

**Session Chair: Sripriya Jayaraman**

Mount Sinai Hospital

**Session Abstract**

Healthcare professionals are no strangers to acute and chronic pain conditions; dentistry is no different. In a typical dental office, patients present for care for a wide variety of reasons. Chief among the reasons is pain from odontogenic (teeth and gums) origin. These pains are managed in a short order by manipulating teeth (like root canal treatment or extractions) and supporting structures (for instance abscess drainage by mucosal incision). Besides odontogenic pain there exist a plethora of other pain states including mucocutaneous disorders encountered in a dental office that result in acute and chronic non-odontogenic/mucocutaneous pain. Chronic orofacial pain typically requires multidisciplinary approach. This lecture takes a bird’s eye view of some of the common etiologies of orofacial pain, along with diagnostic and management considerations. A strong emphasis is placed on integrating medicine, dentistry and allied health professionals. We hope to bring it all together by listening to a person with lived experience.

**At the end of this session, participants will be able to:**

Familiarize themselves with the complex nature of orofacial and craniofacial facial pain.

Outline the role of oral and maxillofacial surgeons.

- Appreciate how social determinants of health can impact pain patients’ experiences of pain and their ability to access resources.

**Title: Potpourri of Orofacial Pains**

**Speaker One**

**Sripriya Jayaraman**

Mount Sinai Hospital

**Abstract**

Toothache or even face pain conjures up visions of painful root canal treatments and/ or extractions. But, what if the pain felt in the tooth is caused by sources outside the dento-alveolar structures? This lecture takes a panoramic view of non odontogenic causes of teeth/face pain with a particular stress on integrating dentistry with medicine and allied health professionals.

**At the end of this presentation, participants will be able to:**

Elucidate the different causes of orofacial pain

Clarify the roles of dental and medical professionals in treating these complex conditions

Foster interprofessional communication and collaboration for better patient outcomes

**Title: Surgical Management in Temporomandibular Pain**

**Speaker Two**

**Ross Linker**

Private Practice

**Abstract**

Temporomandibular joint (TMJ) disorders are a broad category of chronic pain conditions affecting millions of people worldwide. Management of these patients’ pain symptoms often requires multidisciplinary integration between physicians, dentists and other healthcare professionals. Within the scope of treatment, oral and maxillofacial surgeons have a distinct role. Oral surgeons can perform a range of procedures for treatment of temporomandibular dysfunction ranging from intra-articular steroid injections to total joint replacements. Furthermore, they can help distinguish temporomandibular pain from pathological mimics. In this lecture, I will discuss temporomandibular arthrocentesis and its role in treating temporomandibular dysfunction. I will also talk about other surgical treatments for more complex TMJ pathologies. Finally, I will briefly address intraoral pathology and the oral surgeon’s role in distinguishing these lesions from TMJ conditions.

**At the end of this presentation, participants will be able to:**

Recognize the role of oral surgeons in management of temporomandibular pain

Know several surgical options for management of temporomandibular pain

Understand that pain in the TMJ may be caused by other intra oral issues

**Title: Chronic orofacial pain: a patient’s journey in navigating this complex world**

**Speaker Three**

**Chloe Foisy-Marquis**

Person with Lived Experience

**Abstract**

My entry into this world began three years ago with a nagging, growing pain in my jaw and neck area. Within 6 months I was completely incapacitated, unable to work and in too much pain to perform basic tasks.

I sought out several public and private treatment options and was eventually referred to oral and maxillofacial surgery service for pain alleviation. This took me on a years-long journey from diagnosis, to coping, to pain management modalities. This talk offers a view of my ongoing journey into this complicated world navigating different professionals, therapies, hopes and fears. My reflections are informed by my professional experience as a social worker however this talk is rooted in my experience as a patient navigating orofacial pain resources. The talk will also include a glimpse about my privileges and lack thereof along with some recommendations for better patient outcomes.

**At the end of this presentation, participants will be able to:**

Appreciate the intricate world of orofacial/craniofacial pain from a patient point of view

Better understand the need for inter professional collaboration

Receive suggestions for better outcomes from a patient’s standpoint

**Session Title: Percutaneous Epidural Adhesiolysis for Chronic Low Back and Lumbosacral Radicular Pain: Comparative Effectiveness of Conventional Catheter Technique Versus Balloon Assisted Epidural Decompression. The Evolving Role of Patient Cloning AI and Immersive Digital Twin for Spine Interventions.**

**Session Chair: Yuvaraj Kotteeswaran**

NOSM University

**Session Abstract**

Chronic refractory low back pain with or without lower extremity pain that does not resolve after conservative therapy or even surgical treatment can present a therapeutic dilemma with limited options for proper management. Low back and lower extremity pain recalcitrant to conservative management and epidural injections may be secondary to post surgery syndrome, spinal stenosis, and disc herniation. Disc herniation and spinal stenosis are often managed with surgical interventions and post-surgery syndrome may also be managed with repeat surgical interventions or implantable therapies. However, for those patients who are not responsive to or candidates for surgical interventions and/or have not adequately responded to epidural injections, percutaneous adhesiolysis may be an option. Epidural steroid injections have been used extensively in managing low back and lower extremity pain. While results of studies of epidural injections continue to be debated and differ, the proportion of patients who failed to respond to epidural steroid injections are candidates for percutaneous epidural adhesiolysis. Causes of chronic radicular pain include mechanical compression of nerve roots, as well as different proinflammatory substances that trigger ectopic neuron firing. The mechanism described in percutaneous adhesiolysis is the combined effect of local lavage of proinflammatory cytokines, reduction of swelling, lysis of adhesions, desensitization and modification of neuromodulation, and local anesthesia. The presence of epidural adhesions may be diagnosed with magnetic resonance imaging (MRI), followed by epidurography based on filling defects. These filling defects by epidurography are minimized in size after successfully performing epidural lysis of adhesions. Conventional epidural adhesiolysis techniques are broadly classified into chemical adhesiolysis using hypertonic saline and mechanical adhesiolysis using steerable catheters. Although these approaches often yield short-term analgesic benefits, functional improvement has generally been limited. Spinal epidural balloon decompression was developed to overcome this challenge in treating spinal stenosis and post lumbar surgery syndrome. Balloon decompression can achieve more extensive adhesiolysis by expanding the marginal space surrounding the affected nerve root, potentially achieving superior neural decompression, improved walking tolerance, enhanced functional recovery, and reduced need for repeat procedures in selected patient populations. The impact of these therapies including indication and benefits will be addressed at the symposium along with the implications for cost and adverse events.

In parallel, the session will explore the transformative potential of Patient Cloning AI and Immersive Digital Twin for spine intervention. Current image-guided procedures rely primarily on two-dimensional fluoroscopic visualization. However, these approaches present inherent limitations in difficult scenarios, including the misalignment between the operator’s hand movements and the direction of visual attention toward the monitor, as well as difficulties in achieving an intuitive three-dimensional understanding of internal anatomy. To address these challenges, we present newer techniques and strategies for developing artificial intelligence models and demonstrate their application to volumetric medical imaging in spine intervention. The integration of patient cloning AI and immersive digital twins represents a frontier in precision interventional spine care.

**At the end of this session, participants will be able to:**

• Describe the pathophysiological mechanisms underlying epidural fibrosis and chronic low back and lower extremity pain.

• Review of literature on the efficacy and complications of percutaneous epidural adhesiolysis in managing chronic low back and lumbosacral radicular pain.

• Review of clinical experience of the efficacy, durability of response, and complication of spinal epidural balloon decompression.

**Title: Spinal Epidural Balloon Decompression and Adhesiolysis**

**Speaker One**

**Jin-Woo Shin**

University of Ulsan and Asan Medical Center

**Abstract**

Balloon decompression and adhesiolysis is an advanced interventional technique designed to overcome the limitations of conventional epidural nerve blocks and traditional neuroplasty in patients with severe epidural adhesions or lumbar foraminal stenosis. Standard nonsurgical treatments often provide only short-term relief due to mechanical barriers and impaired epidural drug distribution.

In a randomized controlled trial of patients with intractable lumbar foraminal stenosis unresponsive to transforaminal epidural steroid injections, balloon dilatation combined with steroid injection produced significantly greater pain reduction and functional improvement than catheter insertion alone. Clinical benefits were sustained for 3–4 months, and 18.8% of patients maintained more than 50% pain relief for over one year.

Three-dimensional contrast analysis demonstrated significant expansion of the foraminal marginal space after balloon dilatation, supporting its mechanical decompressive mechanism. Based on these findings, the ZiNeu balloon-inflatable neuroplasty catheter was developed to enhance mechanical adhesiolysis while minimizing neural injury and enabling targeted drug delivery. Since 2013, 18 SCI(E)-indexed publications have supported this technique. The procedure is currently used in Singapore, Australia, Thailand, Italy, Malaysia, and Hong Kong, and its international adoption continues to expand.

This lecture will address clinical indications, technical considerations, and practical applications in refractory spinal disorders.

**Title: Patient Cloning AI and Immersive Digital Twin for Spine Interventions.**

**Speaker Two**

**Jae Chul Koh**

Korea University College of Medicine.

**Abstract**

This lecture will be delivered from within an immersive extended reality (XR) environment rather than a conventional presentation setting. Instead of viewing static slides, the audience will observe a live demonstration in which I explore a digital clinical space and performing AI-generated pain procedures in real time.

Pain procedures require precise instrument placement guided by imaging modalities such as fluoroscopy and ultrasound. However, two-dimensional visualization limits intuitive three-dimensional understanding and demands extensive operator experience.

To address these challenges, I combined my background in pain medicine and computer science to develop AI- and XR-based solutions. First, I present a Patient Cloning AI system that automatically extracts and segments anatomical structures from CT and MRI volumetric data, transforming radiodensity and signal-based datasets into semantically meaningful anatomical models.

Second, I introduce an immersive digital twin platform that enables one-click patient cloning and real-time procedural simulation. Within this environment, vascular puncture produces simulated bleeding, spinal access reveals cerebrospinal fluid, and intra-procedural X-ray images are dynamically generated according to instrument position.

This session will demonstrate how AI-driven volumetric modeling and immersive visualization can expand the future of training and procedural practice in pain medicine.

**Session Title: The Evolution of Transitional Pain Services – 10 Years in Review**

**Session Chair: Max Slepian**

University of Toronto

**Session Abstract**

Chronic Post Surgical Pain accounts for one quarter of patients presenting to chronic pain clinics. Patients who develop chronic post-surgical pain are at increased risk for persistent opioid use. Ontario data demonstrates that 50% of patients are discharged with opioid analgesics following major surgery and 3% of previously opioid-naïve patients continue to take these medications 6 months later. Significant gaps in the continuity of care after major surgery are responsible for unrelieved pain, hospital re-admissions, and ongoing opioid use among complex pain patients discharged without appropriate follow-up plans or care, and without pain specialists able to manage their postsurgical pain and/or successfully wean them from opioid medications. Transitional Pain Services have now been adopted in several major Canadian centres and internationally in the United States, Norway, UK, and Australia.

**At the end of this session, participants will be able to:**

• Provide an overview of the evolution of Transitional Pain Services over the past 10 years.

• Gain an appreciation of the PWLE perspective who manage their complex pain, mental health and sometimes, substance use.

• Outline the differences between the US and Canadian models and experience a glimpse into the future of Transitional Pain Programs.

**Title: The Experience of being cared for by a Transitional Pain Service**

**Speaker One**

**Graham Lord**

Person With Lived Experience

**Abstract**

Mr. Graham Lord will describe his person journey follow a major motor vehicle accident. Mr. Lord had brachial plexus avulsions of his C5, 6, 7 & 8 nerve roots, a fractured radius and fractured vertebrae. He will tell an unfortunately common story of entering into an overreliance on medications and substances in general. He was desperate and he will then describe when his trajectory started to change for him upon the intersection with the Toronto General Hospital Transitional Pain Program. His story is one of hope and triumph.

**Title: The Evolution of Transitional Pain Services: The American Perspective**

**Speaker Two**

**Marie Hanna**

Johns Hopkins Hospital

**Abstract**

Dr. Hanna will discuss the evolution of Transitional Pain Services and describe her successful creation of her perioperative pain program at Johns Hopkins. She will present data looking at both pain and opioid related outcomes. Dr Hanna perioperative transitional pain program has borne witness to glimmers of hope amidst the current opioid epidemic. Every patient in the program represents a success story in weaning opioids even in the most stressful period. These programs effectively reduced opioid usage without negatively influencing patient-reported outcomes, such as physical pain score and health-related quality of life

**Title: Transitional Pain Services: The Canadian Perspective and next steps**

**Max Slepian**

**Speaker Three**

University of Toronto

**Abstract**

Dr. Slepian will review the evidence for the efficacy of Transitional Pain Services in reducing opioid use in the short and long term after a variety of surgical procedures. He will also describe recent evidence from the TGH-TPS for novel risk factors for CPSP and how these may impact TPS effectiveness. A discussion regarding the integration of digital health technologies and the use of big data to improve outcomes in the years ahead, in addition to recommendations for future research related to the prevention and management of CPSP will be brought forward for discussion.

